# Design of Potent Mannose‐6‐Phosphate Derivatives as Ligands for CI‐M6P/IGF2R Using Fluorescence Polarization Assay

**DOI:** 10.1002/chem.202500973

**Published:** 2025-05-20

**Authors:** Lucie Mrázková, Klaudia Hladoníková, Barbora Toncarová, Michal Fischer, Jakub Zýka, Jaroslav Kozák, Michal Kráľ, Milan Kožíšek, Jiří Jiráček, Jakub Kaminský, Kamil Parkan, Lenka Žáková

**Affiliations:** ^1^ Institute of Organic Chemistry and Biochemistry of the Czech Academy of Sciences Flemingovo náměstí 542/2 Prague 160 00 Czech Republic; ^2^ The Department of Chemistry of Natural Compounds University of Chemistry and Technology Prague Technická 5 Prague 166 28 Czech Republic; ^3^ Department of Cell Biology, Faculty of Science Charles University Viničná 7 Prague 128 00 Czech Republic

**Keywords:** fluorescence polarization assay, IGF2, ligand binding, Mannose‐6‐phosphate, receptor

## Abstract

The cation‐independent mannose‐6‐phosphate/IGF2 receptor (CI‐M6P/IGF2R) plays a crucial role in transporting lysosomal enzymes and other ligands. In this study, we designed and synthesized novel stable mannose‐6‐phosphate (M6P) derivatives to enhance their affinity for CI‐M6P/IGF2R. To evaluate the binding potency, we employed a sensitive and cost‐effective fluorescence polarization assay, enabling rapid quantification of receptor–ligand interactions in solution. The tested compounds included di‐, tri‐, and penta‐M6P peptides along with various M6P‐derived small molecules featuring phosphate isosteres or other functional modifications. Our findings indicate that ligands bearing multiple M6P moieties exhibit significantly higher receptor affinities than monomeric compounds and that phosphonate groups may serve as a more stable and potent alternative to native M6P. Computational modeling of ligand interactions with the CI‐M6P/IGF2R domains further elucidated the binding mechanisms, offering new directions for the development of more effective ligands. This study advances the design of therapeutic strategies that leverage CI‐M6P/IGF2R for targeted biomolecule delivery to lysosomes, thereby opening new possibilities for biomedical applications.

## Graphical abstract

We designed and synthesized novel mannose‐6‐phosphate (M6P) derivatives to enhance their binding affinity for the CI‐M6P/IGF2 receptor, which plays a key role in lysosomal targeting. Using a fluorescence polarization assay, we evaluated di‐, tri‐, and penta‐M6P peptides and modified M6P analogs. Our results show that multivalent ligands significantly improve receptor binding, with phosphonate groups emerging as a promising stable alternative to native M6P

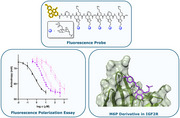

## 1 Introduction

The cation‐independent mannose‐6‐phosphate/IGF2 receptor (CI‐M6P/IGF2R) plays a pivotal role as the primary transporter of mannose‐6‐phosphate (M6P)‐bearing proteins to lysosomes. This 300 kDa multifunctional transmembrane glycoprotein is ubiquitously expressed across the animal kingdom.^[^ ^1^ ^]^ Beyond M6P‐bearing proteins, CI‐M6P/IGF2R binds a range of other ligands, including insulin‐like growth factor 2 (IGF2), urokinase plasminogen activator receptor (uPAR), plasminogen, and retinoic acid.^[^ ^2^ ^]^ The core function of the receptor is to mediate the delivery of specific proteins to the endosomal–lysosomal system for degradation. Dysregulation of this lysosomal degradation pathway can lead to various pathological conditions, including Fabry disease, Pompe disease, and MPS‐I diseases caused by lysosomal enzyme deficiencies.^[^ ^2^ ^]^ Additionally, insufficient clearance of IGF2 from plasma by CI‐M6P/IGF2R can contribute to oncogenesis, while impaired regulation of plasminogen may disrupt fibrinolysis.^[^ ^3^ ^]^

**Figure 1:**
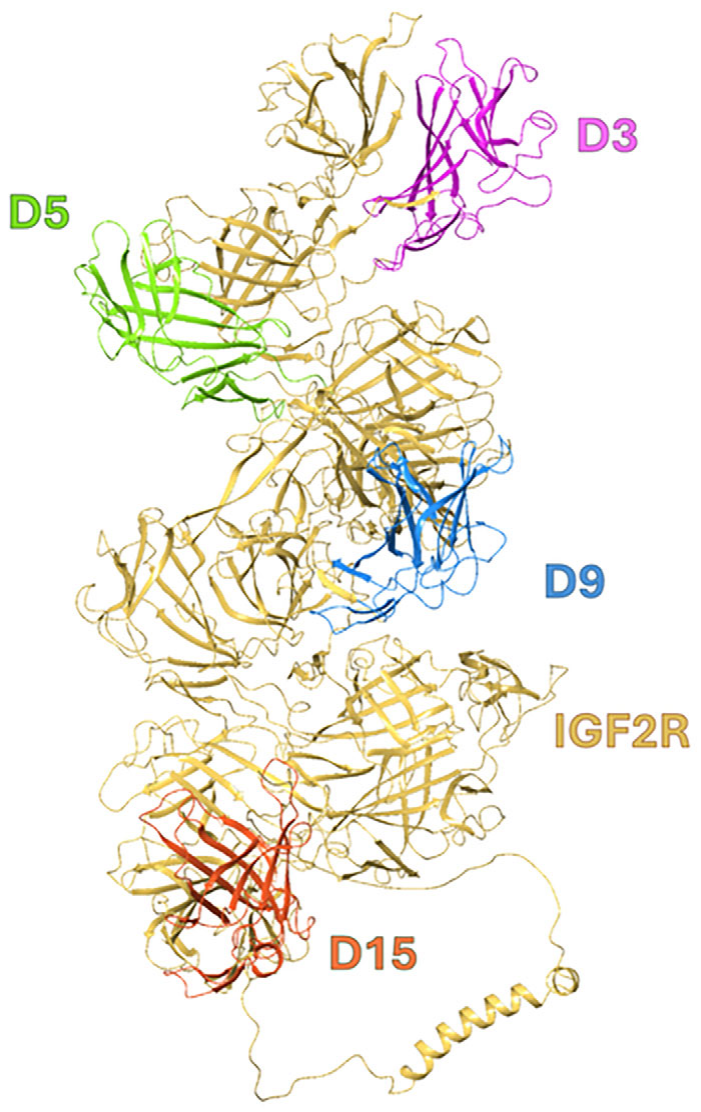
Model structure of the human CI‐M6P/IGF2 receptor (AlphaFold structure ID: AF‐P11717‐F1‐v4). The extracellular portion of the receptor consists of 15 homologous domains, with four domains identified as mannose 6‐phosphate (M6P) ligand‐binding sites. Domains 3 (D3) and 9 (D9) function as high‐affinity binding sites for M6P ligands, whereas domains 5 (D5) and 15 (D15) have been characterized as low‐affinity binding sites. Ligand binding induces extensive conformational changes in the receptor structure, which may influence the binding of additional ligands.^[^ ^6^ ^]^

The extracellular region of CI‐M6P/IGF2R comprises 15 contiguous repeat domains with a high sequence homology (14%–28% identity) (Figure 1). The receptor also features a short 23‐amino‐acid transmembrane domain and a 164‐amino‐acid intracellular domain that lacks catalytic activity but contributes to the formation of clathrin‐coated early endosomal vesicles.^[^ ^4^ ^]^ Each extracellular domain, approximately 147 amino acids in length, forms an antiparallel β‐sheet stabilized by three to four disulfide bonds, creating a flattened β‐structure.^[^ ^5^ ^]^ Ligand‐binding studies and mutational analyses have identified critical residues within domains 3, 5, 9, and 15 that are essential for M6P binding.^[^ ^6^] Despite their structural similarity, these domains exhibit distinct binding affinities for M6P‐bearing ligands: domains 3 and 9 show high affinity, domain 5 shows moderate affinity, and domain 15 shows very low affinity.^[^ ^7^ ^]^ IGF2 binds specifically to domain 11 with additional contributions from the fibronectin‐like domain in repeat 13.^[^ ^8^ ^]^

Following the vesicular transport of cargo to the lysosome, the acidic pH environment induces ligand release, enabling degradation while allowing the receptor to be recycled for subsequent use. Only approximately 10% of CI‐M6P/IGF2R resides on the plasma membrane, where it binds extracellular ligands and IGF2. The remaining majority is intracellular, facilitating the trafficking of newly synthesized hydrolases from the Golgi to the lysosomes.^[^ ^9^ ^]^ A major therapeutic application of M6P derivatives is in the treatment of lysosomal storage disorders, particularly through enzyme replacement therapy (ERT).^[^ ^10^ ^]^ In this strategy, recombinant enzymes are delivered to lysosomes via endocytosis, mediated by the interaction of their M6P residues with CI‐M6P/IGF2R on the cell surface.^[^ ^11^ ^]^ Another emerging application, lysosome‐targeting chimeras (LYTAC), is the targeted delivery of selected proteins to endosomes for degradation. One example of LYTAC is the conjugation of a specific antibody with M6Ps. The resulting construct can interact with the target protein via the antibody and with CI‐M6P/IGF2R through its saccharide moiety. This interaction induces endocytosis of the complex, ultimately leading to the degradation of the target protein in lysosomes.^[^ ^12^ ^]^ These examples underscore the critical role of the receptor as a multifunctional transmembrane transporter for diverse ligands, positioning it as an attractive target for numerous clinical applications, and highlight the importance of new detailed studies of receptor interaction with M6P ligands and the development of new, more potent, and stable derivatives.

To investigate M6P interaction with CI‐M6P/IGF2R, a simple and sensitive binding assay is essential. Such assays typically use ligands labeled with either radioactive atoms or fluorescent groups. Several systems have been described for detecting the binding of M6P‐bearing ligands to CI‐M6P/IGF2R. Pioneering studies from the 1970s to the early 1990s established the biosynthesis of M6P‐proteins and their interactions with CI‐M6P/IGF2R, revealing that effective binding requires at least two terminal M6P residues on a carbohydrate dendrimer linked to proteins via asparagine.^[^ ^13^ ^]^ Glycoproteins with multiple M6P residues exhibit a high nanomolar affinity for CI‐M6P/IGF2R, whereas monomeric M6P binds weakly (K_d_ ∼7 µM). Christensen et al.^[^ ^14^ ^]^ synthesized tripeptides with two M6P‐containing disaccharides and determined their submicromolar binding affinities using ELISA assays. Subsequent studies have shown that altering the peptide scaffold, such as increasing the distance between the M6P units or introducing cyclization, reduces potency. ^[^ ^15^ ^]^ Later, Hoogendoorn et al.^[^ ^16^ ^]^ designed peptides with six flexible M6P moieties for labeling cysteine cathepsins, whereas Berkowitz et al.^[^ ^17^ ^]^ used radioligand binding assays to measure M6P affinities and found lower binding for M6P monomers than for 6‐phosphonomethyl mannose dimers. These findings underscore the importance of multivalency and structural spacing for optimizing CI‐M6P/IGF2R binding.

A key goal in this field is the development of stable and potent synthetic M6P analogues to overcome the inherent instability of native M6P in the human serum. These analogues have the potential to enhance binding affinity to CI‐M6P/IGF2R, thereby improving the effectiveness of therapeutic interventions. To achieve this, we first employed a simple, cost‐effective, and sufficiently sensitive assay to rapidly determine the binding affinities of various synthetic or natural ligands to the M6P binding sites of CI‐M6P/IGF2R. Next, we investigated the binding affinities of a series of di‐, tri‐, and penta‐mannosylated peptides of varying lengths, each bearing a stable 6‐phosphonomethyl moiety on the mannose ring. Finally, we compared the potency of alternative functional groups on the mannose ring, evaluating options such as phosphate, phosphonate, tetrazole, and carboxyl groups.

## 2 Results and Discussion

### 2.1 Peptide Synthesis and Modifications

The syntheses of all precursor peptides and target glycopeptides **1**–**6** (Figure 2) are described in detail in the Supporting Information, which also includes the HPLC traces and MS spectra (Figures S3–S14).

**Figure 2:**
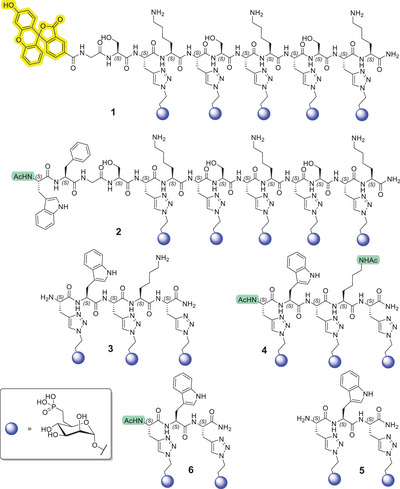
Target glycopeptides **1**–**6** bearing the 6‐phosphonomethyl mannose moiety (blue spheres). The fluorescein group is shown in yellow and *N*‐terminal acetylation is shown in green.

Briefly, all precursor peptides were synthesized via solid‐phase peptide synthesis (SPPS) on Fmoc‐Rink MBHA Amide resin (low loading, 0.42 mmol/g) using a Spyder Mark IV Multiple Peptide Synthesizer (European Patent Application EP17206537.7), developed at the Development Center of the Institute of Organic Chemistry and Biochemistry in Prague. Each amino acid was coupled twice: first, 5 equivalents of the amino acid (0.4 M) with HBTU/HOBt/DIPEA in DMF, followed by a second coupling with 7 equivalents of the amino acid using DIC/HOBt in DMF, with each coupling step lasting 75 minutes. The Fmoc groups were removed using piperidine in DMF. The peptides were cleaved from the resin using 90% TFA with scavengers, precipitated in diethyl ether, purified, and analyzed by HPLC. Their identities were confirmed by MS.

**Scheme 1:**
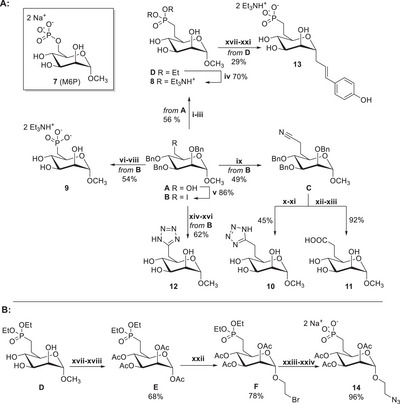
Synthesis of mannose‐6‐phosphate (MP6) analogues **8**–**14**. Reagents and conditions: i) (COCl)_2_, DMSO, CH_2_Cl_2_, −78 °C, then Et_3_N, −78 °C – r.t., 2.5 h, 80%; ii) tetraethyl methylenediphosphonate, *n*‐BuLi, THF, ‐78 °C – r.t., 3 h, 73%; iii) 10% Pd/C, H_2_, EtOH, r.t., 24 h, 97%; iv) TMSBr, 2,6‐lutidine, CH_3_CN, 20 h, 70%; v) I_2_, Ph_3_P, imidazole, 80 °C, 2 h, 86%; vi) P(OEt)_3_, 140 °C, 120 h, 75%; vii) 10% Pd/C, H_2_, EtOH, r.t., 24 h, 98%; viii) TMSBr, 2,6‐lutidine, CH_3_CN, r.t., 3 h, 74%; ix) a) ethyl cyanoacetate, 60% NaH, DMF, 70 °C, 2 h, 65%; b) LiCl, DMSO/H_2_O, 160 °C, 3 h, 76%; x) NaN_3_, Et_3_N•HCl, DMF, 90 °C, 180 h, 58%; xi) 10% Pd/C, H_2_, EtOH, r.t., 24 h, 78%; xii) NaOH, EtOH/H_2_O, 90 °C, 24 h, 96%; xiii) 10% Pd/C, H_2_, EtOH, r.t., 24 h, 91%; xiv) KCN, DMF, 70 °C, 5 h, 98%; xv) NaN_3_, Et_3_N•HCl, DMF, 90 °C, 120 h, 75%; xvi) 10% Pd/C, H_2_, EtOH, r.t., 24 h, 85%; xvii) Ac_2_O, pyridine, r.t., 24 h, 87%; xviii) Ac_2_O, H_2_SO_4_, AcOH, 40 °C, 1 h, 78%; xix) allylTMS, BF_3_•Et_2_O, TMSOTf, CH_3_CN, r.t., 16 h, 78%; xx) 4‐iodophenol, Pd(OAc)_2_, TBAB, NaHCO_3_, DMF, 85 °C,16 h, 92%; xxi) a) TMSBr, pyridine, CH_2_Cl_2_, r.t., 4 h; b) NaOMe, MeOH, r.t., 4 h, 59%; xxii) 2‐bromoethanol, BF_3_•Et_2_O, 0 °C – r.t., 16 h, 78%; xxiii) a) NaN_3_, TBAB, DMSO, r.t., 17 h, 84%; xxiv) TMSBr, pyridine, CH_3_CN, r.t., 3 h, 96%. For abbreviations, see Supporting Information.

First, we designed a fluorescent glycopeptide, compound **1**, bearing an *N*‐terminal fluorescein moiety. The aim was to develop a ligand with sufficient binding affinity for the receptor that could also be tracked via its fluorescent label, thereby enabling the use of this glycopeptide in competitive binding assays. Fluorescein was incorporated into the precursor of **1** on‐resin using 3.5 equivalents of NHS‐activated fluorescein (0.21 M in DMF). Six 6‐phosphonomethyl mannose units were introduced in solution via the CuAAC reaction onto the propargyl glycine residues of the precursor using azide **14** (Scheme 1B, 10 eq.) in a *tert*‐butanol/water mixture, catalyzed by CuSO₄·5H₂O (30 eq.) and sodium ascorbate (30 eq.). The decision to incorporate six 6‐phosphonomethyl mannose units into glycopeptide **1** was based on the assumption that multivalency enhances receptor binding, as compared to ligands containing only one or two mannose units. The 6‐phosphonomethyl modification was chosen because of its superior stability over phosphate. A similar strategy has previously been employed by Bertozzi et al.^[^ ^12a,b^ ^]^ The glycoside units were spaced by polar Lys and Ser residues to maintain the solubility of alkyne‐containing precursors during the CuAAC reaction. Notably, previous attempts using Ala spacers have resulted in completely insoluble peptides. Following the CuAAC step, the acetyl protecting groups on the mannose units were removed by treatment of the glycopeptide precursor (2 mM in anhydrous MeOH) with a saturated solution of solid sodium methoxide, yielding the final fluorescent glycopeptide **1**. The yields of the intermediate steps were considered acceptable: 23% for SPPS, 33% for CuAAC, and 60% for the deprotection of the acetyl groups.

Glycopeptides **2**–**6** were synthesized using similar synthetic procedures, with yields comparable to or exceeding that of glycopeptide **1** (see Supporting Information), most likely because of their shorter sequences and the absence of the bulky fluorescein moiety. Glycopeptide **2** was designed as a non‐fluorescent analog of compound **1** to allow the effective displacement of the fluorescent glycopeptide **1** in receptor‐binding assays, thereby enabling the determination of “non‐specific” binding contributions. Instead of the fluorescein group, Phe and Trp residues were incorporated into glycopeptide **2** to mimic the physicochemical properties of fluorescein while minimizing its potential impact on receptor interactions.

Shorter glycopeptides **3**–**6** were designed to assess whether ligands bearing only three or two 6‐phosphonomethyl mannose units can still effectively bind to the receptor. These analogues also enabled investigation of the impact of *N*‐terminal and lysine side‐chain acetylation (in compounds **4** and **6**). Acetylation was performed in solution using Ac₂O/DIPEA in ACN prior to the deprotection of the mannose acetyl groups. In the highly polar glycopeptides **3**–**6**, the incorporation of hydrophobic Trp residues aided purification by retention in RP‐HPLC and also facilitated concentration determination via UV spectroscopy.

### 2.2 Monosaccharide Mannose‐Containing Ligands

To investigate the binding affinity to CI‐M6P/IGF2R, several monosaccharide analogues of M6P were synthesized. In this study, we focused on the synthesis of methyl α‐d‐mannoside bio‐isosteres of commercially available “native” M6P **7** (Scheme 1A). The primary motivation for preparing these analogues was their chemical and, more importantly, enzymatic stability, which could provide deeper insights into the biological properties of M6P analogues in interaction with CI‐M6P/IGF2R. The synthetic strategy used for these monosaccharide derivatives was inspired by previously reported approaches,^[^ ^18^ ^]^ which were optimized in this work. Herein, we present the modular and efficient synthesis of seven M6P analogues derivatized at the C‐6 position. Ortogonally protected mannopyranoside **A** served as the key precursor^[^ ^19^ ^]^ for the synthesis of both isosteric and non‐isosteric M6P analogues. Specifically, the isosteric phosphonate **8** was obtained by homologation of mannoside **A**, involving the oxidation of the primary alcohol to the corresponding aldehyde, followed by a Wittig–Horner reaction with the ylide formed from tetraethyl methylenediphosphonate and *n*‐BuLi. Subsequent catalytic hydrogenation afforded diethyl phosphonate **C**, which was isolated from alcohol **A** with an excellent overall yield of 57%. This intermediate was then converted to the final phosphonate **8** by diethyl ester hydrolysis using TMSBr in the presence of 2,6‐lutidine. The final phosphonate **8** was isolated as a triethylammonium salt in 70% yield as the last purification step involved RP‐HPLC on a C18 column using TEAB in the mobile phase. (see Supporting Information). For the synthesis of the remaining monosaccharide derivatives **9**–**12**, the corresponding iodide **B** was used, which was prepared from mannopyranoside **A** by the Appel reaction in 86% yield. The non‐isosteric phosphonate **9** was obtained by nucleophilic substitution of iodide **B** with triethyl phosphite, followed by catalytic debenzylation and hydrolysis of the diethyl ester. The final phosphonate **9** was again isolated as a triethylammonium salt in an overall yield of 54% over three steps from iodide **B**.

Isosteric tetrazole **10** and carboxylic acid **11** were synthesized from nitrile intermediate **C** in 49% overall yield via homologation using ethyl cyanoacetate, followed by in situ ester saponification and thermal decarboxylation at 160 °C. For the preparation of tetrazole **10**, the common [3 + 2] cycloaddition of nitrile **C** with NaN₃ was used. After catalytic debenzylation, tetrazole **10** was isolated in a 45% yield (Supporting Information). A similar synthetic approach was used to obtain the shorter tetrazole **12** from iodide **B** in 62% overall yield over three steps.

Finally, isosteric carboxylic acid **11** was obtained from intermediate **C** in 92% yield via acidic hydrolysis followed by final debenzylation. Scheme 1A summarizes the synthetic strategies and overall yields of all prepared methyl d‐mannosides **8**–**12**.

As previously mentioned, to evaluate the binding affinity of the prepared methyl d‐mannosides for CI‐M6P/IGF2R, it was necessary to develop a suitable assay for quantifying this interaction. Based on our prior experience with radioligand‐binding assays, we aimed to prepare a suitable non‐hydrolyzable isosteric derivative that could be radiolabeled (e.g., with [^3^H] or [^125^I]). Since *C*‐glycosides are known to be stable analogues of natural *O*‐glycosides, we focused on the synthesis of phosphonate **13**, incorporating a 4‐hydroxyphenyl propenyl group that would enable radioisotopic labeling. Although we successfully synthesized the [^125^I]‐radiolabeled derivative of compound **13**, its relatively low binding affinity (Table 1) rendered it unsuitable for radioligand‐binding assays, as it would require the addition of an excessively high amount of radioactivity to the assay. Nevertheless, we included derivative **13** in fluorescence polarization assays (FPA), which unexpectedly displayed a relatively high affinity compared to native M6P **7** and M6Po **8**. This result, combined with the excellent chemical and enzymatic stability of the *C*‐glycoside scaffold, highlights **13** as a promising platform for further structural modification and analog development.

**Table 1:** Relative binding potencies^[^ ^a^ ^]^ (in %) of individual compounds for CI‐M6P/IGF2R:D1–D15.

Compound	IC_50_ (µM)	Potency (%)
**2**	0.48 ± 0.11	100.0
**3**	0.49 ± 0.04	96.7
**4**	0.49 ± 0.07	96.5
**5**	0.48 ± 0.10	97.1
**6**	0.66 ± 0.90	72.2
**7**	13.1 ± 2.8	3.6
**8**	11.5 ± 1.3	4.1
**9**	137 ± 2	0.35
**10**	n.b.	
**11**	56 ± 12	0.85
**12**	n.b.	
**13**	4.2 ± 0.02	11.3

*Note*: Compound potencies are reported as the mean of two independent measurements, each performed in triplicate, ± S.D.

^[a]^ The relative binding potencies were calculated as (IC_50_ of the native hormone/IC_50_ of the compound) × 100 (%). n.b. is no binding.

Phosphonate **13** was prepared from diethyl phosphonate **D** via a six‐step synthetic sequence, as detailed in the Supporting Information. In parallel, we also synthesized peracetylated phosphonate **14**, which was used as a key intermediate for preparing glycopeptides **1**–**6** applied in the FPA to evaluate all M6P analogues. Compound **14** was derived from diethyl phosphonate **D** (Scheme 1B). In the first step, the free hydroxyl groups of derivative **D** were acetylated, and after acidic acetolysis of the methyl glycoside, the intermediate phosphonate **E** was obtained in 68% yield. The stereoselective glycosylation of **E** with 2‐bromoethanol in the presence of BF_3_·Et_2_O afforded bromide **F** as the sole product in 78% yield. Subsequent azidation and hydrolysis of the diethyl ester using TMSBr in pyridine yielded phosphonate **14** in an overall yield of 26% over five steps from **D**. Importantly, the acetyl protecting groups in **14** were retained to facilitate RP‐HPLC purification of the glycopeptide precursors (see Supporting Information).

### 2.3 Dissociation Constant and Cooperativity

**Figure 3:**
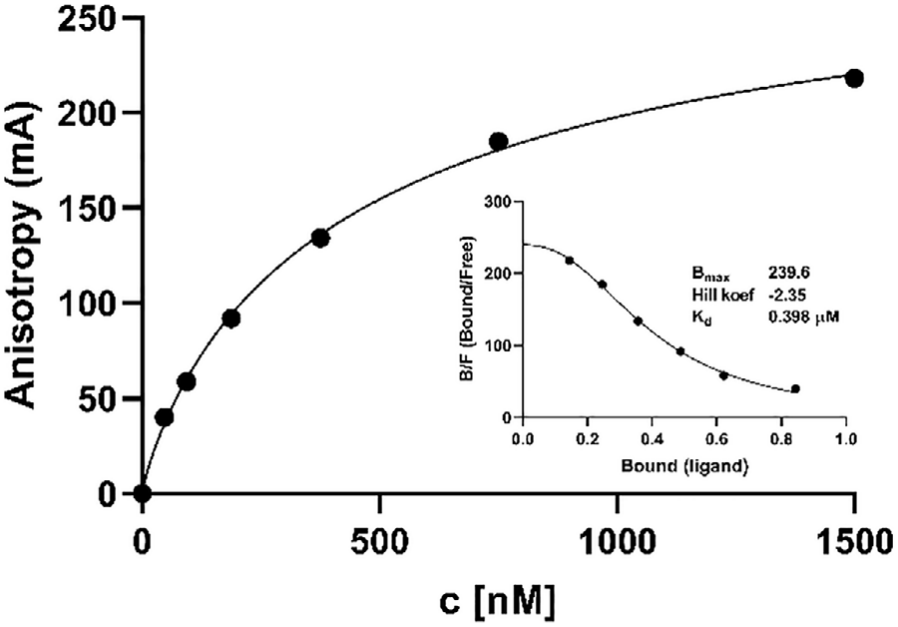
The reverse saturation plot, obtained from the binding of fluorescent glycopeptide **1** (10 nM) to soluble CI‐M6P/IGF2R:D1–D15, was determined by fluorescence polarization and Scatchard analysis (insert plot) after calculation of bound (B) and free (F) receptor concentrations. The values presented in this figure were obtained after subtracting the basal anisotropy values of peptide **1** measured in the absence of the receptor (at c = 0 [nM]). The original measured data are shown in Figure S20.

To determine the binding affinities of various synthetic or natural ligands to the M6P binding sites of CI‐M6P/IGF2R, we first developed a simple, inexpensive, and sufficiently sensitive assay for the rapid determination of binding affinity. Glycopeptide **1** was used as a fluorescent probe to measure binding to the receptor. The interaction between peptide **1** and the soluble ectodomain of CI‐M6P/IGF2R, monitored by fluorescence polarization, is illustrated in Figure 3. Using a reverse saturation binding curve, where glycopeptide **1** was titrated with varying concentrations of CI‐M6P/IGF2R, we determined the dissociation constant (K_d_) to be approximately 0.4 µM. Furthermore, this curve was used to generate a Scatchard plot from which we recalculated the maximum binding capacity (B_max_) and cooperativity coefficient (n).

The cooperativity coefficient was derived using the following equation:

$$B=B_{max}\;\frac{{\left(B/F\right)}^{n}}{K_{d}^{n}+{\left(B/F\right)}^{n}}$$

The cooperativity coefficient (n) was −2.35. Since the Hill model accounts for cooperative binding, a high value of the cooperativity coefficient suggests strong cooperativity. Based on the value of |n| (2.35), the receptor contains approximately two to three binding sites exhibiting cooperativity. This suggests that the binding of glycopeptide **1** or another ligand to the first binding site affects the binding of an additional ligand to another binding site. The presence of two to three binding sites is consistent with previous experimental findings, ^[^ ^13a^ ^]^ where domains 3 and 9 were identified as high‐affinity binding sites, whereas domain 5 displayed a lower affinity. Although some studies suggest^[^ ^20^ ^]^ that even D15 has low mannosylated‐ligand‐binding capabilities (Figure 1). Our experiment identified only 2–3 binding sites on the receptor, but it is possible that binding to any low‐affinity domains on the isolated soluble ectodomain was not detectable or that the number of binding sites varies depending on the specific ligand. However, this result provides an excellent basis for further evaluation of the binding affinities of other non‐fluorescent ligands in a competitive binding assay.

### 2.4 Competitive Fluorescence Polarization Assays

**Figure 4:**
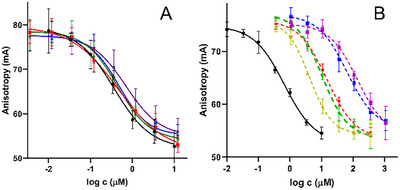
Representative titration curves of compounds for CI‐M6P/IGF2R:D1–D15. Panel A: peptide **2** (black), peptide **3** (red), peptide **4** (blue), peptide **5** (green), and peptide **6** (magenta); Panel B: peptide **2** (black), compound **7** (red), compound **8** (green), compound **9** (magenta), compound **11** (blue), and compound **13** (dark yellow).

The binding potencies of compounds **2**–**13** for the soluble D1–D15 domains of CI‐M6P/IGF2R are presented in Table 1, and representative binding curves are shown in Figure 4. The binding data indicate that glycopeptides carrying two or more 6‐phosphonomethyl mannose rings are significantly more effective in binding than compounds with a single 6‐phosphonomethyl mannose. Glycopeptides with five (**2**), three (**3**), and two 6‐phosphonomethyl mannoses (**5**) exhibited nearly identical binding affinities that were also very similar to the K_d_ value of fluorescent probe **1**. Peptides **4** and **6** are acetylated counterparts of peptides **3** and **5**, respectively, and the data show that while *N*‐terminal acetylation had no effect on the binding potency of peptides with 3–5 6‐phosphonomethyl mannose moieties, acetylation of a peptide with two mannose units can lower its binding affinity to 72% (Table 1). This suggests that acetylation plays no significant role in longer peptides, but in shorter peptides, the reduction of positive charge and elimination of free amino groups may influence receptor interaction. The results further indicate that peptide length does not play a crucial role in the receptor‐binding ability. Although glycopeptide **2** is a 15‐amino‐acid peptide with five 6‐phosphonomethyl mannose units, and glycopeptide **5** is a tripeptide with only two 6‐phosphonomethyl mannose units, both of which exhibit the same binding potency. This confirms previous findings that at least two 6‐phoshonomethyl mannose moieties are necessary for potent binding, ^[^ ^13^ ^a,^ ^21^ ^]^ as our compounds with a single 6‐phosphonomethyl mannose moiety were significantly less potent (Table 1). Natural M6P (**7**) exhibited a binding affinity of 13 µM, which is comparable to previously reported values. ^[^ ^13^ ^a,^ ^14^, ^22^ ^]^ The more stable 6‐phosphonomethyl mannose (**13**) exhibited a superior binding affinity (4.5 µM) compared to natural M6P (**7**, 13 µM). This result could be of interest for the synthesis of further M6P analogues with the potential to achieve significantly higher potency. However, the shortened chain carrying a phosphonate group in **9** was found to be unfavorable for receptor binding, leading to a decrease in the binding affinity to 137 µM. Replacing the phosphate group with tetrazole (compounds **10** and **12**) completely disrupted binding, as these compounds did not bind to the receptor. Furthermore, substitution of the phosphate group with a carboxyl group (compound **11**) resulted in a fivefold reduction in binding affinity compared to native M6P **7**. Thus, the significance of the occurrence of a phosphate or phosphonate group on the C6 carbon is evident from the decrease in affinity of compounds **10**, **11,** and **12**. However, these groups must also be in the correct location, as is evident in compound **9**. The presence of the phosphonate group also contributes to the very good affinity of compound **13,** exhibiting an almost threefold increase in binding affinity compared to that of compound **8**. However, such a good result for compound **13** is also due to the presence of a propenylphenol group at C1, as discussed in the affinity modeling section. These results indicate that the affinities of mannose ligands for CI‐M6P/IGF2R can be successfully modulated by their non‐saccharide substituents.

Overall, we can conclude that we have successfully developed a highly effective and sensitive assay for measuring the binding affinities of mannosylated ligands to CI‐M6P‐IGF2R, which is simpler and more cost‐effective than previous assays based on the detection of radioactively labeled ligands or using an ELISA sandwich (see Introduction).

### 2.5 Affinity Modeling

Using the theoretical approaches described in the Experimental Methods, we attempted to rationalize the observed differences in the affinities of compounds **7**–**13** to CI‐M6P/IGF2R. As previously mentioned, CI‐M6P/IGF2R contains four distinct binding sites (in contrast to the cation‐dependent MPR with only one^[^ ^13a^ ^]^) with distinct affinity for M6P. Domains 3 and 9 of CI‐M6P/IGF2R bind M6Ps strongly than domain 5 or even domain 15, which exhibit very weak affinity to M6P (more than 10,000 times lower affinity).^[^ ^6^, ^23^, ^24^ ^]^ To reduce the computational cost, we simulated the binding of ligands **7**–**13** to the D3, D5, and D9 domains alone. Figure S15 shows a comparison of the prepared domains. We can conclude that our models reproduce well the reported high homology of domains^[^ ^6^, ^23^, ^24^ ^]^ with their similar folding. For completeness, Figure S15 also provides an analysis of individual sequences with the identification of common motifs and their localization in the sequence. Since phosphate has pK_a_ values of ∼2.1, 7.2, and 12.0, compounds **7**–**9** and **13** were considered at pH 8 (experimental conditions) as fully deprotonated with a charge of ‐2. Compounds **10** and **12** were neutral, and compound **11** was deprotonated with a charge of −1 (estimated pK_a_ of ∼3). Prepared ligands **7**–**13** were then docked to D3, D5, and D9. For all ligands studied, we observed a similar pattern of domain binding, as reported for M6P and domains 3, 5, and 9. As an example, Figure S16 shows a comparison of the docked M6P to D9 with the experimental structure 2RL8 (bovine CI‐M6P/IGF2R:D9 with M6P).

Table S1 summarizes the estimated binding energies of ligands **7**–**13** to D3, D5, and D9 obtained using docking (GlideScore and E_model_) and the subsequent MM‐GBSA method. Since we found that the affinity of compounds **10** and **12** was unmeasurably low, we replaced it with an arbitrary value of 1000 µM for the purpose of numerical comparison of the calculated values with the experiment. The quality of the predicted binding energies can then be evaluated using the Pearson coefficient of the experimental IC_50_ and the calculated energies. Note that the predicted binding energies are negative, and the higher the IC_50_ value, the lower the affinity of the ligand. Therefore, a ligand with the highest experimental affinity should have the lowest theoretical binding energy. Outliers **10** and **12** can negatively affect the correlation. Therefore, we also report the Pearson coefficients without outliers **10** and **12**. E_model_ seems to provide the best estimates of binding energies, as these values calculated for all ligands and domains correlate best with the experiment (both with and without outliers **10** and **12**). This method was able to identify compounds **10** and **12** as the weakest ligands for all domains and simultaneously tag compound **13** as the strongest ligand for D3 and D5. Its ability to sort ligands with similar affinity is only moderate. Other approaches based on GlideScore or MM‐GBSA $\Delta G_{\mathrm{bind}}$ have either the problem of identifying the weaker binders **10** and **12** or the strongest binder **13**.

Note that the experimental IC_50_ values were obtained for the whole CI‐M6P/IGF2R, whereas we performed predictions on single domains. Therefore, we combined the results for all domains and gathered the lowest binding energy for a given ligand among all considered CI‐M6P/IGF2R domains (Table S2). For better illustration, the domain to which the ligand should preferably bind is color‐coded according to the given method. We can see that, according to E_model_, D3 and D9 are preferred over D5. This is in line with aforementioned observations for compound **7**, which has a higher affinity for D3 and D9 than for D5.^[^ ^6^, ^23^, ^24^ ^]^ The RMS deviation of E_model_ for D3 and D9 binding is ∼5.8 kcal/mol ($E_{\mathrm{model}}^{D9}-E_{\mathrm{model}}^{D3}$). However, the analogous deviation comparing the binding efficiency to D3 or D5 is ∼16 kcal/mol (similarly ∼13.5 kcal/mol for D9 and D3). Thus, the affinities of compounds **7**–**13** to D3 and D9 were much closer than those of D5. It should also be noted that docking of compound **13** into the individual domains led to a large dispersion of the complex geometries compared to compounds **7**–**12**. For example, Figure S17 shows the best docked poses of compounds **7** and **13** in D5. Any misdocking of compound **13** may overestimate its true domain affinity, resulting in a larger difference in the behavior of compound **13** with respect to the individual domains. However, the differential binding of compound **13** to D5 cannot be fully excluded.

**Figure 5:**
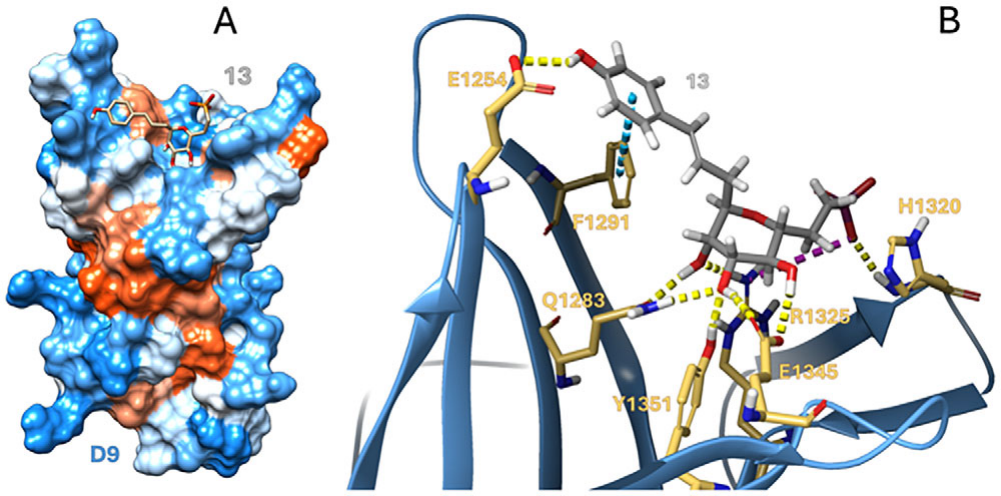
Modeled structure of ligand **13** in CI‐M6P/IGF2R:D9. **A**: Overall view; **B**: Detailed zoomed view of the binding site with the interacting CI‐M6P/IGF2R residues marked.

Since predictions of ligand affinities based on a single complex geometry can be inaccurate, especially for similarly strong ligands, we included a time dependence to our predictions via molecular dynamics. Ligands **7**–**13** in CI‐M6P/IGF2R:D9 obtained by docking were subjected to 500 ns MD simulations. Ten thousand sampled ligand‐D9 geometries were then evaluated for each ligand using MM‐GBSA, and the average ΔG values were obtained. Table S3 compares the average ΔG of ligands **7**–**13** in D9 with the experimental IC_50_ values. We observed a good correlation of predicted affinities with the experiment (both with and without outliers **10** and **12**). Let us focus more closely on the binding of ligand **13** to D9 (Figure 5), as this was the best monosaccharide ligand, outperforming M6P (**7**). Ligand **13** binds similarly to D9 as compounds **7** and **8,** and interacts with similar D9 residues. Protein interactions were monitored throughout the simulations, categorized by type, and are summarized in Figure S18. The observed stronger affinity of ligand **13** for D9 over ligand **7** is likely due to the stronger interaction of the former with F1291, T1318, K1321, and R1325. Moreover, ligand **13** exhibited additional interactions with E1254 and K1285, which were not observed for ligand **7**. In contrast, ligand **7** likely interacts more strongly with K1293 and Y1351 than compound **13**. The combined MD/MM‐GBSA approach also predicted a slightly better affinity of phosphonate **8** over phosphate **7** (Table S3), consistent with the experiment. This is likely due to the stronger interaction of phosphonate **8** with K1293, T1318, and R1325 than that observed for phosphate **7** (Figure S18A,B). The overestimation of ligand **9** affinity seen in Table S2 is reduced using the MD/MM‐GBSA approach to better correspond to the experimental trend. The significantly lower affinity of phosphonate **9**, which has a one‐carbon‐shorter exocyclic group, is due to its significantly weaker interaction with K1293, T1318, and K1321 (compared to **7**). These amino acids interact with the ‐PO_3_ group of **7** or **8** and stabilize the ligand at the binding site. For compounds **10** and **12,** these interactions are even weaker, together with other weakened interactions with H1320 and R1325. This leads to a weak affinity of ligands **10** and **12** for D9 and an early decay of the complex during the simulation. The affinity of ligand **11** is slightly overestimated, probably due to the overestimated interaction of the charge and carboxylate groups with K1293 or R1325. Interestingly, the semiempirical PM6‐D3H4 method was able to detect low‐binding ligands **10** and **12**, but was unable to correctly estimate the affinity of ligand **13** (Table S3).

As noted above, ligand **13** showed greater structural variability during docking when forming a complex with CI‐M6P/IGF2R domains. For example, docking often changed the original ^4^ *C*_1_ conformation of the pyranose ring to ^1^ *C*_4_ and flipped the ligand around the O5‐C3 axis. Such docked pose of **13** in D9, compared to the geometry discussed in the previous paragraph (which is similar to the orientation of **7** in D9) can be seen in Figure S19, together with their interactions with the D9 residues. This possible conformational benevolence in ligand **13** binding may contribute to its higher affinity for D9 by reducing the entropic penalty. On the other hand, we can presume that the change of ^4^ *C*_1_ to ^1^ *C*_4_ is energetically unfavored, and the flipped orientation of **13** is probably just a computational artifact. However, without the experimentally confirmed structure of ligand **13** in D9, an alternative ligand orientation cannot be fully ruled out.

## 3 Conclusion

In this study, we successfully designed and synthesized new stable mannose‐6‐phosphate (M6P) ligands to improve their binding affinity for the cation‐independent mannose‐6‐phosphate/IGF2 receptor (CI‐M6P/IGF2R). Using a fluorescence polarization assay, we demonstrated that multi‐mannosylated ligands exhibit significantly stronger binding than monomeric analogs, confirming the importance of, at least, bivalency in receptor interactions. Additionally, our results suggest that phosphonate derivatives could serve as more stable and potent alternatives to native M6P, and that the potency of 6‐phosphonomethyl‐mannosylated ligands can be modulated by the character of the non‐saccharide part of the molecule. Computational modeling further provided insights into ligand‐receptor interactions, highlighting key binding residues and structural factors influencing affinity. The suggested computational approach based on MD simulations exhibited high precision in estimating ligand affinity to a receptor, and thus has predictive potential. This approach may be further used to design new M6P‐containing ligands with a high affinity for CI‐M6P/IGF2R. Nevertheless, extension of the model beyond a single receptor domain is likely necessary. The combination of experimental and computational approaches enables a better understanding of receptor specificity and facilitates the rational design of next‐generation ligands. Overall, this study contributes to the development of more effective ligands for CI‐M6P/IGF2R and lays the groundwork for future research aimed at improving lysosomal targeting strategies.

## 4 Experimental Methods

### Synthesis of Compounds

The detailed protocols for the preparation of the individual target glycopeptides **1**–**6** (Figure 2), monosaccharide target compounds **8**–**13**, and key intermediate **14** (Scheme 1) are provided in the Supporting Information (Schemes S1–S6 and Figures S3‐S14).

### CI‐M6P/IGF2R:D1–D15 Production

The gene encoding human CI‐M6P/IGF2R domains 1–15 (residues AA 41–2304) fused to an N‐terminal gp67 signal peptide (MLLVNQSHQGFNKEHTSKMVSAIVLYVLLAAAAHSAFA) and C‐terminal 6‐His‐tag, PreScission protease cutting site, and TwinStrep‐tag in pFastBac‐1 vector was used for bacmid generation in DH10Bac *E. coli* (Thermo Fisher Scientific). The recombinant bacmid DNA was purified using alkaline extraction and transfected using ExpiFectamine‐Sf (Thermo Fisher Scientific) into *Spodoptera frugiperda* 9 (Sf9) cells. The first generation of recombinant baculovirus was harvested after 5 days post‐transfection and amplified in subsequent shaking cultures of Sf9 in SF‐900 II serum‐free media (Thermo Fisher Scientific) at 27 °C and 130 rpm at a density of 2 × 10^6^ cells/ml. Media containing viral particles were harvested after four days, and the amplification step was repeated to obtain high‐titer P2 stock, which was then used in large‐scale expression. At 4 days post‐infection, the cells were briefly centrifuged (500 g, 10 minutes, 4 °C) to separate them from the media, which was then adjusted to pH 8.0, and loaded onto a Strep‐Tactin‐XT (IBA Life Sciences) affinity chromatography column. The column was washed with a buffer containing 100 mM Tris‐HCl and 150 mM NaCl (pH 8.0), and the recombinant protein was eluted using the same buffer supplemented with 50 mM biotin. Expression of the recombinant protein was assessed by SDS‐PAGE and Western Blot analysis. Folding was checked by a competitive binding assay with radioactively labeled IGF2 (a natural ligand of IGF2R).^[^ ^25^ ^]^

### Fluorescence Polarization Binding Assay

We employed a fluorescence polarization (FP) binding assay to assess the binding affinity of the tested compounds for a soluble CI‐M6P/IGF2R ectodomain construct. This method monitors the rotational speed of a fluorescent probe—specifically, a fluorescein‐labeled glycopeptide **1**. Upon binding to the CI‐M6P/IGF2R protein, the probe's rotation slows, resulting in increased fluorescence polarization. This shift is quantified as anisotropy (or polarization), which reflects the ratio of bound to free probe, enabling binding affinity estimation without phase separation.^[^ ^26^ ^]^

In all experiments, the compounds were dissolved in water and serially diluted with water in Echo Qualified 384‐Well PP Source Microplates using a BRAVO pipetting station (Agilent, USA). The compounds were then pre‐spotted onto black 384‐well low‐volume round‐bottom plates (Corning, USA) using an Echo 650 liquid handler (Beckman Coulter, USA) to obtain the desired final compound concentrations. Subsequently, 5 µL of the prepared mixture (soluble receptor and glycopeptide **1** in 100 mM Tris/HCl, 150 mM NaCl, pH 8.0) was added using either a MANTIS Liquid Dispenser (Formulatrix, Dubai, United Arab Emirates) or a Clicktip pipette (Thermo Fischer Scientific, USA). The plate was sealed with Duck Tape (Hampton Research USA) to prevent evaporation, shaken for 10 s at 800 rpm, centrifuged to remove bubbles, and then incubated at room temperature. After 30 minutes, fluorescence polarization was measured using a Tecan Spark plate reader (Tecan, Switzerland) with excitation at 485 nm and emission at 535 nm.

We first analyzed the interaction between soluble CI‐M6P/IGF2R:D1–D15 and the fluorescent probe (glycopeptide **1**) using a reverse saturation binding assay in solution. The receptor concentration ranged from 0 to 1500 nM, while the fluorescein‐labeled ligand (glycopeptide **1**) was maintained at a constant concentration of 10 nM throughout the experiment. This experiment allowed us to determine an optimal receptor concentration of 300 nM for subsequent binding assays. The data from Figure S20 were analyzed using GraphPad Prism 8 (GraphPad, USA), applying the Transform–Scatchard plot and Specific binding with Hill slope models for data evaluation, after subtracting the basal anisotropy values of glycopeptide **1** measured in the absence of the receptor.

Next, competitive FP experiments were performed using a procedure analogous to previously reported methods.^[^ ^27^ ^]^ Briefly, compounds **2**–**13** in their respective dilution series were pre‐spotted onto the plate. Subsequently, 5 µL of a mixture containing CI‐M6P/IGF2R:D1–D15 (final concentration: 300 nM) and fluorescent glycopeptide **1** (final concentration: 10 nM) was added using a MANTIS Liquid Dispenser. The anisotropy data from the plate reader were plotted against the compound concentration using GraphPad Prism 8.0. A nonlinear regression analysis, “log [Inhibitor] versus response – Variable slope (four parameters),” was used to calculate the IC₅₀ values (the concentration at which half‐maximal inhibition is achieved) for each competition experiment. Relative binding affinities were calculated as (IC₅₀ of compound 2 / IC₅₀ of each compound) × 100 (%).

### Calculations

The affinity of ligands **7**–**13** was assessed using computational chemistry tools by estimating the binding energies of the ligands to models of CI‐M6P/IGF2R. Preparation of protein models and ligands, as well as docking, were carried out in the Schrödinger suite (version 2024–3).^[^ ^28^ ^]^ Molecular dynamics simulations were performed in Desmond.^[^ ^29^ ^]^ Semiempirical calculations were carried out using MOPAC.^[^ ^30^ ^]^ Based on molecular docking, we selected several candidate ligand poses in selected CI‐M6P/IGF2R domains and then evaluated these poses by their Glide Score and E_model_ (in Glide, ^[^ ^31^ ^]^ or by higher‐level methods using thermodynamic cycling. The overall workflow employed in this study is depicted in Figure S1 in the Supporting Information. First, structures of ligands **7**–**13** with ionization states were generated to capture their realistic states in solution. These ligand states were docked into the binding sites of domains 3 (D3), 5 (D5), and 9 (D9) of CI‐M6P/IGF2R (Figure S2). The individual binding sites were defined according to previous publications.^[^ ^6^, ^23^ ^]^ Up to 20 poses were generated for each ligand. The poses were scored according to their Glide Score and E_model_, and by estimating their binding energies $\Delta G_{\mathrm{bind}}$ using the molecular mechanics generalized Born surface area (MM‐GBSA) approximation in Prime. ^[^ ^32^ ^]^ The lowest energies for the individual levels of the theory and models were compared with the experimental IC_50_ values. Top docking poses (according to E_model_) of ligands **7**–**13** in D9 were then subjected to 500 ns molecular dynamics (MD) simulations (OPLS4 force field^[^ ^33^ ^]^) in a water environment. For 10,000 geometries sampled for each ligand, their binding energies were estimated using the MM‐GBSA approach at the OPLS4 level^[^ ^33^ ^]^ and averaged. The average $\Delta G_{\mathrm{bind}}$ of the studied ligands to D9 were compared with experimental IC_50_. Alternatively, the best docking poses of ligands **7**–**13** in D9 were rescored using an adapted approach described in Ref. [34]. Briefly, ligand‐D9 complexes were partially optimized at the PM6‐D3H4 level ^[^ ^35^ ^]^ using the COSMO solvation model^[^ ^36^ ^]^ (water). Only the ligand in the D9 environment was optimized. After optimization, the ligand structure was extracted from the optimized complex and optimized separately to the nearest minimum to obtain the conformational deformation of the ligand (ligand strain; ${\Delta H}_{\mathrm{strain}}^{\mathrm{lig}}$). To estimate the affinity of the ligands for D9, we used the binding enthalpy $\Delta H_{\mathrm{bind}}$ calculated as follows: $\Delta \;H_{\mathrm{bind}}=\left[H^{\mathrm{compl}}-\left(H^{\mathrm{lig}}+H^{\mathrm{prot}}\right)\right]+{\Delta H}_{\mathrm{strain}}^{\mathrm{lig}}$, where enthalpies of the individual components correspond to their geometries in the optimized complex. Energies of individual components were rescored at the PM6D3H4 level ^[^ ^35^ ^]^ using the COSMO2 solvation, as designed by Řezáč et al.^[^ ^37^ ^]^ For more details on the computational procedures, see Supporting Information. Calculated $\Delta H_{\mathrm{bind}}$ were again correlated with the experimental IC_50_ values.

## Supporting Information

The authors have cited additional references within the Supporting Information.^[^ ^38^, ^39^, ^40^, ^41^, ^42^, ^43^, ^44^, ^45^, ^46^, ^47^, ^48^, ^49^ ^]^

## Conflict of Interests

The authors declare no conflicts of interest.

## Supporting information



Supporting Information

## Data Availability

The data that support the findings of this study are available in the supplementary material of this article.
