# Supplementary material for: Design of Potent Mannose‐6‐Phosphate Derivatives as Ligands for CI‐M6P/IGF2R Using Fluorescence Polarization Assay
Source: Chemistry. 2025 May 20;31(41):e202500973. doi: 10.1002/chem.202500973 (PMC12284618; doi:10.1002/chem.202500973)
Supplement: Supplementary file 1 — Supporting Information [file CHEM-31-e202500973-s001.pdf]

Design of potent mannose-6-phosphate derivatives as ligands for CI-M6P/IGF2R using fluorescence polarization assay

Lucie Mrázková[a], Klaudia Hladoníková[a], Barbora Toncarová[b], Michal Fischer[b], Jakub Zýka[b], Jaroslav Kozák[a], Michal Kráľ[a], Milan Kožíšek[a], Jiří Jiráček[a], Jakub Kaminský[a]*, Kamil Parkan[b]*, Lenka Žáková[a]*

[a]*Institute of Organic Chemistry and Biochemistry, Czech Academy of Sciences, Flemingovo nám. 2, 166 10, Prague, Czech Republic*

[b]*Department of Chemistry of Natural Compounds, University of Chemistry and Technology, Prague, Technická 5, 166 28, Prague, Czech Republic*

[c] *Department of Cell Biology, Faculty of Science, Charles University, Viničná 7, 128 00 Prague, Czech Republic*

Table of Contents

Synthesis of mannose 6-phosphate derivatives 3
Methods 3
General Information 3
Solvents & Reagents 3
Chromatography 3
Characterization 3
Experimental Procedures and Characterization Data 4
Computational details. 18
Peptide synthesis 22
Affinity modelling 33
Copies of 1H, ^{13}C or APT and ^{31}P NMR Spectra 41
Anisotropy measurements with glycopeptide **1** and CI-M6P/IGF2R 62
References 63

Synthesis of mannose 6-phosphate derivatives

Methods

General Information

All reactions using anhydrous conditions were performed using flame-dried apparatus under an atmosphere of argon. Standard inert techniques were used in handling all air and moisture sensitive reagents.

Solvents & Reagents

Anhydrous tetrahydrofuran and dichloromethane were obtained by distillation using $LiAlH_4$ or CaH_2 as a drying agent. Other anhydrous solvents were used directly as received from commercial suppliers (Sigma-Aldrich or Acros). All other solvents were used as supplied (Analytical or HPLC grade), without prior purification. Reagents were purchased from various commercial suppliers and used as supplied, unless otherwise indicated. Distilled water was used for chemical reactions. 'Cyclohexane' refers to the fraction of cyclohexane boiling in the range 79–82 °C. Brine refers to a saturated solution of sodium chloride. Anhydrous magnesium sulfate ($MgSO_4$) was used as a drying agent after reaction workup, as indicated.

Chromatography

Column chromatography was carried out using Material Harvest silica gel (pore size 60 Å, mesh 230–400 (40–63 µm)). Thin-layer chromatography (TLC) was carried out using Merck TLC Silica gel 60 F_{254} aluminium plates. Visualization of the TLC plates was achieved using a UV lamp Spektroline-ENF-240/F (Spectronics Corporation Westbury, USA) (λ_{max} = 254 nm) and/or by spraying with cerium(IV) sulfate solution (1% in 10% H_2SO_4). Mobile phases are reported in relative composition (e.g. Hexane/EtOAc 1:1 v/v). HPLC separation was conducted on puriFlash 5.250 (Advion Interchim Scientific, France) with Luna® Omega 5 µm Polar 18C 100Å, 250 x 21.5 mm LC column. LC-MS (ESI) spectra were measured using an HP-100 liquid chromatograph with an Agilent 1290 Infinity LC system coupled to an Agilent 6460 triple quadrupole (Agilent Technologies, USA) with a Phenomenex Luna C18 column as a stationary phase and water/methanol buffered by ammonium formate as a mobile phase.

Characterization

All ^{1}H and ^{13}C NMR spectra were recorded using a Bruker Avance III 400 (401.0 MHz for ^{1}H; 162.0 MHz for ^{31}P, 100.8 MHz for ^{13}C) spectrometer. ^{1}H and ^{13}C resonances were fully assigned using H,H-COSY, H,C-HSQC and H,C-HMBC techniques. All chemical shifts are quoted on the δ scale in ppm and referenced using residual ^{1}H solvent signal in ^{1}H NMR spectra (δ(C*H*Cl_3) = 7.26 ppm, δ(*H*OD) = 4.79 ppm, δ(CD_2*H*OD) = 3.31 ppm, δ(CD_2*H*SOCD_3) = 2.50 ppm), and ^{13}C solvent signal in ^{13}C NMR spectra (δ($CDCl_3$) = 77.0 ppm, δ(CD_3OD) = 49.0 ppm, δ($(CD_3)_2SO$) = 39.7 ppm). Coupling constants (*J*) are reported in Hz with the following splitting abbreviations: s = singlet, d = doublet, t = triplet, q = quartet, sep = septet. Multiplets are reported as "m" with a range of chemical shift.

High-resolution mass spectra (HRMS) were measured on a LTQ Orbitrap XL (Thermo Fischer Scientific) spectrometer using ESI ionization technique. Nominal and exact *m*/*z* values are reported in Daltons.

Optical rotations were measured on an AUTOPOL IV (Rudolph Research Analytical, USA) polarimeter at temperature 20 °C and 589 nm sodium line with a path length *l* of 1.0 dm. Concentrations *c* are given in g/100 mL. Specific rotation values are reported as a unitless number with implied units of (deg·mL)/(g·dm).

Experimental Procedures and Characterization Data

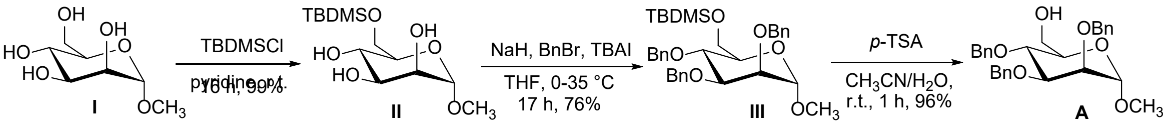

Scheme S1. Synthesis of saccharide derivative **A**.

Methyl 6-*O*-(*tert*-butyldimethylsilyl)-α-D-mannopyranoside (II)

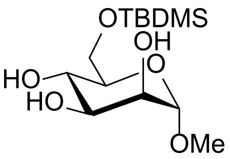

According to Ref.[38], the title compound was prepared from methyl α-D-mannopyranoside **I** (10 g; 51.5 mmol; 1.0 equiv.) following a previously described procedure. The residue was purified by flash chromatography on silica gel ($CHCl_3$/EtOH 10:1) to give product **II** (15.8 g; 99%) as a white solid. Spectral data were in agreement with previously reported.[38]

R_f = 0.25 ($CHCl_3$/EtOH 10:1)

Methyl 2,3,4-tri-*O*-benzyl-6-*O*-(*tert*-butyldimethylsilyl)-α-D-mannopyranoside (III)

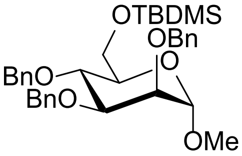

To solution of compound **II** (15.8 g; 51.2 mmol; 1.0 equiv.) in dry THF (400 mL) NaH, (60% in mineral oil; 12.3 g; 307.4 mmol; 6.0 equiv.) was added at 0 °C. The reaction mixture was stirred for 1 h. Then, tetrabutylammonium iodide (4.75 g; 12.8 mmol; 0.25 equiv.) and benzyl bromide (27.5 mL; 230.5 mmol; 4.5 equiv.) were slowly added at 0 °C. The reaction mixture was warmed up and stirred for 20 min. Then, the temperature of the reaction mixture was raised to 35 °C and the reaction was stirred at this temperature for 16 h. The progress of the reaction was monitored by TLC ($CHCl_3$/EtOH 10:1). After the complete consumption of the starting material, the reaction mixture was quenched by the addition of MeOH (5 mL). Then, the reaction mixture was concentrated to one-quarter of its original volume and diluted with CH_2Cl_2 (400 mL) and subsequently washed with a saturated solution of $NaHCO_3$ (300 mL) and brine (300 mL). The organic phase was separated and dried over anhydrous $MgSO_4$, filtered, and concentrated *in vacuo*. The residue was purified by flash chromatography on silica gel (cyclohexane/EtOAc; linear gradient: 93:7 to 10:1 with 1% Et_3N for 45 min) to give product **III** (22.5 g; 76%) as a colorless syrup. Spectral data were in agreement with previously reported.[33]

R_f = 0.55 (cyclohexane/EtOAc 5:1)

Methyl 2,3,4-tri-*O*-benzyl-α-D-mannopyranoside (A)

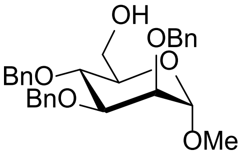

Compound **III** (12.0 g; 20.7 mmol; 1.0 equiv.) was dissolved in THF/H_2O (7:1; 128 mL). To the resulting solution, *p*-toluenesulfonic acid (~ 3.5 g) was added incrementally until the pH of the solution reached a value between 2-3. The reaction mixture was then stirred for 1 h. After the complete consumption of the starting material, CH_3CN was evaporated under reduced pressure. The resulting residue was diluted with EtOAc (300 mL), forming two distinct layers. The organic layer was separated and subsequently washed with a saturated solution of $NaHCO_3$ (200 mL) and brine (150 mL). The organic phase was separated, dried over anhydrous $MgSO_4$, filtered, and concentrated *in vacuo*. The residue was purified by flash chromatography on silica gel (cyclohexane/EtOAc; linear

gradient: 10:1 to 1:1 for 40 min) to give product **A** (9.3 g; 96%) as colorless syrup. Spectral data were in agreement with previously reported.[33]

R_f = 0.16 (cyclohexane/EtOAc 2:1)

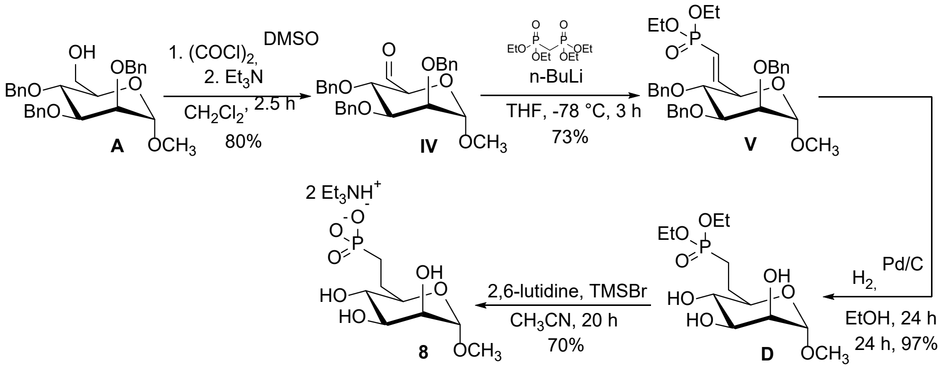

Scheme S2. Synthesis of mannose-6-phosphate analogue **8**.

Methyl 2,3,4-tri-*O*-benzyl-α-D-manno-hexodialdo-1,5-pyranoside (IV)

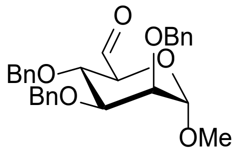

In the first flask, compound **A** (4.6 g; 9.9 mmol; 1.0 equiv.) was dissolved in dry CH_2Cl_2 (20 mL). To separate flask with dry CH_2Cl_2 (100 mL), DMSO (1.5 mL; 21.8 mmol; 2.2 equiv.) and the mixture was cooled to -78 °C. To this cooled solution, oxalyl chloride in dry CH_2Cl_2 (2M solution; 5.5 mL; 10.8 mmol, 1.1 eq) was added dropwise, and the mixture was stirred for 30 min. A solution of compound **A** (4.6 g; 9.9 mmol; 1.0 equiv.) in dry CH_2Cl_2 (20 mL) was added dropwise and stirring continued for 1 h at -78 °C. Afterwards Et_3N (6.9 mL; 50 mmol; 5 equiv.) was added and reaction mixture was stirred and slowly warmed to room temperature for 1 h. Then, the reaction was quenched with saturated NH_4Cl solution (150 mL). The separated organic layer was then washed with H_2O (80 mL), the aqueous phases were extracted with CH_2Cl_2 (200 mL). The combined organic layers were dried over $MgSO_4$, filtered, and concentrated *in vacuo*. The residue was purified by flash chromatography on silica gel (cyclohexane/EtOAc; linear gradient: 1:1 to 0:1 to 40 min) to give product **IV** (3.66 g; 80%) as white solid. Spectral data were in agreement with previously reported.[39]

R_f = 0.57 (cyclohexane/EtOAc 2:1)

Methyl 2,3,4-tri-*O*-benzyl-6-deoxy-6-(*E*)-(diethylphosphonomethylene)-α-D-mannopyranoside (V)

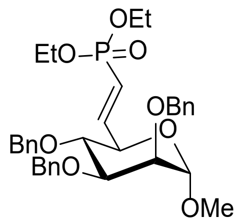

Tetraethyl methylidenebisphosphonate (2.9 mL; 11.7 mmol; 1.5 equiv.) was dissolved THF (70 mL) under argon atmosphere and the solution was cooled to -78 °C. Afterwards, *n*-BuLi (2.5M solution in hexane; 3.9 mL; 9.7 mmol; 1.25 eq) was added dropwise and the reaction mixture was stirred for 1 h at -78 °C. Then, a solution of compound **IV** (3.6 g; 7.8 mmol; 1.0 equiv.) in THF (10 mL) was added dropwise over 10 min. The stirring reaction mixture allowed to warm up to room temperature for an additional 2 h. After complete consumption of the starting material, the reaction mixture was quenched with a saturated NH_4Cl (100 mL) and the solvent was then partially evaporated to reduce the volume to 1/3 of its original volume. The mixture was diluted with EtOAc (150 mL). The separated organic layer was then washed with H_2O (80 mL), the aqueous phases were extracted with EtOAc (200 mL). The combined organic layers were dried over $MgSO_4$, filtered, and concentrated *in vacuo*. The residue was purified by flash

chromatography on silica gel (cyclohexane/EtOAc; linear gradient: 3:1 to 0:1 for 45 min) to give product **V** (3.4 g; 73 %) as white solid. Spectral data were in agreement with previously reported.[32e]

R_f = 0.43 (cyclohexane/EtOAc 1:2)

Methyl 6-deoxy-6-(diethylphosphonomethyl)-α-D-mannopyranoside (VI)

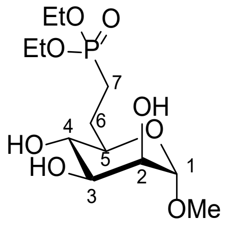

Compound **V** (3.3 g; 5.5 mmol; 1 equiv.) was dissolved in anhydrous EtOH (100 mL). Then Pd/C (10%wt; 589 mg; 0.1 equiv.) was added and H_2 was bubbled through the solution with stirring. After 24 h, the reaction mixture was filtered over Celite® and the filtrate was evaporated *in vacuo*. The residue was purified by flash chromatography on silica gel ($CHCl_3$/EtOH ; linear gradient: 9:1 to 7:1 for 40 min) to give product **D** (1.76 g; 97%) as a colorless oil. Spectral data were in agreement with previously reported. [32e]

R_f = 0.43 ($CHCl_3$/MeOH 8:1)

Methyl 6-deoxy-6-phosphonomethyl-α-D-mannopyranoside bis(triethylammonium) salt (8)

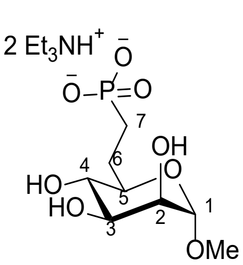

Compound **D** (322 mg; 0.98 mmol; 1.0 equiv.) was dissolved in anhydrous CH_3CN (10 mL) under argon atmosphere. Then, 2,6-lutidine (909 µL; 7.85 mmol; 8 equiv.) and Me_3SiBr (518 µL; 3.92 mmol; 4 equiv.) were added and the reaction was stirred at room temperature for 16 h. The progress of the reaction can be monitored by LC-MS. Afterwards, an additional 2,6-lutidine (2.3 mL; 19.6 mmol; 20 equiv.) and Me_3SiBr (1.3 mL; 9.8 mmol; 10 equiv.) were added and the reaction mixture was stirred for further 4 h. After the complete consumption of the starting material, the reaction was quenched with H_2O (15 mL) at 0 °C and stirring was continued at room temperature for 1 h and the organic layer was evaporated under reduced pressure. The residue was subjected to an ion exchange on Dowex® 50 WX8 resin in (Na^+) form and after 2 h, the resin was filtered off and washed several times with H_2O (50 mL). The aqueous phase was concentrated under reduced pressure. The residue was first purified by flash chromatography on silica gel (CH_3CN/H_2O 80:20) and then by HPLC on reversed phase (H_2O/MeOH + 0.5% TEAB, linear gradient: 2% to 60% over 45 min). The product was lyophilized from H_2O to give product **8** (240 mg, 70%) as a white lyophilizate.

R_f = 0.6 (75:25 CH_3CN/H_2O); $[\boldsymbol{\alpha}]_{\mathbf{D}}^{\mathbf{20}}$= +34.9 (*c* 0.3 in MeOH); ^{1}H NMR (400 MHz, D_2O, ref. 1,4-dioxane): δ 4.66 (d, $J_{1,2}$ = 1.7 Hz, 1H, H-1), 3.86 (dd, $J_{2,3}$ = 3.5, $J_{2,1}$ = 1.7 Hz, 1H, H-2), 3.66 (dd, $J_{3,4}$ = 9.6, $J_{3,2}$ = 3.5 Hz, 1H, H-3), 3.50 (t, $J_{4,3}$ = $J_{4,5}$ = 9.6 Hz, 1H, H-4), 3.45 (m, 1H, H-5), 3.34 (s, 3H, OCH_3), 3.14 (q, $J_{CH2,CH3}$ = 7.3 Hz, 12H, 2 × N(CH_2CH$_3$)$_3$), 1.99 (m, 1H, H-6b), 1.76 – 1.55 (m, 2H, H-7b, H-6a), 1.38 (m, 1H, H-7a), 1.22 (t, $J_{CH3,CH2}$ = 7.3 Hz, 18H, 2 × N(CH$_2$CH_3)$_3$); ^{13}C (101 MHz, D_2O, ref. 1,4-dioxane): δ 100.9 (C-1), 72.9 (d, $J_{C,P}$ = 16.6 Hz, C-5), 70.5 (C-3), 70.0 (C-2, C-4), 57.7 (OCH_3), 46.7 (N(CH$_2$CH$_3$)$_3$), 25.7 (d, $J_{C,P}$ = 3.21 Hz, C-6), 24.7 (d, $J_{C,P}$ = 131.8 Hz, C-7), 8.3 (N(CH$_2$CH$_3$)$_3$); ^{31}P (162 MHz, D_2O, ref. H_3PO_4): δ 23.11; **HRMS** (ESI): m/z calcd for $C_8H_{16}O_8P_4^-$: 271.0588 [M-H]$^-$; found: 271.0586.

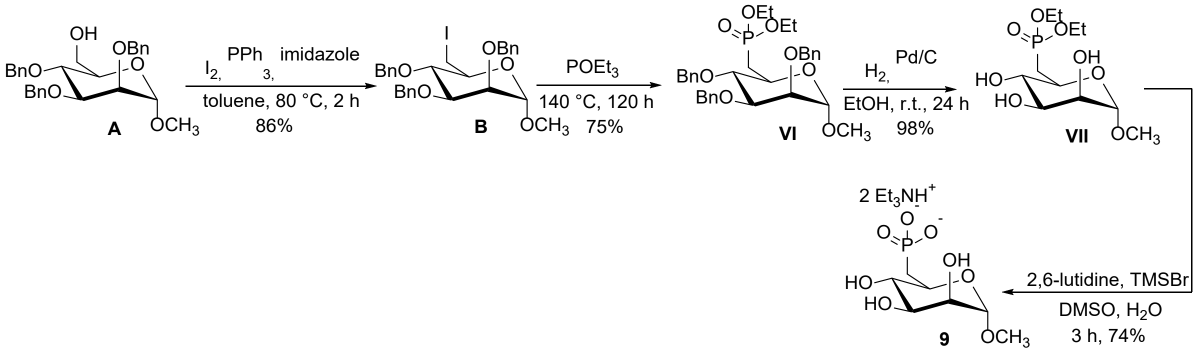

Scheme S3. Synthesis of mannose-6-phosphate analogue **9**.

Methyl 2,3,4-tri-*O*-benzyl-6-deoxy-6-iodo-α-D-mannopyranoside (B)

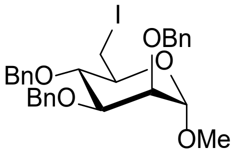

According to Ref.[39], to a stirred solution of compound **A** (2.0 g; 4.31 mmol; 1 equiv.) in toluene (45 mL) was added I_2 (1.58 g; 6.24 mmol; 1.45 equiv.), PPh_3 (3.27 g; 12.48 mmol; 2.9 equiv.), and imidazole (1.69 g; 24.75 mmol; 5.75 equiv.) were added. The reaction mixture was heated to 80 °C and stirred at this temperature for 2 h. After the complete consumption of the starting material, the reaction mixture was cooled to room temperature, and subsequently filtered, and the solid was washed with cyclohexane (100 mL). The filtrate was concentrated under reduced pressure, and the resulting crude product was purified by flash chromatography on silica gel (cyclohexane/EtOAc; linear gradient: 9:1 to 4:1 for 45 min) to give product **B** (2.1 g; 86%) as a colorless oil. Spectral data were in agreement with previously reported. [32f]

$\mathbf{R_f}$ = 0.8 (cyclohexane/EtOAc 2:1)

Methyl 2,3,4-tri-*O*-benzyl-6-deoxy-6-(diethylphosphono) -α-D-mannopyranoside (VI)

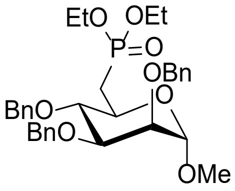

Compound **B** (1 g; 1.74; 1.0 equiv.) was dissolved in triethyl phosphite (10 mL). The reaction mixture was heated to 140 °C and stirred for 120 h. After complete consumption of the starting material, $P(OEt)_3$ was removed by lyophilization and the residue was purified by flash chromatography on silica gel (cyclohexane/ethyl acetate; linear gradient 1:1 to 0:1 for 45 min) to give product **VI** (763 mg; 75%) as a white foam.[32e]

$\mathbf{R_f}$ = 0.55 (cyclohexane/EtOAc 1:5)

Methyl 6-deoxy-6-(diethylphosphono)-α-D-mannopyranoside (VII)

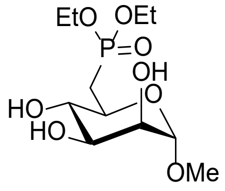

Compound **VI** (760 mg; 1.3 mmol; 1.0 equiv.) was dissolved in anhydrous EtOH (25 mL). Then Pd/C (10%wt; 277 mg; 0.2 equiv.) was added and H_2 was bubbled through the solution with stirring. After 24 h, the reaction mixture was filtered over Celite® and the filtrate was evaporated *in vacuo*. The residue was purified by flash chromatography on silica gel ($CHCl_3$/EtOH; linear gradient: 9:1 to 6:1 for 40 min) to give product **VII** (400 mg; 98%) as a colorless foam.[32e]

$\mathbf{R_f}$ = 0.1 ($CHCl_3$/MeOH 8:1)

Methyl 6-deoxy-6-phosphono-α-D-mannopyranoside bis(triethylammonium) salt (9)

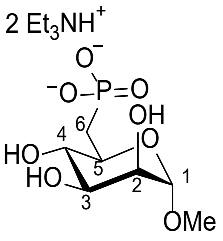

Compound **VII** (400 mg; 1.3 mmol; 1.0 equiv.) was dissolved in anhydrous CH_3CN (15 mL). under argon atmosphere. Then, 2,6-lutidine (1.77 mL; 15.3 mmol; 12 equiv.) and TMSBr (1.7 mL; 12.7 mmol; 10 equiv.) were added and the reaction was stirred at room temperature for 3 h. The progress of the reaction can be monitored by LC-MS. After the complete consumption of the starting material, the reaction was quenched with H_2O (25 mL) at 0 °C and stirring was continued at room temperature for 1 h and the organic layer was evaporated under reduced pressure. The residue was subjected to an ion exchange on Dowex® 50 WX8 resin in (Na^+) form and after 2 h, the resin was filtered off and washed several times with H_2O (60 mL). The aqueous phase was concentrated under reduced pressure. The residue was first purified by flash chromatography on silica gel (CH_3CN/H_2O 80:20) and then by HPLC on reversed phase (H_2O/MeOH + 0.5% TEAB, linear gradient: 2% to 60% over 45 min). The product was lyophilized from H_2O to give product **9** (434 mg, 74%) as a white lyophilizate.

R_f = 0.6 (75:25 CH_3CN/H_2O); $[\alpha]_D^{20}$= +29.4 (*c* 0.3 in MeOH); ^1H NMR (400 MHz, D_2O, ref. 1,4-dioxane): δ 4.64 (d, $J_{1,2}$ = 1.8 Hz, 1H, H-1), 3.85 (dd, $J_{2,3}$ = 3.5, $J_{2,1}$ = 1.7 Hz, 1H, H-2), 3.76 (dt, $J_{5,6a}$ = $J_{5,P}$ = 9.7, $J_{5,6b}$ = 3.1 Hz, 1H, H-5), 3.67 (dd, $J_{3,4}$ = 9.6, $J_{3,2}$ = 3.5 Hz, 2H, H-3), 3.40 (t, $J_{4,3}$ = $J_{4,5}$ = 9.6 Hz, H-4), 3.39 (s, 3H, OCH_3), 3.14 (q, $J_{CH2,CH3}$ = 7.4 Hz, 12H, 2 × $N(CH_2CH_3)_3$), 2.10 (ddd, $J_{6b,P}$ = 19.2, $J_{6b,6a}$ = 15.3, $J_{6b,5}$ = 3.1 Hz, 1H, H-6b), 1.77 (td, $J_{6a,6b}$ = $J_{6a,P}$ = 15.5, $J_{6a,5}$ = 9.7 Hz, 1H, H-6a), 1.21 (t, $J_{CH3,CH2}$ = 7.3 Hz, 18H, 2 × $N(CH_2CH_3)_3$); ^{13}C (101 MHz, D_2O, ref. 1,4-dioxane): δ 100.7 (C-1), 71.7 (d, $J_{C,P}$ = 11.6 Hz, C-4), 70.4 (C-3), 69.9 (C-2), 68.9 (d, $J_{C,P}$ = 3.5, C-5), 55.0 (OCH_3), 46.7 ($N(CH_2CH_3)_3$), 31.4 (d, $J_{C,P}$ = 132.9 Hz, C-6), 8.3 ($N(CH_2CH_3)_3$); ^{31}P (162 MHz, D_2O, ref. H_3PO_4): δ 21.48; **HRMS** (ESI): m/z calcd for $C_7H_{14}O_8P^-$: 257.0432 $[M-H]^-$; found: 257.0432.

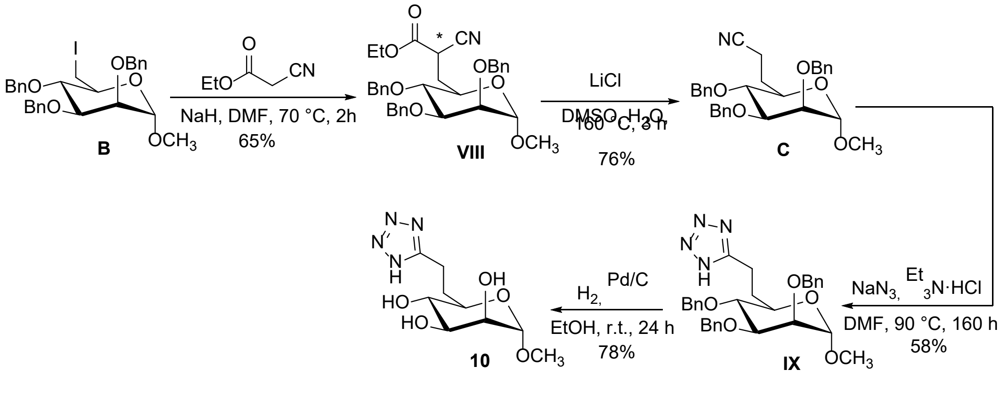

Scheme S4. Synthesis of mannose-6-phosphate analogue **10**.

Methyl 2,3,4-tri-*O*-benzyl-6-deoxy-6-(cyano(ethylcarboxy)methyl)-α-D-mannopyranoside (VIII)

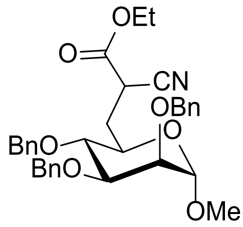

Ethyl cyanoacetate (0.7 mL; 6.61 mmol; 1.9 equiv.) was dissolved in dry DMF (5 mL) under an argon atmosphere. To this solution, NaH (60% in mineral oil; 174 mg; 4.35 mmol; 1.25 equiv.) was added, and the mixture was stirred for 30 min. Then, compound **B** (2g; 3.48 mmol; 1 equiv.), diluted in dry DMF (5 mL) was slowly added and resulting reaction mixture was warm up to 70 °C and stirred for 2 h. After complete consumption of the starting material, the reaction mixture was slowly quenched with a 1M solution of HCl until the pH reached 1. The resulting mixture was then washed with EtOAc (2 × 50 mL). The organic phases were combined, and washed with brine (70 mL), dried over $MgSO_4$, filtered, and concentrated *in vacuo*. The residue was purified by flash chromatography on silica gel (cyclohexane/EtOAc; linear gradient: 5:1 to 2:1 for

45 min) to give a mixture of two diastereomers of product **VIII** (1.26 g; 65%) in ratio 3:2, obtained as a white foam. This mixture was used in subsequent reactions without further purification.

R*f* = 0.55 (cyclohexane/EtOAc 2:1); HRMS (ESI): m/z calcd for $C_{33}H_{38}O_7N$: 560.2643 $[M+H]^+$; found: 560.2642.

Methyl 2,3,4-tri-*O*-benzyl-6,7-dideoxy-7-cyano-α-D-*manno*-heptopyranoside (C)

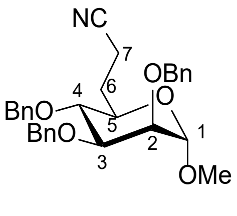

Mixture of diastereomers **VIII** (1.2 g; 2.14 mmol; 1 equiv.) was dissolved in a mixture of DMSO/H_2O (30 mL, ratio 5:1). To this solution, LiCl (136 mg; 3.22 mmol; 1.5 equiv.) was added and the reaction mixture was heated to 160 °C and stirred. After 2 hours, an additional LiCl (409 mg; 9.65 mmol; 4.5 equiv.) was added and the reaction mixture was stirred for the next 1 h. After complete consumption of the starting material, the reaction mixture was cooled to room temperature, diluted with EtOAc (150 mL), and washed with H_2O (100 mL). The organic layer was dried over anhydrous over $MgSO_4$, filtered, and concentrated *in vacuo*. The residue was purified by flash chromatography on silica gel (cyclohexane/EtOAc; linear gradient: 5:1 to 2:1 for 45 min) to give product **C** (694 mg; 75%) as a colorless hard wax.

R*f* = 0.55 (2:1 cyclohexane/EtOAc); $[\alpha]_D^{20}$= +50.7 (*c* 0.3 in $CHCl_3$); ^{1}H NMR (400 MHz, $CDCl_3$): δ 7.41 – 7.27 (m, 15H, 3 × $CH_2C_6H_5$), 4.97 (d, J_{gem} = 11.1 Hz, 1H, CH_2Ph), 4.77 (d, J_{gem} = 12.3, 1H, CH_2Ph), 4.77 (d, J_{gem} = 12.3, 1H, CH_2Ph), 4.67 (d, $J_{1,2}$ = 1.9 Hz, 1H, H-1), 4.64 – 4.59 (m, 3H, CH_2Ph), 3.88 (dd, $J_{3,4}$ = 8.8, $J_{3,2}$= 3.0 Hz, 1H, H-3), 3.80 (dd, $J_{2,3}$ = 3.0, $J_{2,1}$ = 1.9 Hz, 1H, H-2), 3.67 (t, $J_{4,3}$ = $J_{4,5}$ = 8.8 Hz, H-4), 3.63 (m, 1H, H-5), 3.33 (s, 3H, OCH_3), 2.55 – 2.38 (m, 2H, H-7a, H7b), 2.21 (m, 1H, H-6b), 1.79 (m, 1H, H-6a); ^{13}C NMR (101 MHz, $CDCl_3$) δ 138.4, 138.31, 138.25 (3 × C-Bn), 128.6 – 127.8 (9 × C*H*-Bn), 119.7 (*C*N), 99.4 (C-1), 80.4 (C-3), 77.9 (C-4), 75.3 (*C*H_2Ph), 74.5 (C-2), 73.1 (*C*H_2Ph), 72.3 (*C*H_2Ph), 69.5 (C-5), 55.2 (O*C*H_3), 27.8 (C-6), 13.5 (C-7); **HRMS** (ESI): *m/z* calcd for $C_{30}H_{33}O_5N+Na^+$: 510.2251 $[M+Na]^+$; found: 510.2247.

5-(Methyl 2,3,4-tri-*O*-benzyl-6,7-dideoxy-α-D-*manno*-heptapyranos-7-yl)tetrazole (IX)

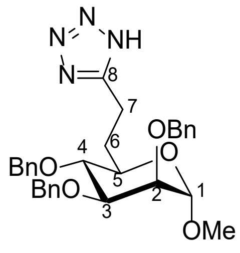

Compound **C** (400 mg; 0.82 mmol; 1.0 equiv.) was dissolved in dry DMF (10 mL) under argon. To this solution, NaN_3 (640 g; 9.84 mmol; 12 equiv.) and triethylamine hydrochloride (1.35 g; 9.84 mmol; 12 equiv.) were added. The reaction mixture was heated to 90 °C and stirred for 20 h. Then, an additional NaN_3 (640 mg; 9.84; 12 equiv.) and triethylamine hydrochloride (1.35 g; 9.84 mmol; 12 equiv.) were added and the reaction was stirred at 90 °C for another 160 h. After complete consumption of the starting material, the reaction mixture was slowly quenched with a 1M solution of HCl until the pH reached 1. The resulting mixture was then washed with EtOAc (2 × 50 mL). The organic phases were combined, and washed with brine (70 mL), dried with $MgSO_4$, filtered, and concentrated *in vacuo*. The residue was purified by flash chromatography on silica gel (cyclohexane/EtOAc; linear gradient: 1:1 to 1:5 for 45 min) to give product **IX** (253 mg; 58%) as a white solid.

R*f* = 0.55 (1:5 cyclohexane/EtOAc); $[\alpha]_D^{20}$= +37.0 (*c* 0.5 in $CHCl_3$); ^{1}H NMR (400 MHz, $CDCl_3$) δ 7.40 – 7.27 (m, 15H, 3 × $CH_2C_6H_5$), 4.97 (d, J_{gem} = 11.1 Hz, 1H, CH_2Ph), 4.88 (d, J_{gem} = 11.9 Hz, 1H, CH_2Ph), 4.72 (d, J_{gem} = 11.9 Hz, 1H, CH_2Ph), 4.65 (s, 2H, CH_2Ph), 4.65 (d, J_{gem} = 11.1 Hz, 1H, CH_2Ph), 4.61 (d, $J_{1,2}$ = 2.0 Hz, 1H, H-1), 3.93 (dd, $J_{3,4}$ = 9.0, $J_{3,2}$= 2.8 Hz, 1H, H-3), 3.86 (dd, , $J_{2,3}$ = 2.9, $J_{2,1}$ = 2.1 Hz, 1H, H-2), 3.78 (dd, $J_{4,5}$ = 9.8, $J_{4,3}$ = 9.0 Hz, 1H, H-4), 3.68 (ddd, $J_{5,4}$ = 9.8, $J_{5,6a}$ = 6.7, $J_{5,6b}$ = 3.0 Hz, 1H, H-5), 3.22 (s, 3H, OCH_3), 3.05 (ddd, J_{gem} = 16.1, $J_{7a,6a}$ = 6.9, $J_{7a,6b}$ = 5.1 Hz, 1H, H-7a), 2.95 (ddd, J_{gem} = 16.1, $J_{7b,6a}$ = 9.2, $J_{7b,6b}$ = 5.0 Hz, 1H, H-7b), 2.26 – 2.15 (m, 1H, H-6a), 2.10 – 1.98 (m, 1H, H-6b); ^{13}C NMR (101 MHz, $CDCl_3$) δ 155.9 (C-8), 138.1, 138.0, 137.5 (3 × C-Bn), 129.0 – 127.7 (9 × C*H*-Bn), 99.4 (C-1), 80.3 (C-3),

76.7 (C-4), 75.3 (CH_2Ph), 74.6 (C-2), 73.7 (CH_2Ph), 72.4 (CH_2Ph), 70.8 (C-5), 55.2 (OCH_3), 28.0 (C-6), 19.5 (C-7); **HRMS** (ESI): *m/z* calcd for $C_{30}H_{34}O_5N_4+Na^+$: 553.24214 $[M+Na]^+$; found: 553.24166.

5-(Methyl 6,7-deoxy-α-D-*manno*-heptapyranos-7-yl)tetrazole (10)

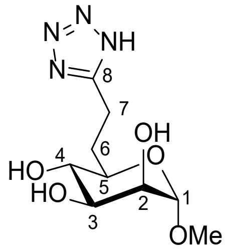

Compound **IX** (256 mg; 0.48 mmol; 1 equiv.) was dissolved in anhydrous EtOH (10 mL). Then Pd/C (10%wt; 565 mg, 1.1 equiv.) was added and H_2 was bubbled through the solution with stirring. After 24 h, the reaction mixture was filtered over Celite® and the filtrate was evaporated *in vacuo*. The residue was purified by flash chromatography on silica gel ($CHCl_3$/EtOH; linear gradient: 9:1 to 5:1 for 40 min) and HPLC on reversed phase (H_2O/MeOH; linear gradient 5% to 80% for 40 min). The product was lyophilized from H_2O to give product **10** (98 mg, 78%) as a white lyophilizate.

$\mathbf{R_f}$ = 0.15 ($CHCl_3$/MeOH 8:1); $[\alpha]_D^{20}$= +71.3 (*c* 0.4 in MeOH); ^{1}H NMR (400 MHz, D_2O, ref. 1,4-dioxane): δ 4.65 (d, $J_{1,2}$ = 1.7 Hz, 1H, H-1), 3.89 (dd, $J_{2,3}$ = 3.5, $J_{2,1}$ =1.7 Hz, 1H, H-2), 3.67 (dd, $J_{3,4}$ = 9.4, $J_{3,2}$ = 3.5 Hz, 1H, H-3), 3.55 – 3.45 (m, 2H, H-4, H-5), 3.28 (s, 3H, OCH_3), 3.20 – 3.09 (m, 2H, H-7a, H-7b), 2.38 (dddd, J_{gem} = 14.2, $J_{6a,7b}$ = 8.8, $J_{6a,7a}$ = 7.4, $J_{6a,5}$ = 2.0 Hz, 1H, H-6a), 1.98 (dddd, J_{gem} = 14.3, $J_{6b,7b}$ = 9.4, $J_{6b,7a}$ = 7.9, $J_{6b,5}$ = 6.1 Hz, 1H, H-6b); ^{13}C (101 MHz, D_2O, ref. 1,4-dioxane): δ 156.7 (C-8), 101.0 (C-1), 70.9, 70.4, 70.3 (C-3, C-4, C-5), 69.9 (C-2), 54.8 (OCH_3), 28.4 (C-6), 19.2 (C-7); **HRMS** (ESI): m/z calcd for $C_9H_{16}O_5N_4+Na^+$: 283.1013 $[M+Na]^+$; found: 283.1014.

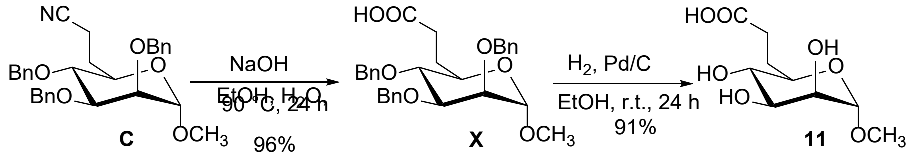

Scheme S5. Synthesis of mannose-6-phosphate analogue **11**.

Methyl-2,3,4-tri-*O*-benzyl-α-D-mannopyranosidocturonic acid (X)

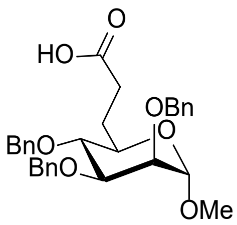

Compound **C** (250 mg; 0.51 mmol; 1 equiv.) was dissolved in EtOH (6 mL). Separately, NaOH (0.6 g; 15.4 mmol; 30 equiv.) was dissolved in H_2O (2.8 mL) of water and added to the reaction mixture. The resulting mixture was then heated to 90 °C and stirred for 24 h. After complete consumption of the starting material, the reaction mixture was first diluted with a 1M solution of HCl (10 mL). The resulting mixture was then extracted with EtOAc (2 × 50 mL). The organic phases were put together and washed with brine (70 mL), dried over $MgSO_4$, filtered, and concentrated *in vacuo*. The residue was purified by flash chromatography on silica gel (cyclohexane/EtOAc; linear gradient: 1:1 to 0:1 for 40 min) to give product **X** (249 mg; 96%) as a white solid. Spectral data were in agreement with previously reported.[40]

$\mathbf{R_f}$ = 0.88 (cyclohexane/EtOAc 1:5)

(Methyl 6,7-dideoxy-α-D-*manno*-octapyranoside)uronic acid (11)

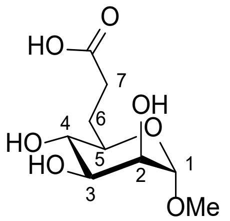

Compound **X** (236 mg; 0.47 mmol; 1.0 equiv.) was dissolved in anhydrous EtOH (10 mL). Then Pd/C (10%wt; 100 mg, 0.2 equiv.) was added and H_2 was bubbled through the solution with stirring. After 24 h, the reaction mixture was filtered over Celite® and the filtrate was evaporated *in vacuo*. The residue was purified by flash chromatography on silica gel ($CHCl_3$/EtOH; linear gradient: 9:1 to 5:1 for 40 min) and HPLC on reversed phase (H_2O/MeOH; linear gradient 2% to 80%). The product was lyophilized from H_2O to give product **11** (100 mg, 91%) as a white lyophilizate. Spectral data were in agreement with previously reported.[32d]

R*f* = 0.25 ($CHCl_3$/MeOH 8:1); $[\alpha]_D^{20}$= +78.7 (*c* 0.4 in MeOH);^{1}H NMR (400 MHz, D_2O, ref. 1,4-dioxane): δ 4.69 (d, $J_{1,2}$ = 1.8 Hz, 1H, *H*-1), 3.90 (dd, $J_{2,3}$ = 3.5, $J_{2,1}$ = 1.7 Hz, 1H, H-2), 3.69 (dd, $J_{3,4}$ = 9.2, $J_{3,2}$ = 3.4 Hz, 1H, H-3), 3.57 – 3.44 (m, 2H, H-4, H-5), 3.37 (s, 3H, OC*H*$_3$), 2.63 – 2.48 (m, 2H, H-7a, H-7b), 2.20 (dtd, J_{gem} = 15.7, $J_{6b,7b}$ = $J_{6b,7a}$ = 7.8, $J_{6b,5}$ = 2.4 Hz, 1H, H-6b), 1.75 (dddd, J_{gem} = 14.1, $J_{6a,7a}$ = 9.1, $J_{6a,7b}$ = 7.3, $J_{6a,5}$ = 6.1 Hz, 1H, H-6a); ^{13}C NMR (101 MHz, D_2O, ref. 1,4-dioxane): δ 178.3 (*C*OOH), 100.9 (C-1), 71.1, 70.5 (C-3, C-4, C-5), 70.0 (C-2), 54.8 (O*C*H$_3$), 30.1 (C-7), 26.2 (C-6); **HRMS** (ESI): m/z calcd for $C_9H_{16}O_7$+Na^+: 259.0788 [M+Na]$^+$; found: 259.0789.

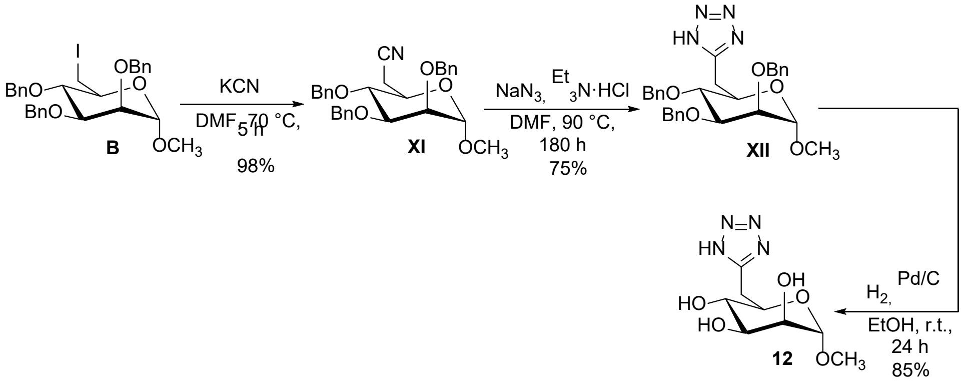

Scheme S6. Synthesis of mannose-6-phosphate analogue **12**.

Methyl 6-deoxy-6-cyano-2,3,4-tri-*O*-benzyl-α-D-mannopyranoside (XI)

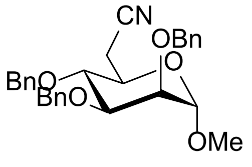

According to Ref.[32f], to a solution of compound **B** (1 g; 1.7 mmol; 1 equiv.) in dry DMF (20 mL) was added KCN (0.57 g; 8.7 mmol; 5 equiv.). The obtained reaction mixture was warmed to 70 °C and stirred for 5 h. After the complete consumption of the starting material, the reaction mixture was cooled to room temperature, and diluted with EtOAc (60 mL) and washed with a saturated solution of $NaHCO_3$ (50 mL) and brine (50 mL). The organic phase was dried over $MgSO_4$, filtered, and concentrated *in vacuo*. The residue was purified by flash chromatography (cyclohexane/EtOAc; linear gradient: 4:1 to 2:1 for 40 min) to give product **XI** (0.81 g; 98%) as colorless foam. Spectral data were in agreement with previously reported.[32f]

R*f* = 0.55 (2:1 cyclohexane/EtOAc)

5-(Methyl 2,3,4-tri-*O*-benzyl-6-deoxy-α-D-mannopyranos-6-yl)tetrazole (XII)

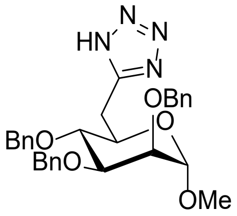

Compound **XI** (237 mg; 0.5 mmol; 1.0 equiv.) was dissolved in dry DMF (5 mL) under argon. To this solution, NaN_3 (488 mg; 7.5 mmol; 15 equiv.) and triethylamine hydrochloride (1.03 g; 7.5 mmol; 15 equiv.) were added. The reaction mixture was heated to 90 °C and stirred for 180 h. After complete consumption of the starting material, the reaction mixture was slowly quenched with a 1M solution of HCl, until the pH reached 1. The resulting mixture was then washed with EtOAc (2 × 50 mL). The organic phases were combined, and washed with brine (70 mL), dried over $MgSO_4$, filtered, and concentrated *in vacuo*. The residue was purified by flash chromatography on silica gel (cyclohexane/EtOAc; linear gradient: 1:1 to 0:1 for 40 min) to give product **XII** (194 mg; 75%) as white solid. Spectral data were in agreement with previously reported.[32f]

R_f = 0.55 (2:1 cyclohexane/EtOAc)

5-(Methyl 6-deoxy-α-D-mannopyranos-6-yl)tetrazole (12)

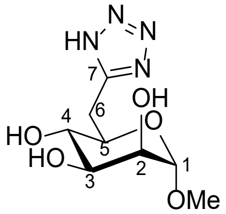

Compound **XIII** (131 mg; 0.25 mmol; 1.0 equiv.) was dissolved in anhydrous EtOH (5 mL). Then Pd/C (10 wt%; 0.27 g; 0.28; 1.1 equiv.) was added, and H_2 was bubbled through the solution with stirring. After 24 h, the reaction mixture was filtered through a pad of Celite® and the filtrate was evaporated *in vacuo*. The residue was purified by flash chromatography silica gel ($CHCl_3$/EtOH; linear gradient: 9:1 to 5:1 for 45 min) and HPLC on reversed phase (H_2O/MeOH; linear gradient: 5% to 90% for 40 min). The product was lyophilized from H_2O to give product **12** (53 mg, 85%) as a white lyophilizate. Spectral data were in agreement with previously reported.[32f]

R_f = 0.45 ($CHCl_3$/EtOH 8:1) $[\boldsymbol{\alpha}]_{\mathbf{D}}^{\mathbf{20}}$= +76.9 (*c* 0.3 in MeOH); ^{1}H NMR (400 MHz, D_2O, ref. 1,4-dioxane): δ 4.66 (d, $J_{1,2}$ = 1.7 Hz, 1H, H-1), 3.91 (dd, $J_{2,3}$ = 3.4, $J_{2,1}$ = 1.7 Hz, 1H, H-2), 3.84 (td, $J_{5,4}$ = $J_{5,6b}$ = 9.8, $J_{5,6a}$ = 2.9 Hz, 1H, H-5), 3.75 (dd, $J_{3,4}$ = 9.8, $J_{3,2}$ =3.4 Hz, 1H, H-3), 3.59 (d, $J_{4,5}$ = $J_{4,3}$ = 9.8 Hz, 1H, H-4), 3.57 (dd, $J_{6a,6b}$ =15.2, $J_{6a,5}$ = 2.9 Hz, 1H, H-6a), 3.20 (dd, J_{gem} = 15.2, $J_{6b,5}$ = 9.9 Hz, 1H, H-6b), 3.10 (s, 3Hz, OC*H*$_3$); ^{13}C NMR (101 MHz, D_2O, ref. 1,4-dioxane): δ 154.2 (C-7), 101.0 (C-1), 70.4 (C-4), 70.2 (C-3), 70.1 (C-5), 70.0 (C-2), 54.6 (OCH_3), 25.6 (C-6); **HRMS** (ESI): m/z calcd for $C_8H_{14}O_5N_4$+Na^+: 269.0856 $[M+Na]^+$; found: 269.0859.

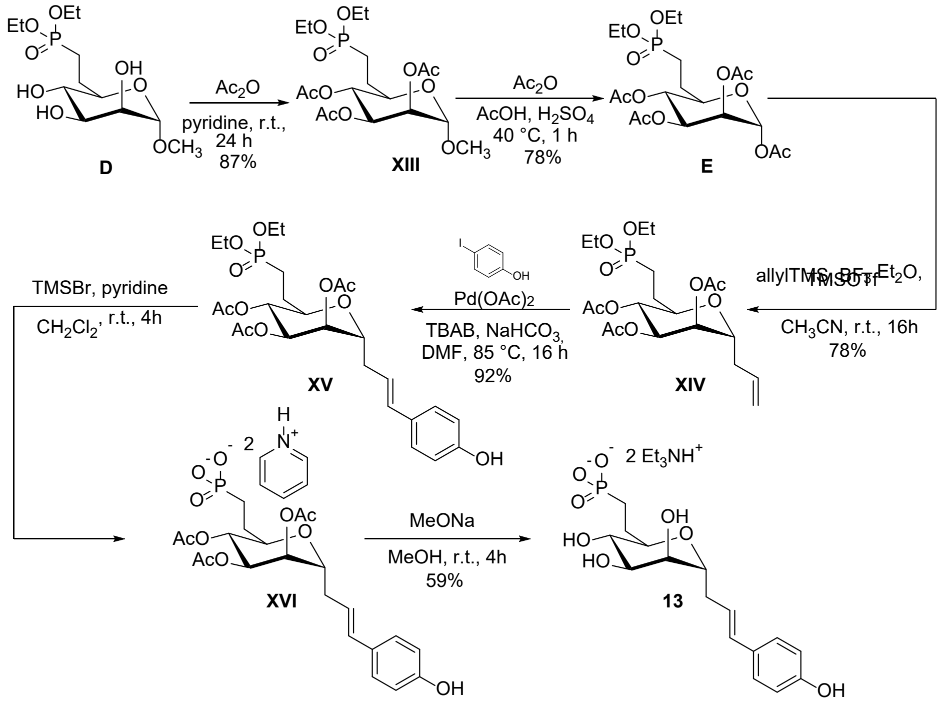

Scheme S7. Synthesis of mannose-6-phosphate analogue **13**.

Methyl 2,3,4-tri-*O*-acetyl-6-deoxy-6-(diethylphosphonomethyl)-α-D-mannopyranoside (XIII)

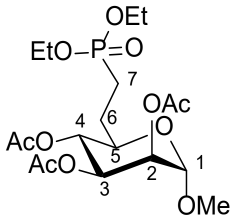

Compound **D** (2.77 g; 8.44 mmol, 1.0 equiv.) was dissolved in pyridine (50 mL; 633 mmol; 75 equiv.). To this solution, Ac_2O (40 mL; 422 mmol; 50 equiv.) was added, and the reaction mixture was stirred at room temperature for 24 h. After complete consumption of the starting material, the reaction was diluted with CH_2Cl_2 (100 mL) and washed with H_2O (100 mL), a saturated solution of $NaHCO_3$ (150 mL). The organic phase was subsequently dried over $MgSO_4$, filtered, and evaporated *in vacuo*. The residue was purified by flash chromatography on silica gel ($CHCl_3$/MeOH; linear gradient: 60:1 to 10:1 for 45 min) to give product **XIII** (3.34 g; 87%) as a colorless oil.

R*f* = 0.65 ($CHCl_3$/MeOH 8:1); $[\alpha]_D^{20}$= +51.2 (*c* 0.4 in $CHCl_3$); ^{1}H NMR (400 MHz, $CDCl_3$): δ 5.27 (dd, $J_{3,4}$ = 9.9, $J_{3,2}$ = 3.5 Hz, 1H, H-3), 5.22 (dd, $J_{2,3}$ = 3.5, $J_{2,1}$ = 1.7 Hz, 1H, H-2), 5.09 (t, $J_{4,3}$ = $J_{4,5}$ = 9.9 Hz, 1H, H-4), 4.64 (d, $J_{1,2}$ = 1.7 Hz, 1H, H-1), 4.17 – 4.04 (m, 4H, 2 × OCH_2CH$_3$), 3.73 (ddd, $J_{5,6b}$ = 12.2, $J_{5,4}$ = 9.9, $J_{5,6a}$ = 2.6 Hz, 1H, H-5), 3.37 (s, 3H, OCH_3), 2.14 (s, 3H, CH_3CO), 2.06 (m, 1H, H-6b), 2.04 (s, 3H, CH_3CO), 1.98 (s, 3H, CH_3CO), 1.88 (m, 1H, H-6a), 1.82 – 1.66 (m, 2H, *H*-7a, H-7b), 1.35 – 1.29 (2 × t, $J_{CH3,CH2}$ = 7.3 Hz, 6H, 2 × OCH$_2$CH_3); ^{13}C NMR (101 MHz, $CDCl_3$): δ 170.2, 170.11, 170.09 (3 × *C*OCH$_3$), 98.5 (C-1), 69.7 (C-2), 69.4 (C-5), 69.23 (C-4), 69.20 (C-3), 61.8, 61.7 (2 × d, $J_{C,P}$ = 3.2 Hz, O*C*H$_2$CH$_3$), 55.3 (O*C*H$_3$), 24.4 (d, $J_{C,P}$ = 4.1 Hz, C-6), 21.4 (d, $J_{C,P}$ = 143.1 Hz, C-7), 21.1, 20.94, 20.86 (3 × CO*C*H$_3$), 16.6 (d, $J_{C,P}$ = 5.8 Hz, OCH$_2$*C*H$_3$); ^{31}P (162 MHz, $CDCl_3$, ref. H_3PO_4): δ 32.02; **HRMS** (ESI): m/z calcd for $C_{18}H_{31}O_{11}P+Na^+$: 477.1496 $[M+Na]^+$; found: 477.1492.

1,2,3,4-Tetra-*O*-acetyl-6-deoxy-6-(diethylphosphonomethyl))-α-D-mannopyranose (E)

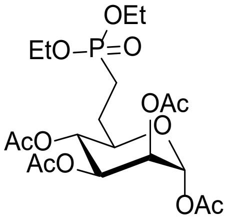

Compound **XIII** (3.0 g; 6.6 mmol; 1.0 equiv.) was dissolved in Ac_2O (8.7 mL; 92 mmol, 14 equiv.) and AcOH (70 mL; 1.2 mmol; 186 equiv.). H_2SO_4 (14 mL; 264 mmol, 40 equiv.) was then added and the reaction was stirred for 1 h at room temperature. After complete consumption of the starting material, the reaction was diluted with CH_2Cl_2 (200 mL) and this solution was poured into ice-cold H_2O (150 mL). The organic phase was separated and extracted with 2M solution of NaOH (2 × 150 mL). The organic phase was dried over $MgSO_4$, filtered, and evaporated *in vacuo*. The residue was purified by flash chromatography on silica gel ($CHCl_3$/MeOH; linear gradient: 30:1 to 10:1 for 45 min) to give product **E** (2.48 g; 78%) as a mixture of α/β anomers in a 7:1 ratio, obtained as a colorless oil. This mixture was used in subsequent reactions without further purification.

R_f = 0.45 ($CHCl_3$/MeOH 8:1)

3-(2,3,4-Tri-*O*-acetyl-6-deoxy-6-(diethylphosphonomethyl)-α-D-mannopyranosyl)prop-1-ene (XIV)

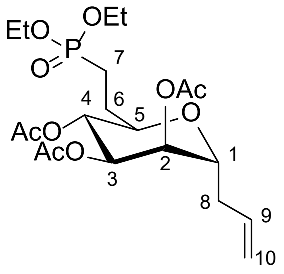

Compound **E** (2.0 g; 4.15 mmol; 1 equiv.) was dissolved in dry CH_3CN (50 mL) under an argon atmosphere. Allyltrimethylsilane (2.63 mL; 16.6 mmol; 4.0 equiv.) followed by $BF_3{\cdot}OEt_2$ (2.1 mL; 16.6 mmol; 4 equiv.) and TMSOTf (3.0 ml; 16.6 mmol; 4 equiv.) were added sequentially at room temperatureand the reaction mixture was then stirred for 16 h. After complete consumption of the starting material, the reaction was poured into ice-cold saturated aqueous solution of $NaHCO_3$ (150 mL) and extracted with CH_2Cl_2 (200 mL). The organic phase was extracted with brine (100 mL), dried over $MgSO_4$, filtered and filtered, and evaporated *in vacuo*. The residue was purified by flash chromatography on silica gel ($CHCl_3$/MeOH; linear gradient: 15:1 to 10:1 for 45 min) to give product **XIV** (1.2 g; 78%) as an inseparable mixture of α/β anomers in a 6.5:1 ratio, obtained as a colorless syrup.

R_f = 0.58 (CH_2Cl_2/MeOH 9:1); ^{1}H NMR (400 MHz, $CDCl_3$) δ 5.75 (ddt, $J_{9,10a}$ = 17.1, $J_{9,10b}$ = 10.2, $J_{9,8a}$ = $J_{9,8b}$ = 6.9 Hz, 1H, H-9), 5.24 – 5.17 (m, 2H, H-2, H-3), 5.16 (dq, $J_{10a,9}$ = 17.3, $J_{10a,10b}$ = $J_{10a,8a}$ = $J_{10a,8b}$ =1.6 Hz, 1H, H-10a), 5.13 (dq, $J_{10b,9}$ = 10.2, $J_{10b,8a}$ = $J_{10b,8b}$ =1.2 Hz, 1H, H-10b), 5.04 (t, $J_{4,3}$ = $J_{4,5}$ = 8.5 Hz, 1H, H-4), 4.13 – 4.02 (m, 4H, 2 × OCH_2CH_3), 3.95 (ddd, $J_{1,8a}$ = 9.4, $J_{1,8b}$ = 5.9, $J_{1,2}$ = 2.5 Hz, 1H, H-1), 3.62 (td, $J_{5,4}$ = $J_{5,6a}$ = 8.5, $J_{5,6b}$ = 3.5 Hz, 1H, H-5), 2.51 (dddt, $J_{8a,8b}$ = 14.8, $J_{8a,1}$ = 9.4, $J_{8a,9}$ = 7.1, $J_{8a,10a}$ = $J_{8a,10b}$ = 1.3 Hz, 1H, H-8a), 2.38 (dddt, $J_{8b,8a}$ = 14.7, $J_{8b,9}$ = 6.9, $J_{8b,1}$ = 5.8, $J_{8b,10a}$ = $J_{8b,10b}$ = 1.4 Hz, 1H, H-10b), 2.11 (s, 3H, $COCH_3$), 2.05 (s, 3H, $COCH_3$), 2.00 (s, 3H, $COCH_3$), 1.88 – 1.58 (m, 4H, H-6, H-7), 1.31 (t, $J_{CH3,CH2}$ = 7.1 Hz, 3H, OCH_2CH_3), 1.30 (t, $J_{CH3,CH2}$ = 7.0 Hz, 3H, OCH_2CH_3); ^{13}C NMR (101 MHz, $CDCl_3$) δ 170.4, 170.2, 167.0 (3 × $COCH_3$), 132.9 (C-9), 118.5 (C-10), 74.2 (C-1), 71.7 (d, $J_{C,P}$ = 17.0 Hz, C-5), 70.4 (C-2), 70.0 (C-4), 69.2 (C-3), 61.8, 61.7 (2 × d, $J_{C,P}$ = 2.8 Hz, OCH_2CH_3), 33.7 (C-8), 24.5 (d, $J_{C,P}$ = 4.1 Hz, C-6), 21.6 (d, $J_{C,P}$ = 136.2 Hz, C-7), 21.1, 21.0, 20.9 (3 × $COCH_3$), 16.6 (d, J = 5.9 Hz, OCH_2CH_3); ^{31}P NMR (162 MHz, $CDCl_3$) δ 32.50; **HRMS** (ESI): m/z calcd for $C_{20}H_{33}O_{10}P+Na^+$: 487.1704 $[M+Na]^+$; found: 487.1701.

(*E*)-4-(3-(2,3,4-Tri-*O*-acetyl-6-deoxy-6-(diethylphosphonomethyl)-α-D-mannopyranosyl)-prop-1-en-1-yl)phenol (XV)

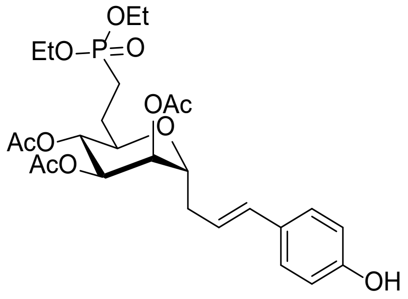

Compound **XIV** (200 mg; 431 μmol; 1.0 equiv.) was dissolved in dry DMF (8 mL) under an argon atmosphere. 4-Iodophenol (190 mg; 861 μmol; 2.0 equiv.) was added, followed addition of TBAB (139 mg; 431 μmol; 1.0 equiv), $Pd(OAc)_2$ (4.8 mg; 22 μmol, 0.05 equiv.) and $NaHCO_3$ (108 mg; 1.29 mmol; 3 eq). The reaction mixture was heated to 85 °C and stirred for 16 h. After complete consumption of the starting material, the reaction was concentrated to 1/5 volume and diluted with EtOAc and extracted $NaHCO_3$, NaCl. Organic phase was dried over $MgSO_4$, filtred andthe filtrate was evaporated under reduced pressure. The residue was purified by flash chromatography on silica gel ($CHCl_3$/MeOH; linear gradient: 20:1 to 15:1 for 45 min) to give product **XV** (221 mg; 92%) as a inseparable mixture of α/β anomers in a 6.5:1 ratio, obtained as a colorless syrup. This mixture was used in subsequent reactions without further purification.

R*f* = 0.54 (CH_2Cl_2/EtOH 15:1); **HRMS** (ESI): m/z calcd for $C_{26}H_{37}O_{11}P+Na^+$: 579.1966 $[M+Na]^+$; found: 579.1963.

(*E*)-4-(3-(6-Deoxy-6-phosphonomethyl-α-D-mannopyranosyl)-prop-1-en-1yl)phenol bis(triethylammonium) salt (13)

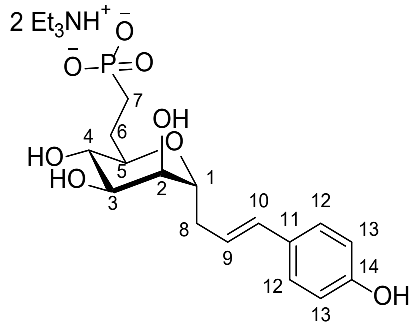

Compound **XV** (322 mg; 0.58 mmol; 1.0 equiv.) was dissolved in anhydrous CH_2Cl_2 (15 mL). under argon atmosphere. Then, pyridine (700 µL; 8.68 mmol; 15 equiv.) and Me_3SiBr (0.31 ml; 2.3 mmol; 4 equiv.) were added and the reaction was stirred at room temperature for 4 h. The progress of the reaction can be monitored by LC-MS. After the complete consumption of the starting material, the reaction was quenched with H_2O (15 mL) at 0 °C and stirring was continued at room temperature for 1 h and the organic layer was evaporated under reduced pressure. The crude product **XVII** (288 mg) was used in the subsequent reaction without further purification. The crude product (288 mg, LCMS = 498.2) was dissolved in dry MeOH (20 mL) and 1M NaOMe (1-2 mL) was added until the pH reached 10. The reaction mixture was stirred at room temperature for 4 h. After the complete consumption of the starting material, the reaction was evaporated under reduced pressure. The residue was first purified by flash chromatography on silica gel (CH_3CN/H_2O 80:20) and then by HPLC on reversed phase (H_2O/MeOH + 0.5% TEAB, linear gradient: 2% to 60% over 45 min). The product was lyophilized from H_2O to give product **13** (197 mg, 59%) as a white lyophilizate.

R*f* = 0.2 (CH_3CN/H_2O 80:20) $[\alpha]_D^{20}$= +34.2 (*c* 0.2 in H_2O); ^{1}H NMR (400 MHz, ref.*t*-BuOH): δ 7.37 (d, $J_{12,13}$ = 8.7 Hz, 2H, H-12), 6.87 (d, $J_{13,12}$ = 8.7 Hz, 2H, H-13), 6.51 (d, $J_{10,9}$ = 15.9 Hz, 1H, H-10), 6.11 (ddd, $J_{9,10}$ = 15.9, $J_{9,8b}$ = 7.8, $J_{9,8a}$ = 6.5 Hz, 1H, H-9), 4.01 (ddd, $J_{1,8b}$ = 10.1, $J_{1,8a}$ = 5.3, $J_{1,2}$ = 1,7 Hz, 1H, H-1), 3.94 (dd, $J_{2,3}$ = 3.4, $J_{2,1}$ = 1.9 Hz, 1H, H-2), 3.85 (dd, $J_{3,4}$ = 9.1, $J_{3,2}$ = 3.5 Hz, 1H, H-3), 3.48 – 3.58 (m, 2H, H-4, H-5), 3.18 (q, $J_{CH2,CH3}$ = 7.3 Hz, 12H, $(CH_3C\mathit{H}_2)_3N$), 2.68 (ddd, $J_{8b,8a}$ = 14.6, $J_{8b,1}$ = 10.1, $J_{8b,9}$ = 7.8 Hz, 1H, H-8b), 2.44 (ddd, $J_{8a,8b}$ = 14.6, $J_{8a,9}$ = 6.7, $J_{8a,1}$ = 5.5 Hz, 1H, H-8a), 1.97 – 2.10 (m, 1H, H-6a), 1.58 – 1.78 (m, 2H, H-6a, H-7b), 1.38 – 1.54 (m, 1H, H-7a), 1.27 (t, $J_{CH3,CH2}$ = 7.3 Hz, 18H, $(C\mathit{H}_3CH_2)_3N$); ^{13}C NMR (101 MHz, D_2O, ref. *t*-BuOH): δ 155.7 (C-14), 132.4 (C-10), 130.6 (C-11), 128.3 (2 × C-13), 124.6 (C-9), 116.3 (2 × C-12), 78.5 (C-1); 74.5 (d, $J_{C,P}$ = 17.0 Hz, C-5), 71.7 (C-2), 71.4 (C-3), 71.3 (C-4), 47.3 ($N(CH_2CH_3)_3$), 32.4 (C-8), 26.2 (d, $J_{C,P}$ = 3.2 Hz, C-6), 25.1 (d, $J_{C,P}$ = 133.6 Hz, C-7), 8.9 ($N(CH_2CH_3)_3$); ^{31}P

NMR (162 MHz, D_2O): δ 27.31; **HRMS** (ESI): m/z calcd for $C_{16}H_{21}O_8P^-$: 373.1058 [M-H]$^-$; found: 373.1057.

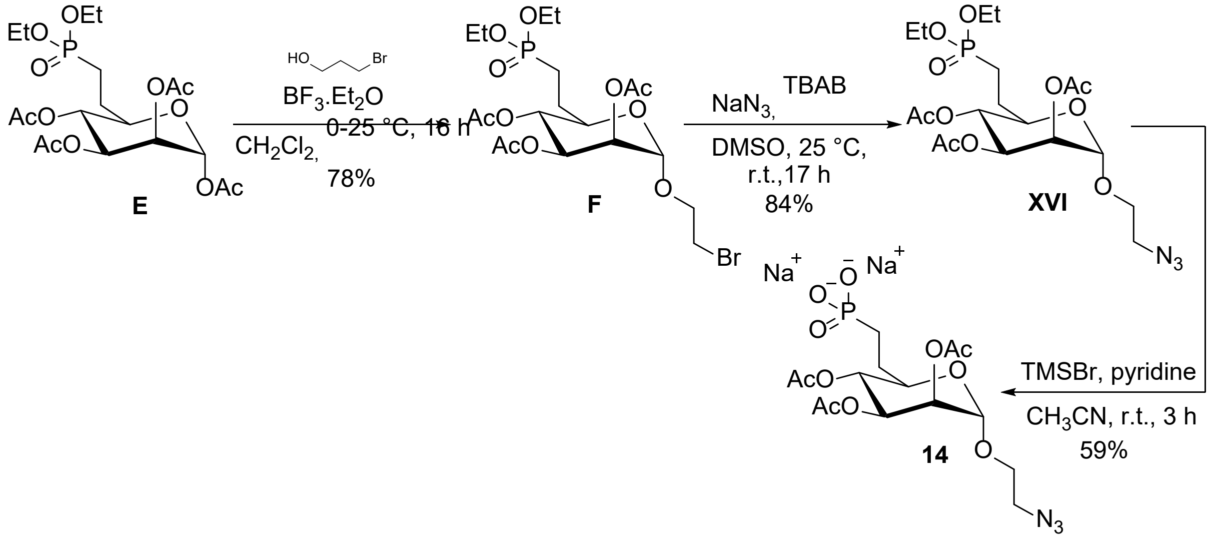

Scheme S8. Synthesis of mannose-6-phosphate analogue **14**.

2-Bromoethyl 2,3,4-tri-*O*-acetyl-6-deoxy-6-(diethylphosphonomethyl)-α-D-mannopyranoside (F)

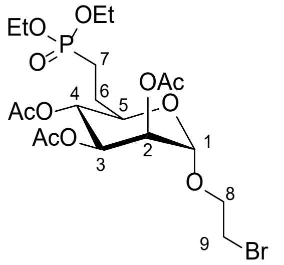

Compound **E** (1.23 g; 255 mmol; 1.0 equiv.) was dissolved in dry CH_2Cl_2 (12 mL) under an argon atmosphere. 2-Bromoethanol (362 µL; 5.10 mmol; 2.0 equiv.) followed by $BF_3{\cdot}Et_2O$ (1.57 mL; 12.75 mmol; 5.0 equiv.) was added dropwise were added sequentially at 0 °C and the reaction mixture was then stirred for 16 h at room temperature. After complete consumption of the starting material, the reaction was poured into ice-cold saturated aqueous solution of $NaHCO_3$ (150 mL) and extracted with CH_2Cl_2 (200 mL). The organic phase was extracted with brine (100 mL), dried over $MgSO_4$, filtered and filtered, and evaporated *in vacuo*. The residue was purified by flash chromatography on silica gel ($CHCl_3$/EtOH; linear gradient: 30:1 to 20:1 for 45 min) to give product **F** (1.1 g; 78%) as a pale-yellow oil.

R*f* = 0.73 ($CHCl_3$/MeOH 8:1); $[\alpha]_D^{20}$= +12.7 (*c* 0.5 in MeOH); ^{1}H NMR (400 MHz, $CDCl_3$): δ 5.30 (dd, $J_{3,4}$ = 9.9, $J_{3,2}$ = 3.5 Hz, 1H, H-3), 5.26 (dd, $J_{2,3}$ = 3.5, $J_{2,1}$ = 1.7 Hz, 1H, H-2), 5.10 (t, $J_{4,5}$ = $J_{4,3}$ = 9.9 Hz, 1H, H-4), 4.80 (d, $J_{1,2}$ = 1.7 Hz, 1H, H-1), 4.17 – 4.02 (m, 4H, 2 × OCH_2CH$_3$), 3.99 – 3.87 (m, 2H, H-5, H-8b), 3.81 (dd, $J_{8a,8b}$ = 11.1, $J_{8a,9b}$ = $J_{8a,9a}$ = 5.5 Hz, 1H, H-8a), 3.56 – 3.47 (m, 2H, H-9a, H-9b), 2.14 (s, 3H, COCH_3), 2.05 (s, 3H, COCH_3), 1.98 (s, 3H, COCH_3), 1.90 – 1.67 (m, 4H, 2 × H-7, 2 × H-6), 1.32 (t, $J_{CH3,CH2}$ = 7.2 Hz, 3H, OCH$_2$CH_3), 1.31 (t, $J_{CH3,CH2}$ = 7.2 Hz, 3H, OCH$_2$CH_3); ^{13}C NMR (101 MHz, $CDCl_3$): δ 170.2, 170.06, 170.05 (3 × *C*OCH$_3$), 97.5 (C-1), 70.0 (d, $J_{C,P}$ = 17.2 Hz, C-5), 69.7, 69.2 (C-2, C-3), 69.1 (C-4), 68.2 (C-8), 61.8, 61.7 (2 × d, $J_{CH2CH3,P}$ = 6.3 Hz, 2 × OCH$_2$CH$_3$), 29.88 (C-9), 24.5 (d, $J_{C,P}$ = 4.1 Hz, C-6), 21.3 (d, $J_{C,P}$ = 142.9 Hz, C-7), 21.04, 20.92, 20.84 (3 × COCH$_3$), 16.64, 16.58 (2 × OCH$_2$CH_3); ^{31}P NMR (162 MHz, $CDCl_3$): δ 33.97; **HRMS** (ESI): *m/z* calcd for $C_{19}H_{32}O_{11}P{+}Na^+$: 569.0758 [*M*+Na]$^+$; found: 569.0752.

2-Azidoethyl 2,3,4-tri-*O*-acetyl-6-deoxy-6-(diethylphosphonomethyl)-α-D-mannopyranoside (XVI)

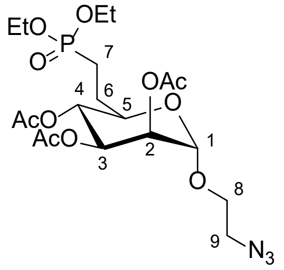

Compound **F** (1.0 g; 1.91 mmol; 1.0 equiv.) was dissolved in dry DMSO (6 mL) under an argon atmosphere. NaN_3 (741 mg; 11.4 mmol; 6 equiv.) and TBAB (1.23 g, 3.8 mmol, 2 equiv.) were added and the reaction mixture was stirred at room temperature under darkness for 17 h. After complete consumption of the starting material, the reaction was diluted with CH_2Cl_2 (150 ml) and washed with H_2O (2 × 75 ml), brine (75 ml). The organic phase was dried over $MgSO_4$, filtered, and concentrated under reduced pressure. The residue was purified by flash chromatography on silica gel ($CHCl_3$/EtOH; linear gradient: 30:1 to 20:1 for 45 min) to give product **XVI** (813 mg; 84%) as a colorless oil.

$\mathbf{R_f}$ = 0.74 ($CHCl_3$/MeOH 8:1); $[\alpha]_D^{20}$= +11.5 (*c* 0.5 in MeOH); ^{1}H NMR (400 MHz, $CDCl_3$): δ 5.30 (dd, $J_{3,4}$ = 9.9, $J_{3,2}$ = 3.5 Hz, 1H, H-3), 5.25 (dd, $J_{2,3}$ = 3.5, $J_{2,1}$ = 1.8 Hz, 1H, H-2), 5.10 (t, $J_{4,3}$ = $J_{4,5}$ = 9.9 Hz, 1H, H-4), 4.79 (d, $J_{1,2}$ = 1.8 Hz, 1H, H-1), 4.14 – 4.03 (m, 4H, 2 × O$CH_2$$CH_3$), 3.86 – 3.78 (m, 2H, H-5, H-8a), 3.60 (ddd, $J_{8b,8a}$ = 10.4, $J_{8b,9a}$ = 6.0, $J_{8b,9b}$ = 3.5 Hz, 1H, H-8b), 3.48 (ddd, $J_{9b,9a}$ = 13.3, $J_{9b,8a}$ = 6.4, $J_{9b,8b}$ = 3.5 Hz, 1H, H-9b), 3.40 (ddd, $J_{9a,9b}$ = 13.3, $J_{9a,8b}$ = 6.0, $J_{9a,8a}$ = 3.6 Hz, 1H, H-9a), 2.14 (s, 3H, COCH_3), 2.04 (s, 3H, COCH_3), 1.97 (s, 3H, COCH_3), 1.94 – 1.66 (m, 4H, 2 × H-6, 2 × H-7), 1.31 (t, *J* = 7.1 Hz, 6H, 2 × O$CH_2$$CH_3$); ^{13}C NMR (101 MHz, $CDCl_3$): δ 170.2, 170.1, 170.0 (3 × *C*OCH_3), 97.5 (C-1), 69.9 (d, $J_{5,P}$ = 17.0 Hz, C-5), 69.6 (C-2), 69.1 (C-3, C4), 66.9 (C-8), 61.8, 61.7 (2 × d, $J_{CH2CH3,P}$ = 6.3 Hz, 2 × O$CH_2$$CH_3$), 50.5 (C-9), 24.5 (d, $J_{6,P}$ = 3.9 Hz, C-6), 21.3 (d, $J_{7,P}$ = 143.5 Hz, C-7), 21.0, 20.9, 20.8 (3 × COCH_3), 16.62, 16.56 (2 × O$CH_2$$CH_3$); ^{31}P NMR (162 MHz, $CDCl_3$, ref. H_3PO_4): δ 31.89; **HRMS** (ESI): *m/z* calcd for $C_{19}H_{32}O_{11}N_3P+Na^+$: 532.1667 [*M*+Na]$^+$; found: 532.1666.

2-Azidoethyl 6-deoxy-6-phosphonomethyl-α-D-mannopyranoside disodium salt (14)

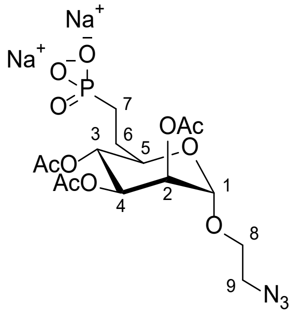

Compound **XVI** (677 mg; 1.33 mmol; 1.0 equiv.) was dissolved in dry CH_3CN (10 mL) under an argon atmosphere. Then, pyridine (0.32 mL; 3.99 mmol; 3 equiv.) and Me_3SiBr (1.72 mL; 13.29 mmol; 10 equiv.) were added and the reaction was stirred at room temperature for 3 h. The progress of the reaction can be monitored by LC-MS. After the complete consumption of the starting material, the reaction was quenched with H_2O (15 mL) at 0 °C and stirring was continued at room temperature for 1 h and the organic layer was evaporated under reduced pressure. The residue was dissolved in H_2O (20 mL) and subjected to an ion exchange on Dowex® 50 WX8 resin in (Na^+) form and after 2 h, the resin was filtered off and washed several times with H_2O (60 mL). The aqueous phase was concentrated under reduced pressure. The residue was purified by HPLC on reversed phase (H_2O/MeOH, linear gradient: 2% to 60% over 45 min). The product was lyophilized from H_2O to give product **14** (581 mg, 96%) as a white lyophilizate.

$\mathbf{R_f}$ = 0.11 (CH_3CN/H_2O 85:15); $[\alpha]_D^{20}$= +37.0 (*c* 0.4 in MeOH); ^{1}H NMR (500 MHz, DMSO-d6) δ 5.11 (dd, $J_{2,3}$ = 3.6, $J_{2,1}$ = 1.7 Hz, 1H, H-2), 5.08 (dd, $J_{3,4}$ = 10.0, $J_{3,2}$ = 3.6 Hz, 1H, H-3), 4.92 (t, $J_{4,3}$ = $J_{4,5}$ = 9.9 Hz, 1H, H-4), 4.91 (d, $J_{1,2}$ = 1.7 Hz, 1H, H-1), 3.79 (ddd, $J_{8a,8b}$ = 10.8, $J_{8a,9a}$ = 6.2, $J_{8a,9b}$ = 3.8 Hz, 1H, H-8a), 3.78 – 3.72 (m, 1H, H-5), 3.63 (ddd, $J_{8b,8a}$ = 10.8, $J_{8b,9b}$ = 6.2, $J_{8b,9a}$ = 3.5 Hz, 1H, H-8b), 3.55 – 3.42 (m, 2H, H-9), 2.09 (s, 3H, COCH_3), 2.03 (s, 3H, COCH_3), 1.92 (s, 3H, COCH_3), 1.78 – 1.1 (m, 4H, 2 × H-6, 2 × H-7);^{13}C NMR (126 MHz, DMSO-d6) δ 169.7, 169.6, 169.6 (3 × *C*OCH_3), 96.4 (C-1), 69.5 (d, $J_{5,P}$ = 16.6 Hz, C-5), 68.8, 68.70, 68.67 (C-2, C-3, C-4), 66.2 (C-8), 49.7 (C-9), 25.2 (C-6), 23.7 (d, $J_{7,P}$ = 137.7 Hz, C-7), 20.6, 20.5, 20.4 (3 × COCH_3); ^{31}P NMR (202 MHz, DMSO-d6) δ 25.4; **HRMS** (ESI): *m/z* calcd for $C_{15}H_{24}O_{11}N_3P+Na^+$: 476.1041 [*M*+Na]$^+$; found: 476.1041.

Computational details.

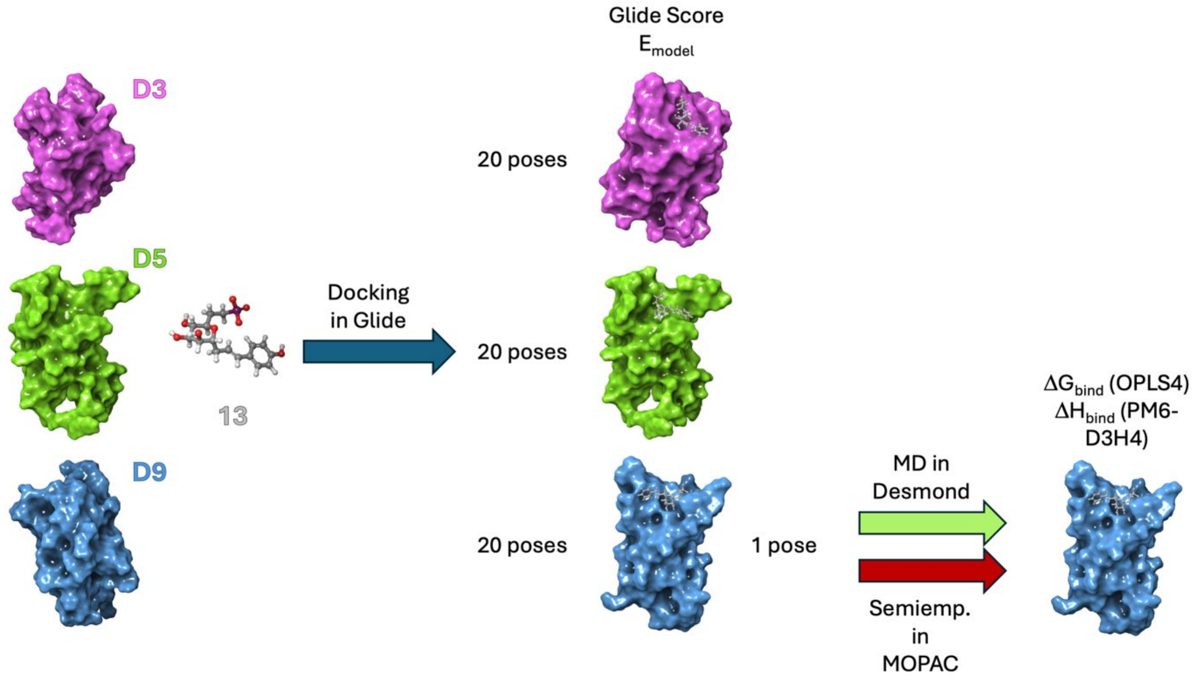

Figure S1. Computational workflow for estimation of ligand affinities towards domains D3, D5 and D9 of IGF2R.

1. Protein models

Preparation of protein models and ligands, as well as docking, were carried out in the Schrödinger suite (version 2024-3).[21] To investigate the interaction between the studied mannose derivatives and key binding domains of the IGF2 receptor, three models were prepared. The first consisted mainly of domain 3 (D3; extended by part of domain 2), the second of domain 5 and the third of domain 9 of human IGF2R. 3D structures of individual domains (**Figure S2**) were constructed from their primary sequence (UniProt ID P11717) using template-based homology modelling in the Maestro Bioluminate suite from Schrödinger. The D3 domain model was constructed from amino acids 291-433, D5 from amino acids 620-764 and D9 from amino acids 1225-1363. Note that D3 is commonly defined by residues 326-468. However, according to the structure in Ref.[41], Y324 may have some stabilizing effect on M6P, so we extended the model to Y324. A portion of domain 2 (S291-325) was further added to stabilize the structure of D3 and limit the influence of the terminal amino acids on ligand binding (see **Figure S2**). PDB structures 6P8I, 6UM2, and 6Z30 were used as templates for homology modelling of domains D3, D5, and D9, respectively. Final domain structures were prepared by Protein Preparation Workflow in Schrodinger,[21] in which ionization states were generated by Epik[42] at pH = 7.4 ± 2.0, and H-bonds were optimized by PROPKA. On such prepared protein structures, docking

grids were generated using the default grid set up in Glide.[24] Ligand interacting sites were defined as centroids of selected residues: Y324, Q348, S386, R391, E416, Y421 for D3 (Ref.[25]); Q644, W653, Y679, R687, E709, Y714 for D5 (Ref.[25]); and Y1255, Q1283, H1320, R1325, E1345, Y1351 for D9 (Ref. [6]).

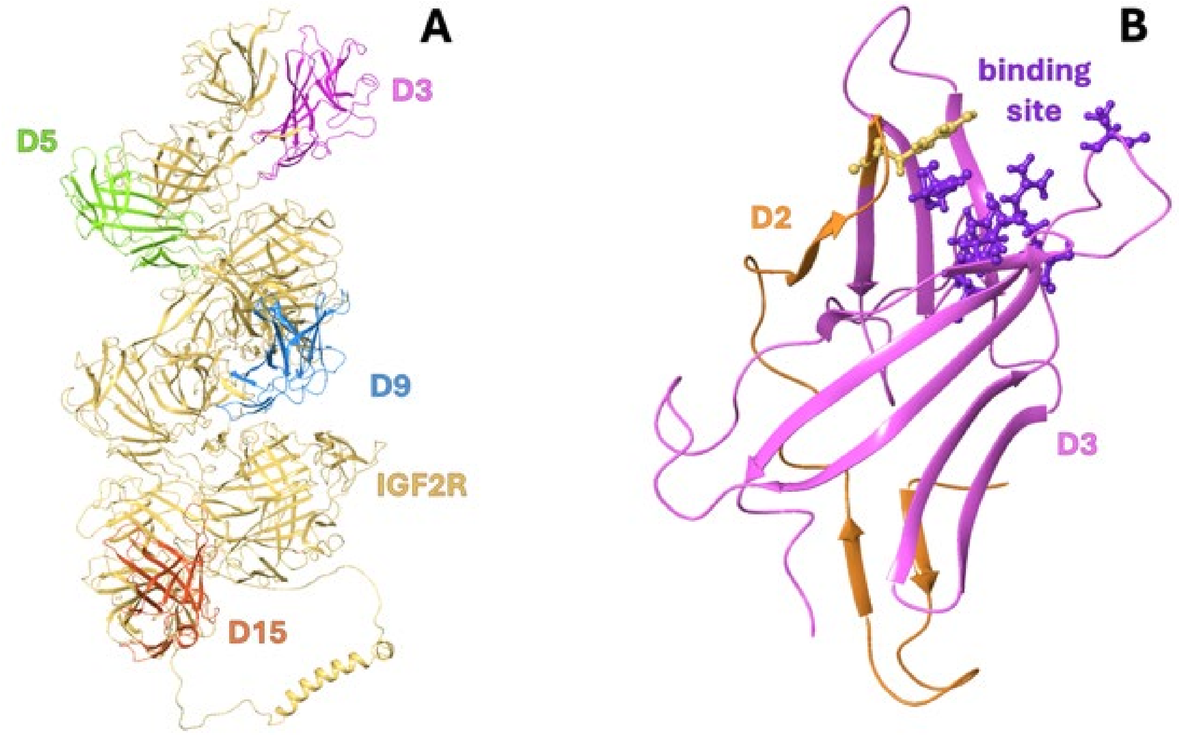

Figure S2. AlphaFold structure of IGF2R with marked domains D3, D5, and D9 (A) and the structure of D3 with a fragment of D2 and marked binding site for M6P (B).

2. Docking

Structures of ligands **7-13** for the subsequent interaction study were prepared using LigPrep[26] and the OPLS4 force field[27]. LigPrep is a tool for the robust and rapid preparation of high-quality small molecule ligand structures for further use in structure-based virtual screening. Tautomers and ionization states were generated using Epik[42] at pH = 7.4 ± 2.0 to match experimental conditions. Ligands were docked to domains by Glide[24] using the SP procedure (GlideScore version SP5.0) with flexible ligand sampling. To soften the close contacts during docking, the VdW radii of the ligand nonpolar atoms (partial charge less than 0.15) were scaled by a factor of 0.8. Partial charges for ligands were taken from OPLS4.[27] In the conformer generation step (ConfGen module), enhanced sampling (2×) was used. The top twenty docking poses for each ligand in the D3, D5 and D9 domains were scored and ranked according to their Glide SP Score and E_{model}. Additionally, all poses were also rescored by estimating their binding energies using the molecular mechanics generalized Born

surface area (MM-GBSA) approximation in Prime[26a] [43] at the OPLS4 level.[27] The solvent effect (water) was included using the VSGB 2.0 model.[44]

3. MD Simulations

Top docking poses (according to E_{model}) of ligands **7-13** in D9 were then solvated in an orthorhombic water box with a ~10 Å buffer around the protein using the System Builder of Desmond.[22a] [22b] The OPLS4 force field[27] was used to define the complex. TIP4P water molecules[45] were used to create a biologically relevant aqueous environment. To maintain electrostatic neutrality, the solvated system was charge neutralized using Na or Cl ions. When generating the MD box, the ligand was assigned the ESP charges (B3LYP/lacvp*) from the previous QM/MM calculations in QSite,[46a] [46b] while the receptor was assigned the OPLS4 charges. Solvated systems were subjected to molecular dynamics simulations in Desmond[22a] [22b] for a total of 0.5 μs within the NpT ensemble. Prior to the production simulation, the system was subjected to a multistep relaxation protocol to ensure stability.

3.1 Equilibration and Relaxation Protocol

The system was first minimized using Desmond's standard N_PT relaxation protocol. This consists of a 100 ps Brownian dynamics simulation under the NVT ensemble at 10 K, where solute heavy atoms were restrained (a harmonic force constant of 50 kcal/mol/Å^2). Then, a 12 ps equilibration under the NVT ensemble (T = 10 K) follows with the same restraints on the solute heavy atoms. A subsequent 12 ps NpT equilibration step was conducted while maintaining a temperature of 10 K and p of 1 atm. Subsequently, the temperature was gradually raised to 300 K over a final 12 ps NpT equilibration step. The relaxation protocol finalizes with a 24 ps NpT simulation at 300 K, where all restraints on the solute heavy atoms were removed.

3.2 Production Run

Equilibrated systems were subjected to 500 ns MD simulations maintaining a constant pressure and temperature, using the Martyna-Tobias-Klein method.[47] The temperature was controlled at 300 K with a thermostat coupling constant of 1.0 ps (Nosé–Hoover thermostat), while the pressure was maintained at 1.01325 atm with a barostat coupling constant of 2.0 ps (Martyna–Tobias–Klein barostat). The time step used for the integration of Newton's equations of motion was 2 fs for bonded interactions and short-range nonbonded interactions, whereas long-range nonbonded interactions were updated every 6 fs using the particle mesh Ewald method.[48] Throughout the simulation, the system was saved every 50 ps, producing 10,000 frames for subsequent analysis.

4. Post-Simulation Analysis

Post-simulation analysis was performed using various tools within the Maestro 13.8 software to investigate the structural dynamics and energetics of the ligand-D9 interaction. Root-mean-square deviation (RMSD) analysis was conducted to assess the stability of the protein complex over time, and the trajectory was processed using a 50 ps interval for accurate and representative results. Additionally, the binding free energies of a ligand to D9 were estimated for sampled MD geometries using the molecular mechanics generalized Born surface area (MM-GBSA) approximation using Prime[26a] [43] at the OPLS4 level.[27] The solvent effect (water) was included using the VSGB 2.0 model.[44]

5. Semiempirical Calculations

Alternatively, the best docking poses of ligands **7-13** to D9 were rescored using an adapted approach described in Ref.[28] Briefly, ligand-D9 complexes were partially optimized at the PM6-D3H4 level[29] using the COSMO solvation model[30] (water) in MOPAC.[23] Only the ligand in the D9 environment was optimized. The localized molecular orbitals algorithm (MOZYME) was used during the SCF cycle. The molecular mechanics correction (MMOK) was used, and the dielectric constant for COSMO was set to 78.4 (EPS = 78.4). After optimization, the ligand structure was extracted from the optimized complex and optimized separately to the nearest minimum to obtain the conformational deformation of the ligand (ligand strain; $\Delta\mathrm{H}_{\mathrm{strain}}^{\mathrm{lig}}$). To estimate the affinity of the ligands for D9, we used the binding enthalpy $\Delta\mathrm{H}_{\mathrm{bind}}$ calculated as follows: $\Delta\mathrm{H}_{\mathrm{bind}} = \left[\mathrm{H}^{\mathrm{compl}} - (\mathrm{H}^{\mathrm{lig}} + \mathrm{H}^{\mathrm{prot}})\right] + \Delta\mathrm{H}_{\mathrm{strain}}^{\mathrm{lig}}$, where enthalpies of the individual components correspond to their geometries in the optimized complex. Energies of individual components were rescored at the PM6D3H4 level[29] using the COSMO2 solvation, as designed by Řezáč et al.[31]

Peptide synthesis

Peptide P1 ($C_{75}H_{92}N_{16}O_{21}$, Exact Mass: 1552.6623, Molecular Weight: 1553.6520)

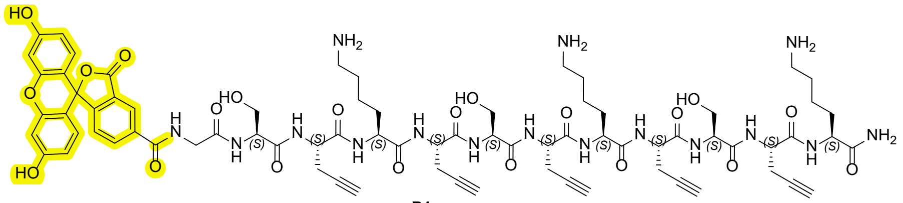

P1

The compound **P1** was synthesized on Fmoc-Rink MBHA Amide resin (LL, 0.42 mmol/g) using 60 µmol per synthetic batch. All Fmoc amino acids, including the non-standard Fmoc-L-propargylglycine, were purchased from Iris Biotech GmbH.

For each position of the peptide, two coupling steps of 75 minutes each were performed. The first coupling was carried out with 0.7 ml of 0.4 M amino acid in DMF with 0.45 M HOBt, 0.66 ml of 0.4 M HBTU in DMF, and 0.53 ml of 1 M DIPEA in DMF. The second coupling was performed with 1 ml of 0.4 M amino acid in DMF with 0.45 M HOBt, and 0.44 ml of 1 M DIC in DMF. The resin was deprotected using 2 ml of 20% piperidine in DMF, with two treatments of 2 minutes and 20 minutes, respectively.

After the deprotection of the N-terminal glycine residue, the resin was washed with a mixture of DMF and DCM (7:3). Then, 100 mg of NHS-activated fluorescein (211 µmol, Thermo Scientific) was added in a mixture of 0.7 ml of DMF and 0.3 ml of DCM. The resin was stirred for 8 hours, then washed with DCM, dried under vacuum, and treated for 2 hours with a cleavage cocktail consisting of 5 ml of TFA, 0.2 ml of water, 0.2 ml of TIS, and 0.2 ml of DODT.

The crude peptide was precipitated with 45 ml of cold diethyl ether and purified on a semipreparative C4 column (YMC-Triart Bio C4, 250 × 10 mm) at a flow rate of 3 ml/min using a linear gradient of 8% to 80% acetonitrile in water with 0.1% TFA over 30 minutes. The product **P1** was lyophilized, and its identity confirmed by ESI mass spectrometry in a positive mode (found 1552.66, calculated 1552.66, deconvoluted spectrum). The yield was 21 mg (23%).

<u>Glycopeptide 1A</u> ($C_{150}H_{212}N_{31}O_{76}P_5$ Exact Mass: 3818.2365, Molecular Weight: 3820.3558)

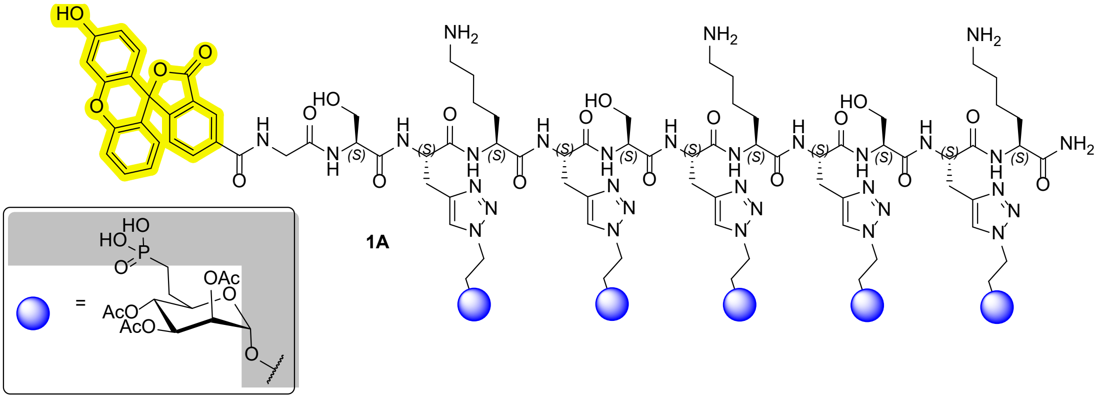

1A

Peptide **P1** (6 mg, 3.86 µmol) and compound **14** (shown below, 18 mg, 40 µmol) were dissolved in 3.5 mL of a *tert*-BuOH/water mixture (1:1, sonicated and purged with argon). Subsequently, $CuSO_4 \cdot 5H_2O$ (30 mg, 120 µmol) and sodium ascorbate (24 mg, 120 µmol) were added in 2 mL of water (sonicated and purged with argon). After 20 minutes of stirring in the dark, the reaction mixture was diluted with water, desalted using a C18 Chromabond cartridge (6 mL), and eluted with acetonitrile. The crude product was purified using the same method as for peptide **P1**. The product **1A** was lyophilized, and its identity was confirmed by ESI mass spectrometry in a negative mode (found: 3817.225; calculated: 3818.229, deconvoluted spectrum). The yield was 4.8 mg (33%).

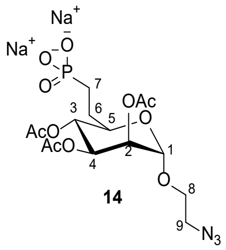

14

Compound 1 ($C_{120}H_{182}N_{31}O_{61}P_5$, Exact Mass: 3188.0780, Molecular Weight: 3189.8008)

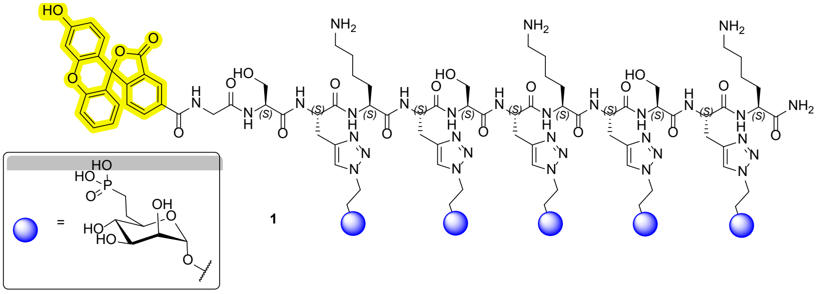

Glycopeptide 1A (3.8 mg, 1 μmol) was dissolved in 0.5 mL of dry MeOH. Then, solid CH_3ONa was added to form a saturated, highly basic solution (confirmed by pH indicator paper). The completeness of the reaction was checked by LC-MS. The product was purified on a semipreparative C4 column (YMC-Triart Bio C4, 250 × 10 mm) at a flow rate of 3 mL/min using a linear gradient of 0.8% to 80% acetonitrile in water with 0.1% TFA over 30 minutes. The product was lyophilized, and its identity was confirmed by high-resolution ESI mass spectrometry in a negative mode (**Figure S3**) (found: 3188.0796; calculated: 3188.0780, deconvoluted spectrum) and the purity (≥95%) checked by analytical HPLC (**Figure S4**). The yield was 1.9 mg (60%).

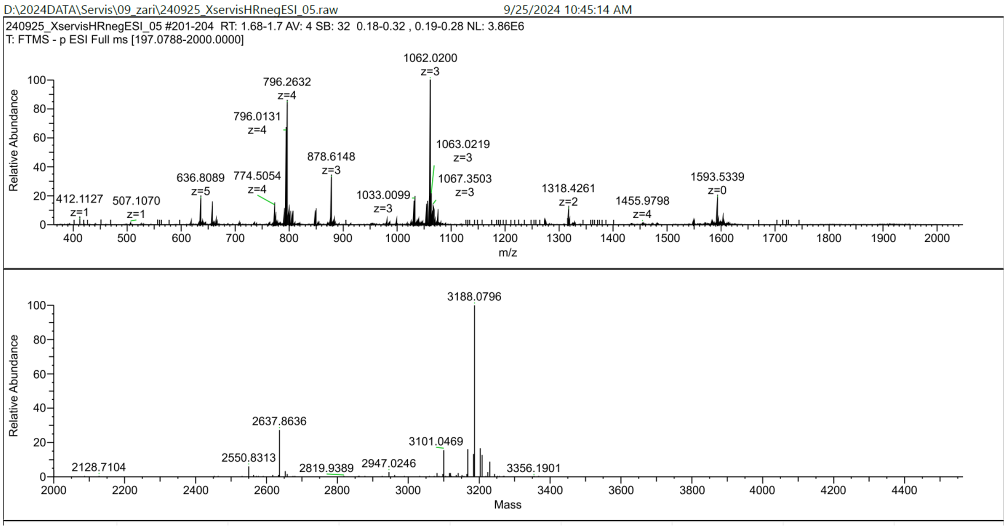

Figure S3. Deconvoluted high-resolution ESI mass spectrum of compound **1** in a negative mode.

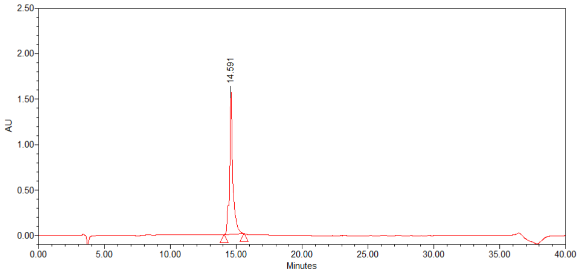

Figure S4. Analytical HPLC chromatogram of compound **1** on C4 column (YMC-Triart Bio C4, 250 × 4 mm) at a flow rate of 1 mL/min using a linear gradient of 0.8% to 80% acetonitrile in water with 0.1% TFA over 30 minutes. Detection at 218 nm.

Peptide P2 ($C_{76}H_{103}N_{19}O_{18}$, Exact Mass: 1569.7728, Molecular Weight: 1570.7750)

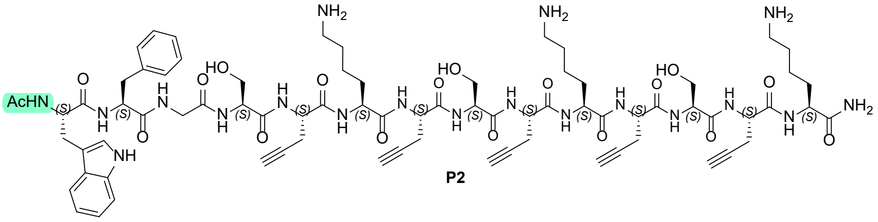

The peptide was prepared using the same methodology as **P1** using a 50 µmol synthetic batch. However, instead of coupling with fluorescein, the peptide was N-terminally acetylated with 5% DIPEA and 10% Ac_2O in DMF for 2 × 10 minutes. The peptide was purified as described for **peptide P1**. The product was lyophilized, and its identity was confirmed by liquid chromatography coupled with mass spectrometry (LC-MS) in positive mode (found: 786.4 for M^{2+} and 524.7 for M^{3+}; calculated: 786.4 for M^{2+} and 524.6 for M^{3+}). The yield was 25 mg (32%).

Glycopeptide 2A ($C_{151}H_{223}N_{34}O_{73}P_5$, Exact Mass: 3835.3471, Molecular Weight: 3837.4788)

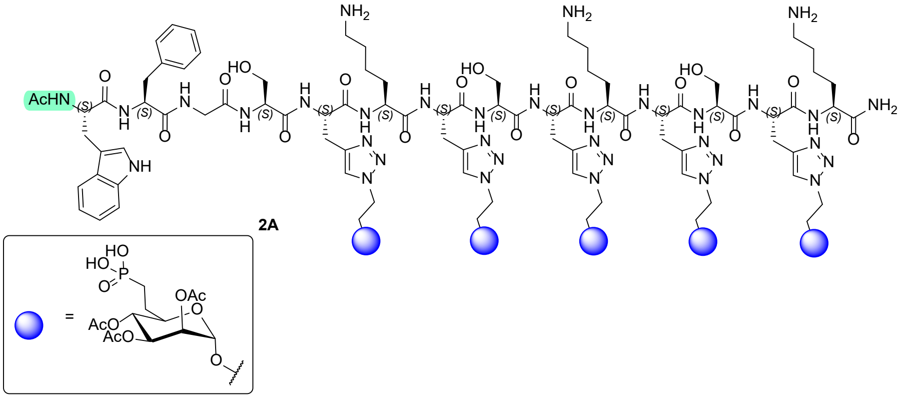

The peptide **P2** was "clicked" with **compound 14** by the same methodology as for **1A**. The product **2A** was lyophilized, and its identity was confirmed by high-resolution mass ESI spectrometry in a negative mode (found: 3835.3458; calculated: 3835.3471, deconvoluted spectrum). The yield was 5.5 mg (45%).

Compound 2 ($C_{121}H_{193}N_{34}O_{58}P_5$, Exact mass: 3205.1886, Molecular Weight: 3206.9238)

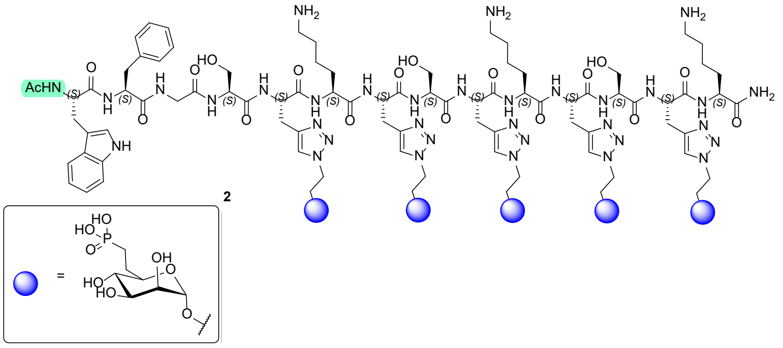

The glycopeptide **2A** (5 mg, 1.3 μmol) was deprotected and purified by the same methodology as for compound **1**. The product **2** was lyophilized, and its identity was confirmed by high-resolution ESI mass spectrometry in a negative mode (found: 3205.1875; calculated: 3205.1886, deconvoluted spectrum) (**Figure S5**) and the purity (≥95%) checked by analytical HPLC (**Figure S6**). The yield was 2.5 mg (60%).

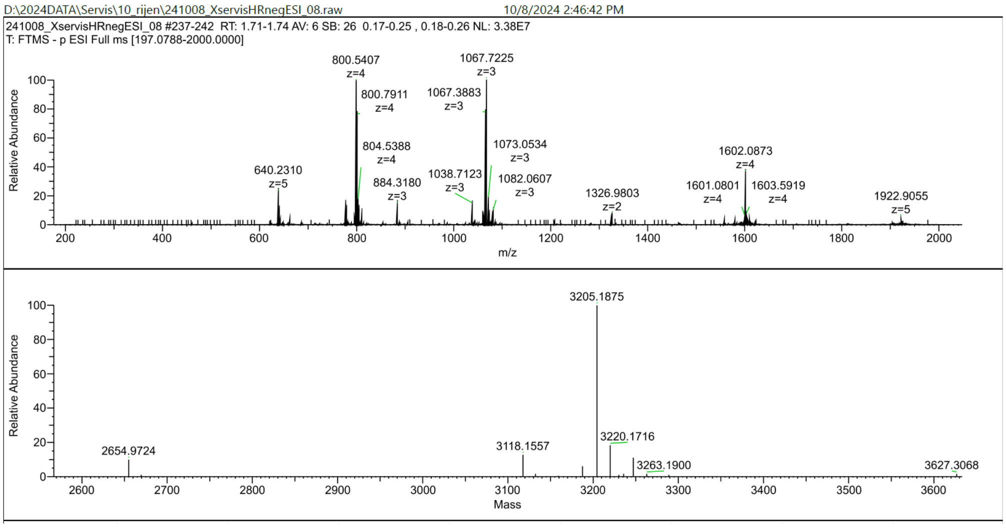

Figure S5. Deconvoluted high-resolution ESI mass spectrum of compound **2** in a negative mode.

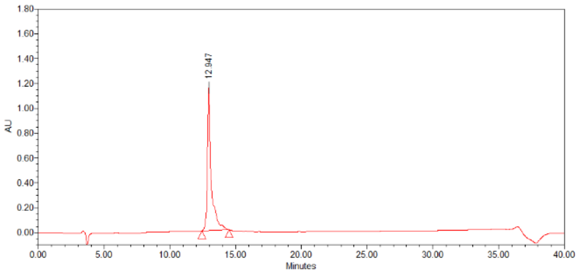

Figure S6. Analytical HPLC chromatogram of compound **2** on C4 column (YMC-Triart Bio C4, 250 × 4 mm) at a flow rate of 1 mL/min using a linear gradient of 0.8% to 80% acetonitrile in water with 0.1% TFA over 30 minutes. Detection at 218 nm.

Peptide P3 ($C_{32}H_{40}N_8O_5$, Exact Mass: 616.3122, Molecular Weight: 616.7230)

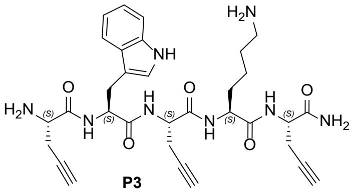

P3

The peptide was prepared using the same methodology as **P1** but using 200 µmol synthetic batch. The product **P3** was lyophilized, and its identity was confirmed by ESI mass spectrometry in a positive mode (found: 617.3 for M^{1+}; calculated: 617.3 for M^{1+}). The yield was 91 mg (74%).

Glycopeptide 3A ($C_{77}H_{112}N_{17}O_{38}P_3$, Exact Mass: 1975.6567, Molecular Weight: 1976.7453)

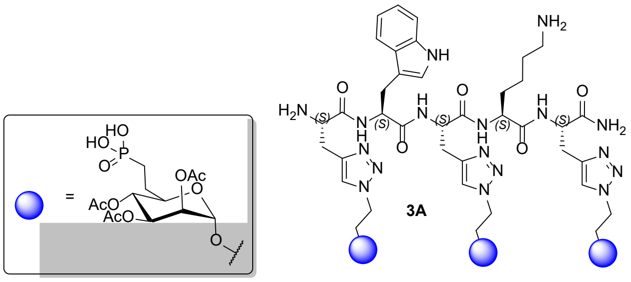

The peptide **P3** (10 mg, 14.3 µmol) was "clicked" with compound **14** by the same methodology as **1A**. The purified product **3A** was lyophilized, and its identity was confirmed by MALDI mass spectrometry in a positive mode (found: 1976.7285 for M^{1+}; calculated: 1976.6647 for M^{1+}). The yield was 21.9 mg (77%).

<u>Compound 3</u> ($C_{59}H_{94}N_{17}O_{29}P_3$, Exact Mass: 1597.5616, Molecular Weight: 1598.4123)

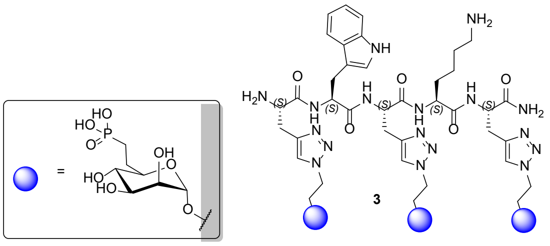

The glycopeptide **3A** (9.6 mg, 4.86 μmol) was deprotected and purified by the same methodology as for compound **1**. Product **3** was lyophilized, and its identity was confirmed by high-resolution ESI mass spectrometry in a negative mode (found: 1597.5625; calculated: 1597.5616, deconvoluted spectrum) (**Figure S7**) and the purity (≥95%) checked by analytical HPLC (**Figure S8**). The yield was 4.2 mg (54%).

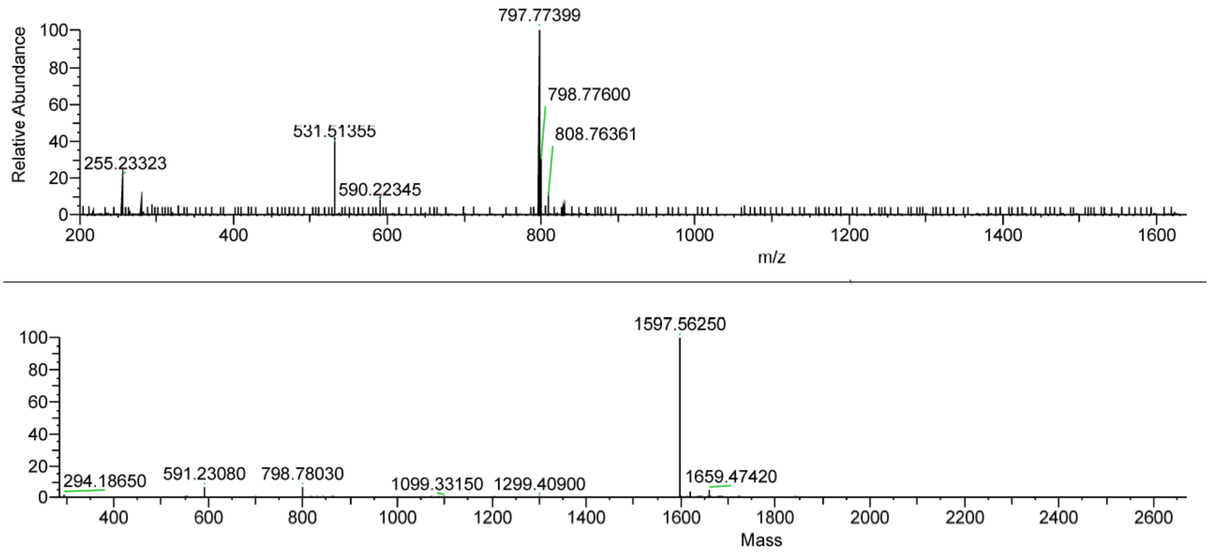

Figure S7. Deconvoluted high-resolution ESI mass spectrum of compound **3** in a negative mode.

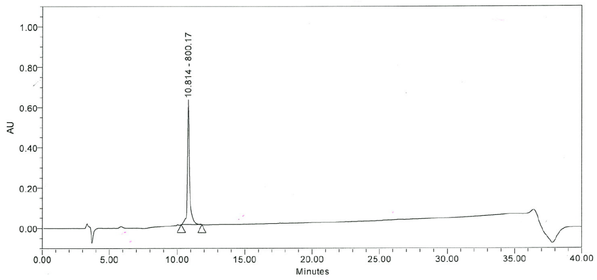

Figure S8. Analytical HPLC chromatogram of compound **3** on C4 column (YMC-Triart Bio C4, 250 × 4 mm) at a flow rate of 1 mL/min using a linear gradient of 0.8% to 80% acetonitrile in water with 0.1% TFA over 30 minutes. Detection at 218 nm.

Compound 4 ($C_{63}H_{98}N_{17}O_{31}P_3$, Exact Mass: 1681.5827, Molecular Weight: 1682.4863)

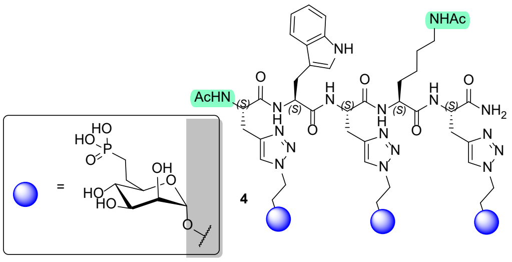

The glycopeptide **3A** (10 mg, 5.06 µmol) was dissolved in 1 mL of ACN and 0.35 mL of DIPEA (2 mmol) and 0.35 mL of Ac_2O (3.7 mmol) were added. After 30 minutes of stirring at ambient temperature, the reaction mixture was evaporated to dryness, and the residue was dissolved in 1.5 mL of dry MeOH. Then, solid CH_3ONa was added to form a saturated, highly basic solution (confirmed by pH indicator paper). The completeness of the reaction was checked by LC-MS. The crude product was purified using the same method as for peptide **P1**. The product **4** was lyophilized, and its identity was confirmed by high-resolution MALDI mass spectrometry in a negative mode (found: 1680.5730 for M^{1-}; calculated: 1680.5747 for M^{1-}) (**Figure S9**) and the purity (≥95%) checked by analytical HPLC (**Figure S10**). The yield was 4 mg (44%).

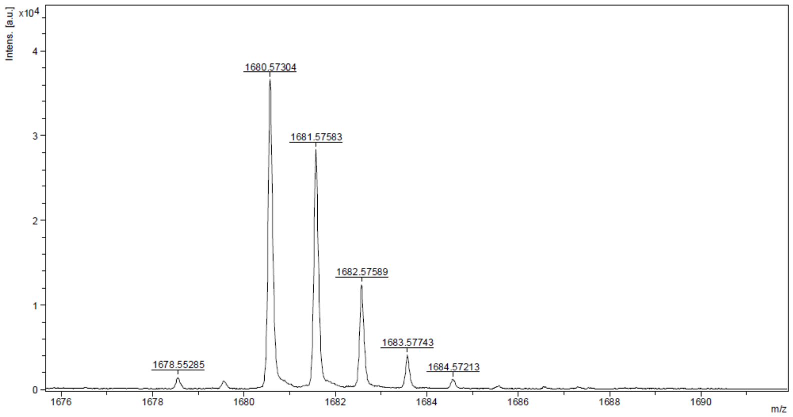

Figure S9. High-resolution MALDI mass spectrum of compound **4** in a negative mode.

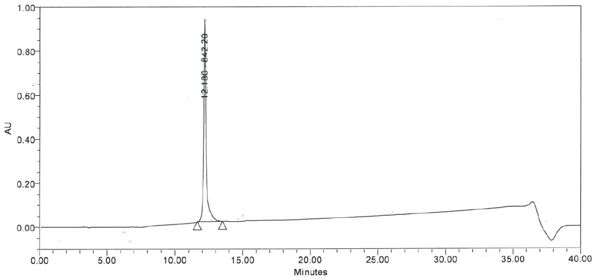

Figure S10. Analytical HPLC chromatogram of compound **4** on C4 column (YMC-Triart Bio C4, 250 × 4 mm) at a flow rate of 1 ml/min using a linear gradient of 0.8% to 80% acetonitrile in water with 0.1% TFA over 30 minutes. Detection at 218 nm.

Peptide P4 ($C_{21}H_{23}N_5O_3$, Exact Mass: 393.1801, Molecular Weight: 393.4470)

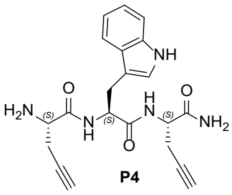

The peptide **P4** was prepared using the same methodology as **P1** but using 200 μmol synthetic batch. The product **P4** was lyophilized, and its identity was confirmed by MALDI mass spectrometry in positive mode (found: 394.1873 for M^{1+}; calculated: 394.1881 for M^{1+}). The yield was 63 mg (51%).

Glycopeptide 5A ($C_{51}H_{71}N_{11}O_{25}P_2$, Exact Mass: 1299.4098, Molecular Weight: 1300.1285)

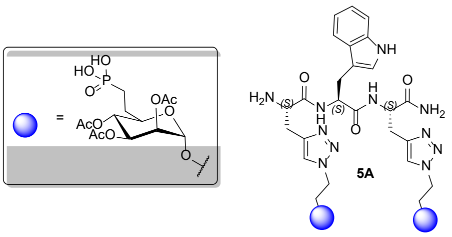

The peptide **P4** (10 mg, 25.4 μmol) was "clicked" with **compound 14** by the same methodology as **1A**. The purified product **5A** was lyophilized, and its identity was confirmed by MALDI mass spectrometry in a positive mode (found: 1300.4284 for M^{1+}; calculated: 1300.4178 for M^{1+}). The yield was 16 mg (48%).

<u>Compound 5</u> ($C_{39}H_{59}N_{11}O_{19}P_2$, Exact Mass: 1047.3464, Molecular Weight: 1047.9065)

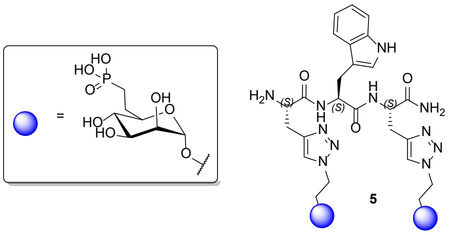

The **5A** peptide (7 mg, 5.4 μmol) was deprotected and purified by the same methodology as for compound **1**. Product **5** was lyophilized, and its identity was confirmed by high-resolution MALDI mass spectrometry in a positive mode (found: 1048.3549 for M^{1+}; calculated: 1048.3544 for M^{1+}) (**Figure S11**) and the purity (≥95%) checked by analytical HPLC (**Figure S12**). The yield was 2.8 mg (50%).

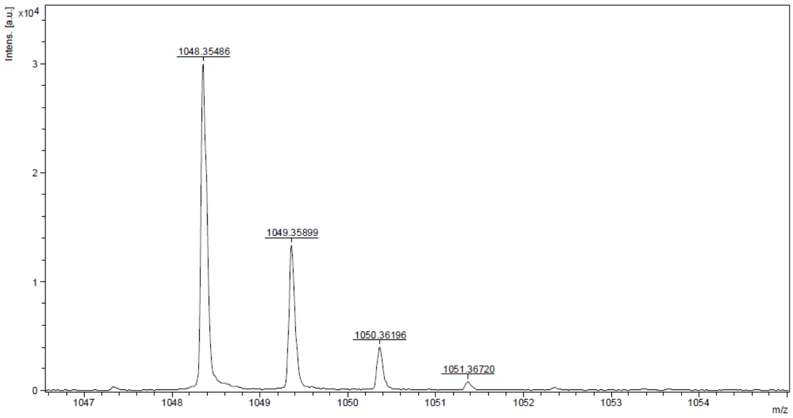

Figure S11. High-resolution MALDI mass spectrum of compound **5** in a positive mode.

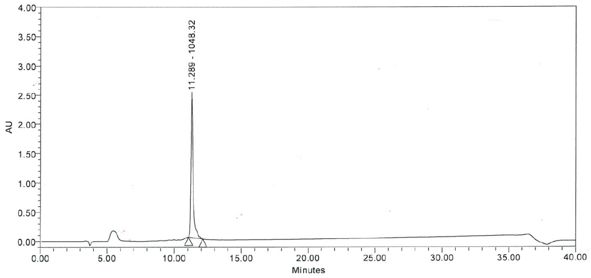

Figure S12. Analytical HPLC chromatogram of compound **5** on C4 column (YMC-Triart Bio C4, 250 × 4 mm) at a flow rate of 1 mL/min using a linear gradient of 0.8% to 80% acetonitrile in water with 0.1% TFA over 30 minutes. Detection at 218 nm.

Compound 6 ($C_{41}H_{61}N_{11}O_{20}P_2$, Exact Mass: 1089.3570, Molecular Weight: 1089.9435)

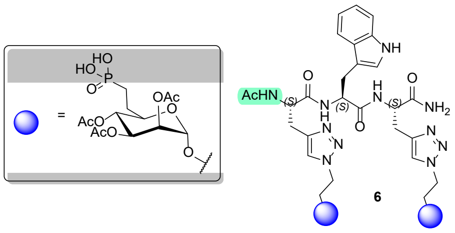

6

Glycopeptide **5A** (7.5 mg, 5.66 μmol) was acetylated and deprotected as described for compound **4**. The crude product **6** was purified using the same method as for compound **1**. The product **6** was lyophilized, and its identity was confirmed by high-resolution MALDI mass spectrometry in a positive mode (found: 1090.3657 for M^{1+}; calculated: 1090.365 for M^{1+}) (**Figure S13**) and the purity (≥95%) checked by analytical HPLC (**Figure S14**). The yield was 3 mg (48%).

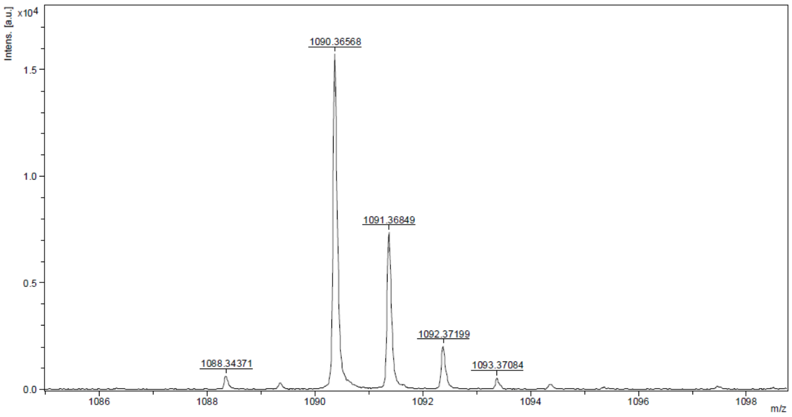

Figure S13. High-resolution MALDI mass spectrum of compound **6** in a negative mode.

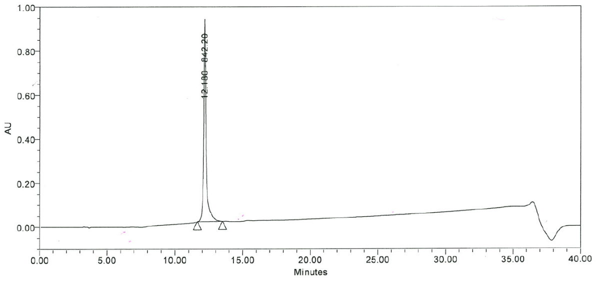

Figure S14. Analytical HPLC chromatogram of compound **6** on C4 column (YMC-Triart Bio C4, 250 × 4 mm) at a flow rate of 1 mL/min using a linear gradient of 0.8% to 80% acetonitrile in water with 0.1% TFA over 30 minutes. Detection at 218 nm.

Affinity modelling

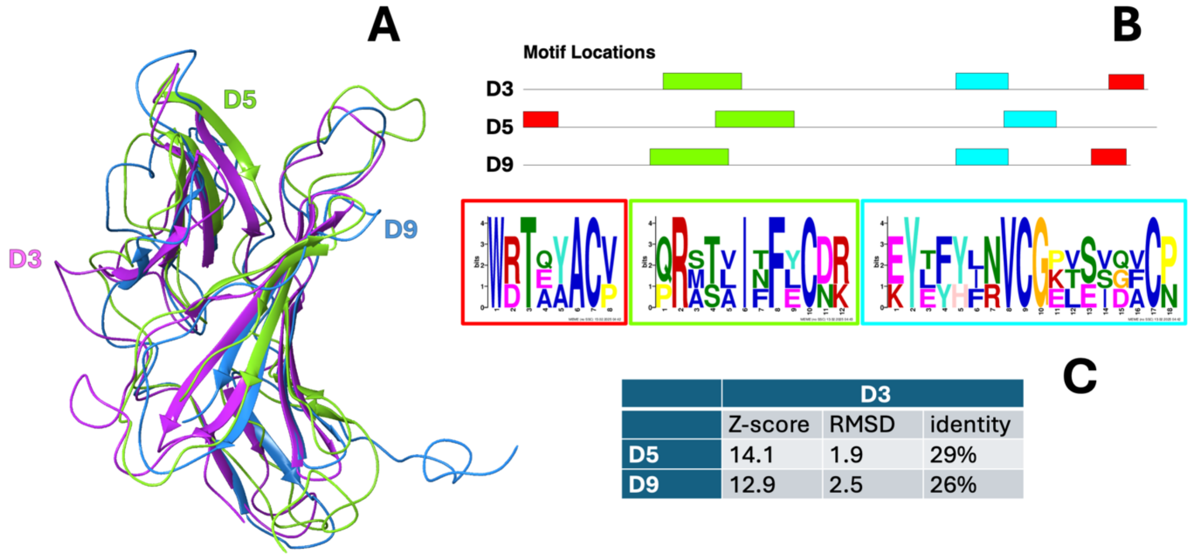

	D3		
	Z-score	RMSD	identity
D5	14.1	1.9	29%
D9	12.9	2.5	26%

Figure S15. Comparison of the prepared D3, D5, and D9 IGF2R models. A: Structural overlap; B: Sequence motif analysis (MEME Suite, Ref.[49]); C: Domain structural similarity analysis (DALI, Ref.[50]).

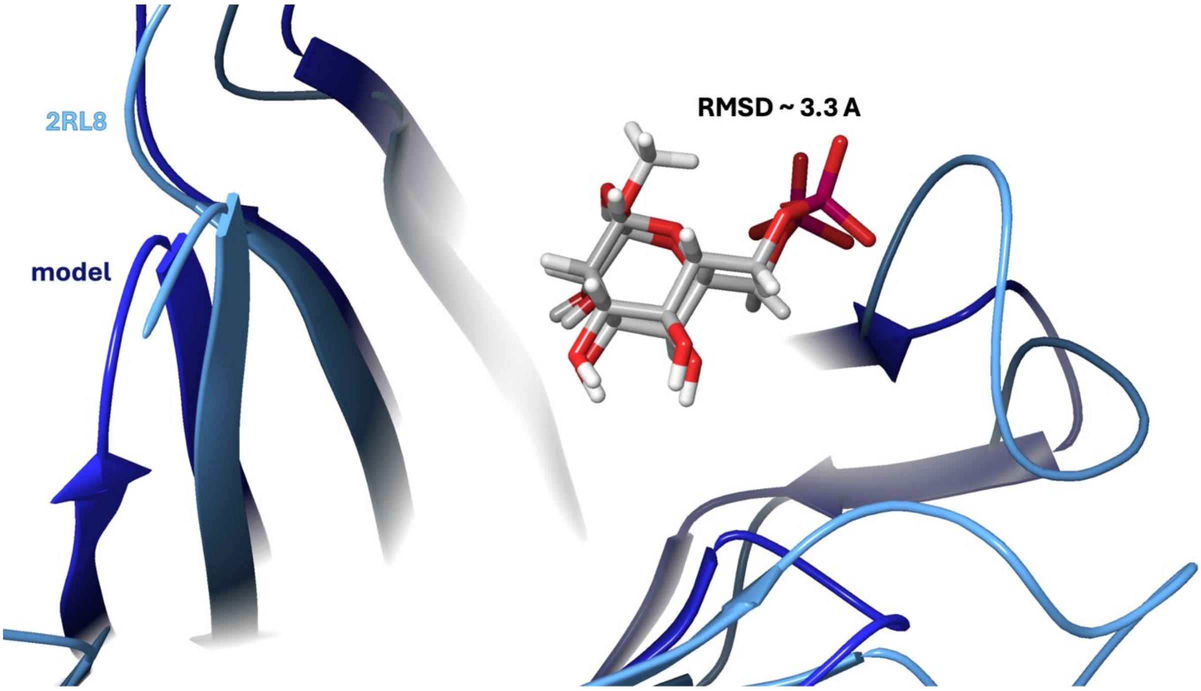

Figure S16. Comparison of the best docking pose (according to E_{model}) of compound **7** to D9 with the experimental structure of 2RL8.

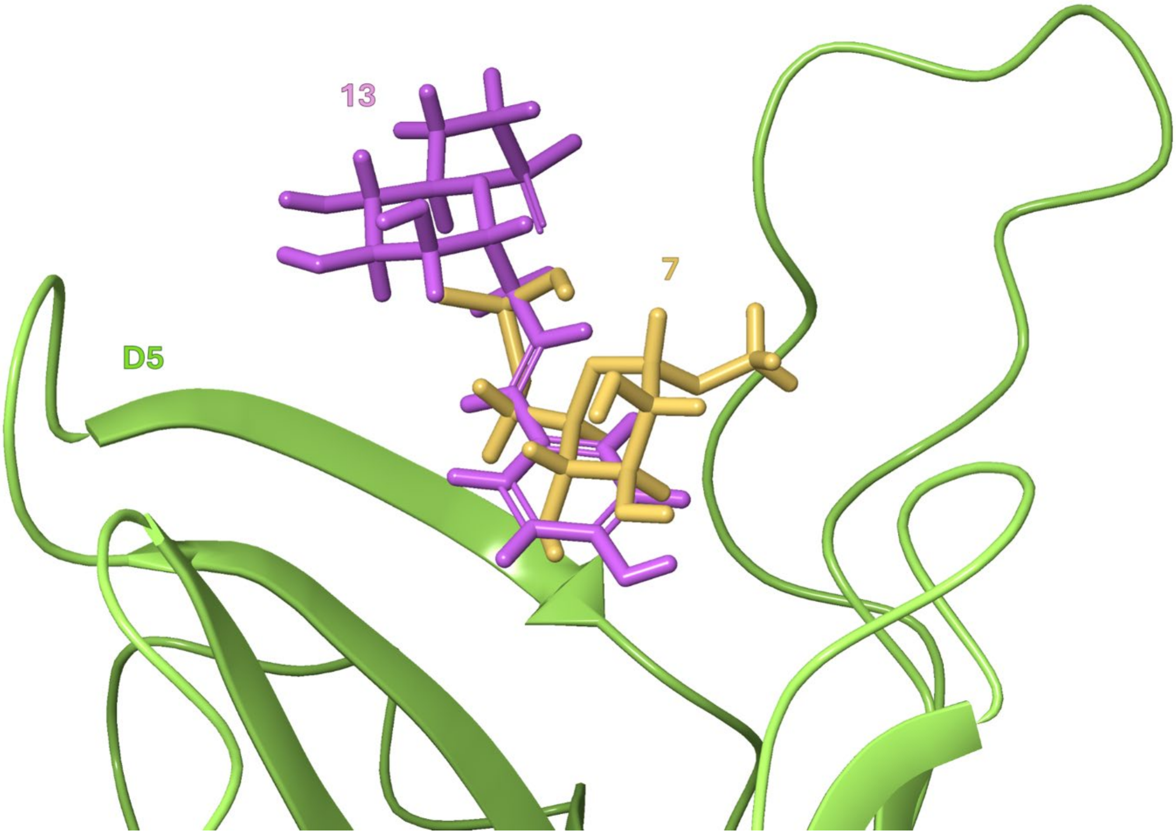

Figure S17. Comparison of the best docking pose (according to MM-GBSA) of compound **13** to D5 with the best pose of compound **7** (M6P).

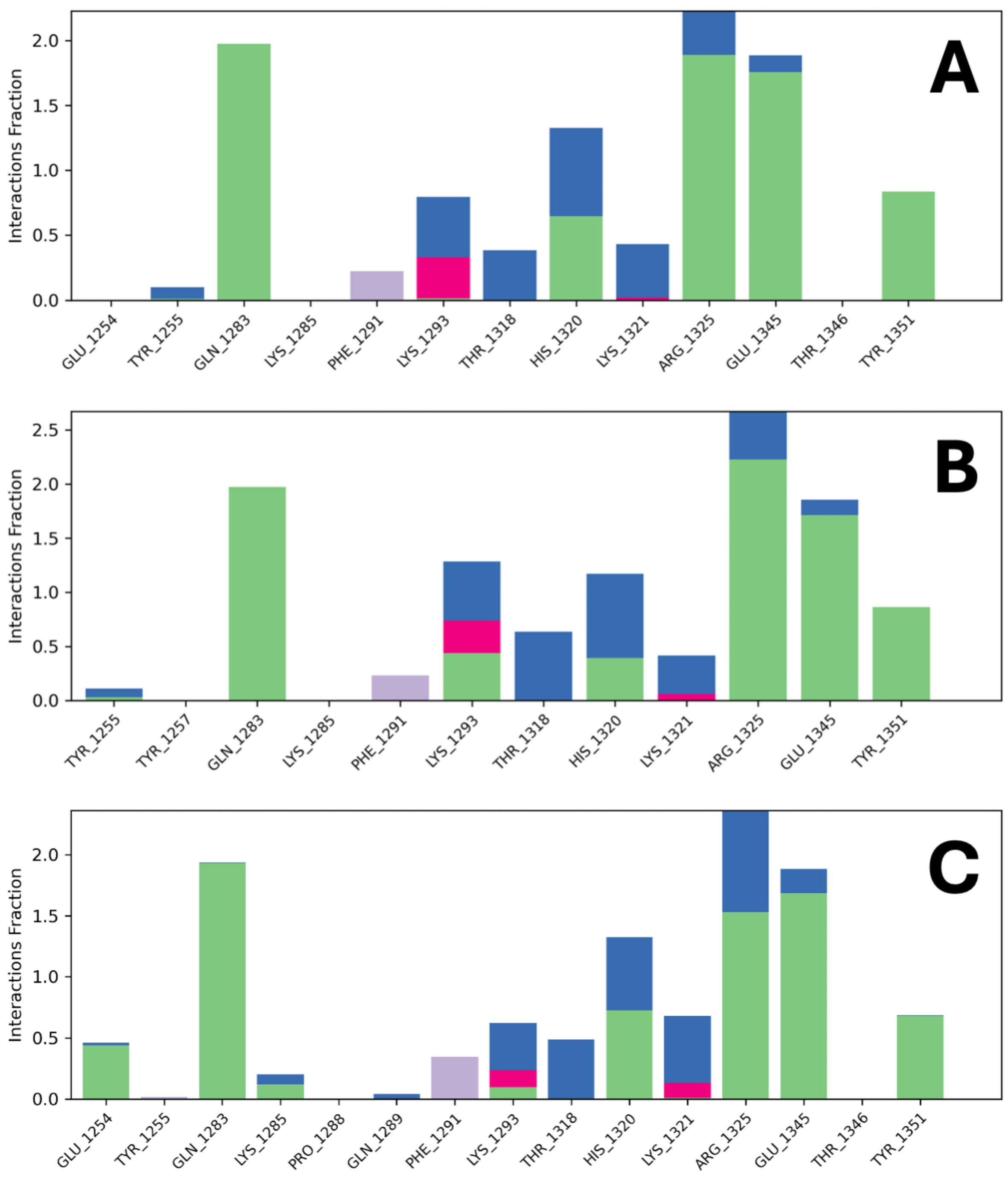

Figure S18. Interactions of M6P **7** (A), compound **8** (B), and compound **13** (C) with D9-IGF2R according to their type (hydrogen bonds - green; hydrophobic - purple; ionic - magenta; water bridges - blue). The stacked bar charts are normalized over the course of the (500 ns) MD trajectory.

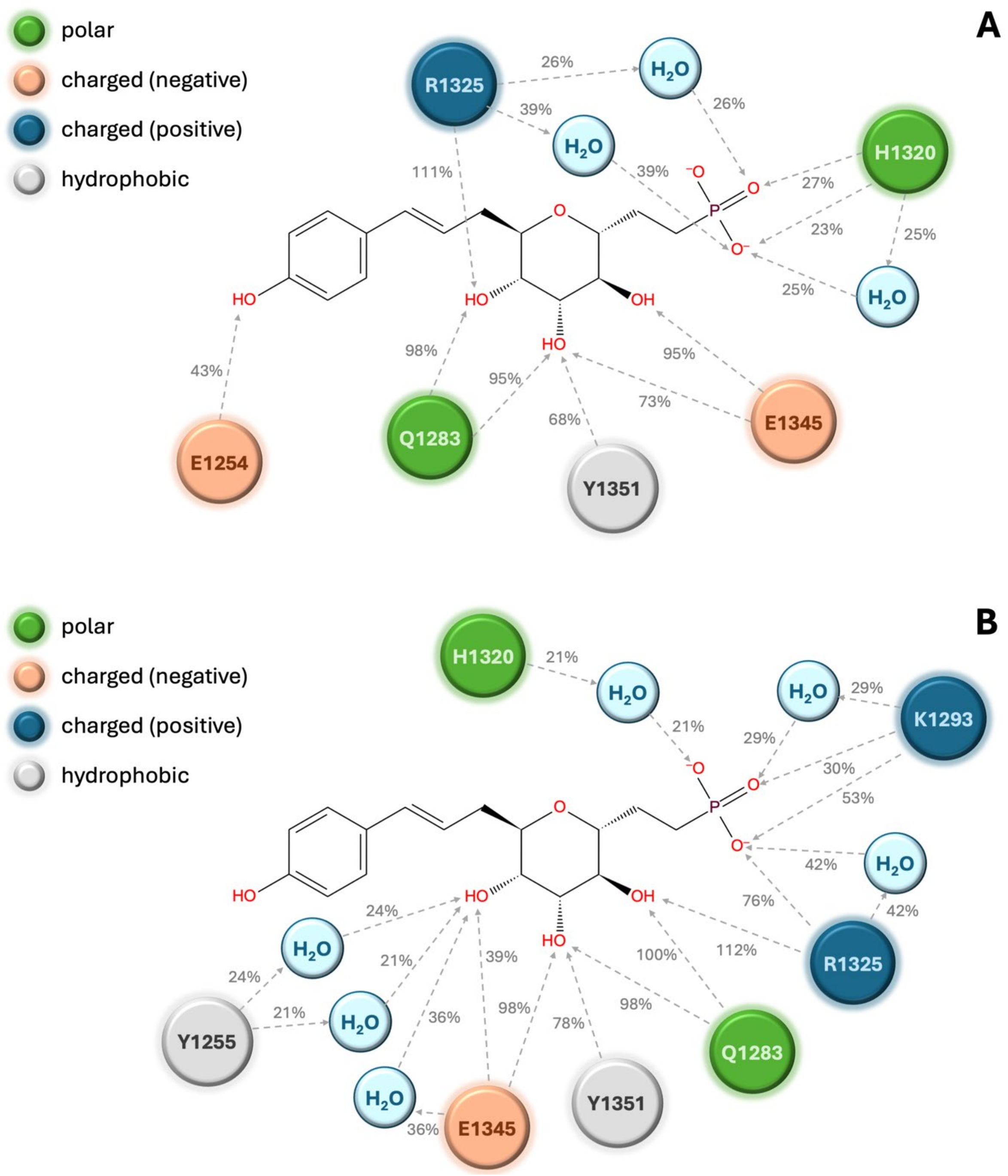

Figure S19. Interactions of compound **13** atoms with D9 protein residues. Only interactions that occur more than 30.0% of the simulation time (500 ns) are shown. Since arginine 1325 can have multiple interactions of the same type with the same ligand atom, interactions > 100% are the result. Two different orientations of **13** in D9 are displayed. A: orientation corresponding to **7** in D9; B: possible flipped orientation.

Table S1. Comparison of docking affinity predictions (in terms of E_{model}, GlideScores; in kcal/mol) and MM-GBSA predictions of the best poses of compounds **7-13** to IGF2(D3), IGF2(D5) and IGF2(D9) with experimental IC_{50} (μM).

Compound	IC_{50}	D3			D5			D9		
		E_{model}	GlideScore	MM-GBSA	E_{model}	GlideScore	MM-GBSA	E_{model}	GlideScore	MM-GBSA
**7**	14.0	-72.0	-6.2	-36.0	-54.7	-6.4	-27.5	-70.1	-6.2	-37.7
**8**	11.5	-70.6	-6.2	-23.8	-46.3	-5.6	-20.1	-66.5	-6.2	-35.0
**9**	126.9	-70.5	-6.4	-29.9	-47.6	-5.7	-22.7	-63.2	-6.4	-30.0
**10**	1000.0[a]	-40.7	-4.7	-37.9	-38.4	-4.9	-39.3	-49.5	-5.7	-45.3
**11**	89.2	-56.4	-5.9	-36.2	-39.1	-5.3	-34.8	-53.8	-6.1	-39.1
**12**	1000.0[a]	-41.3	-5.1	-41.0	-37.3	-4.9	-38.7	-44.1	-5.0	-40.0
**13**	4.4	-70.3	-5.5	-30.9	-62.5	-5.6	-50.8	-62.0	-5.4	-45.1
Pearson		0.94	0.84	-0.69	0.68	0.80	-0.33	0.86	0.64	-0.42
Pearson[b]		0.42	-0.46	-0.20	0.61	0.37	0.31	0.51	-0.56	0.63

[a]Since the experimental affinity was unmeasurably low, it was replaced by the arbitrary value of 1000 μM for comparison with the calculation.

[b]Pearson's correlation without outliers (compounds **10** and **12**).

Table S2. Comparison of docking affinity predictions (in terms of E_{model}, GlideScores; in kcal/mol) and MM-GBSA predictions of the best poses of compounds **7-13** to IGF2R[a] with experimental IC_{50} (μM).

**Compound**	**IC_{50}**	**IGF2R**		
		E_{model}	GlideScore	MM-GBSA
**7**	14.0	-72.0	-6.4	-37.7
**8**	11.5	-70.6	-6.2	-35.0
**9**	126.9	-70.5	-6.4	-30.0
**10**	1000.0[b]	-49.5	-5.7	-45.3
**11**	89.2	-56.4	-6.1	-39.1
**12**	1000.0[b]	-44.1	-5.1	-41.0
**13**	4.4	-70.3	-5.6	-50.8
Pearson		0.90	0.70	-0.27
Pearson[c]		0.42	-0.43	0.60

[a]The table shows the lowest binding energy for a given ligand to all considered IGF2R domains. The color then demonstrates which domain the ligand should bind best to according to the given method: D3 (magenta), D5 (green), and D9 (blue)

[b]Since the experimental affinity was unmeasurably low, it was replaced by the arbitrary value of 1000 μM for comparison with the calculation.

[c]Pearson's correlation without outliers (compounds **10** and **12**)

Table S3. Comparison of ΔG (in kcal/mol; MD-GBSA) and ΔH (kcal/mol; semiempirical) of compounds **7-13** to IGF2(D9) with experimental IC_{50} (μM).

**Compound**	**IC_{50}**	**ΔG**	**ΔH**
**7**	14.0	-33.4	-17.6
**8**	11.5	-34.6	-23.3
**9**	126.9	-21.5	-18.8
**10**	1000.0[a]	-13.2	-10.5
**11**	89.2	-33.3	-24.7
**12**	1000.0[a]	-13.2	-11.3
**13**	4.4	-41.9	-5.3
Pearson		0.89	0.44
Pearson[b]		0.83	0.44

[a]Since the experimental affinity was unmeasurably low, it was replaced by the arbitrary value of 1000 μM for comparison with the calculation.

[b]Pearson's correlation without outliers (compounds **10** and **12**).

Copies of 1H, ^{13}C or APT and ^{31}P NMR Spectra

Methyl 6-deoxy-6-phosphonomethyl-α-D-mannopyranoside bis(triethylammonium) salt (8)

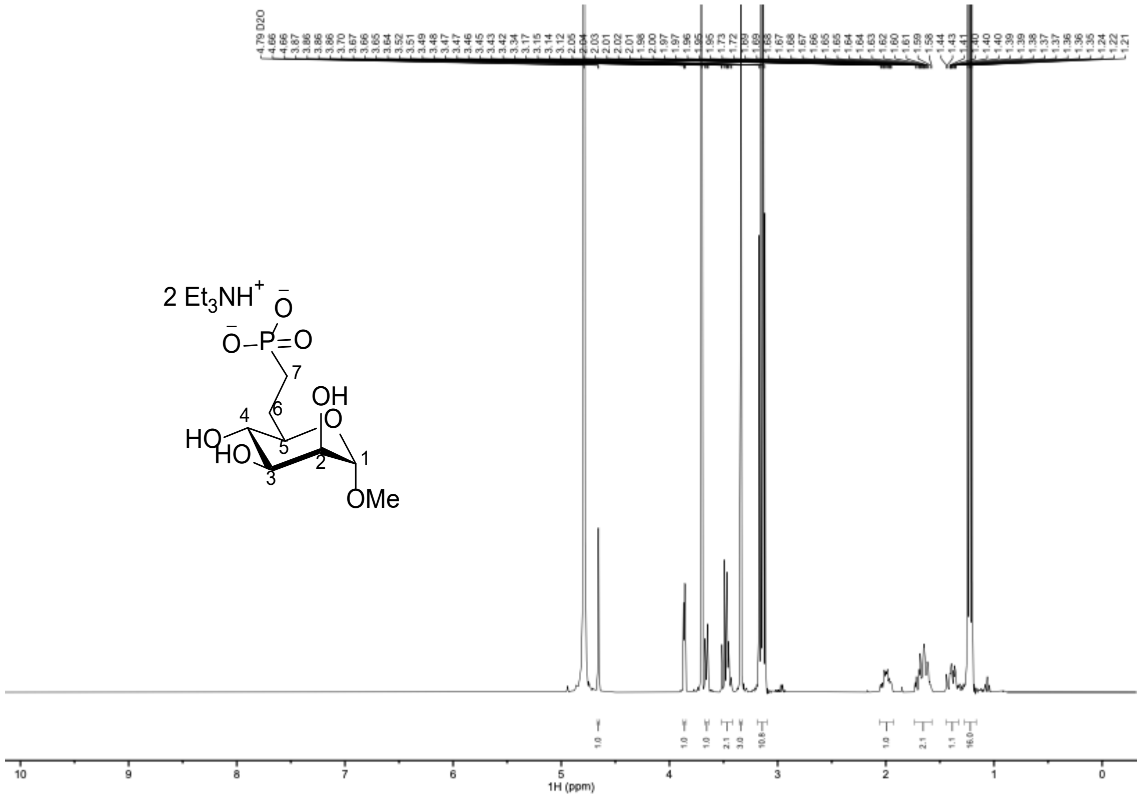

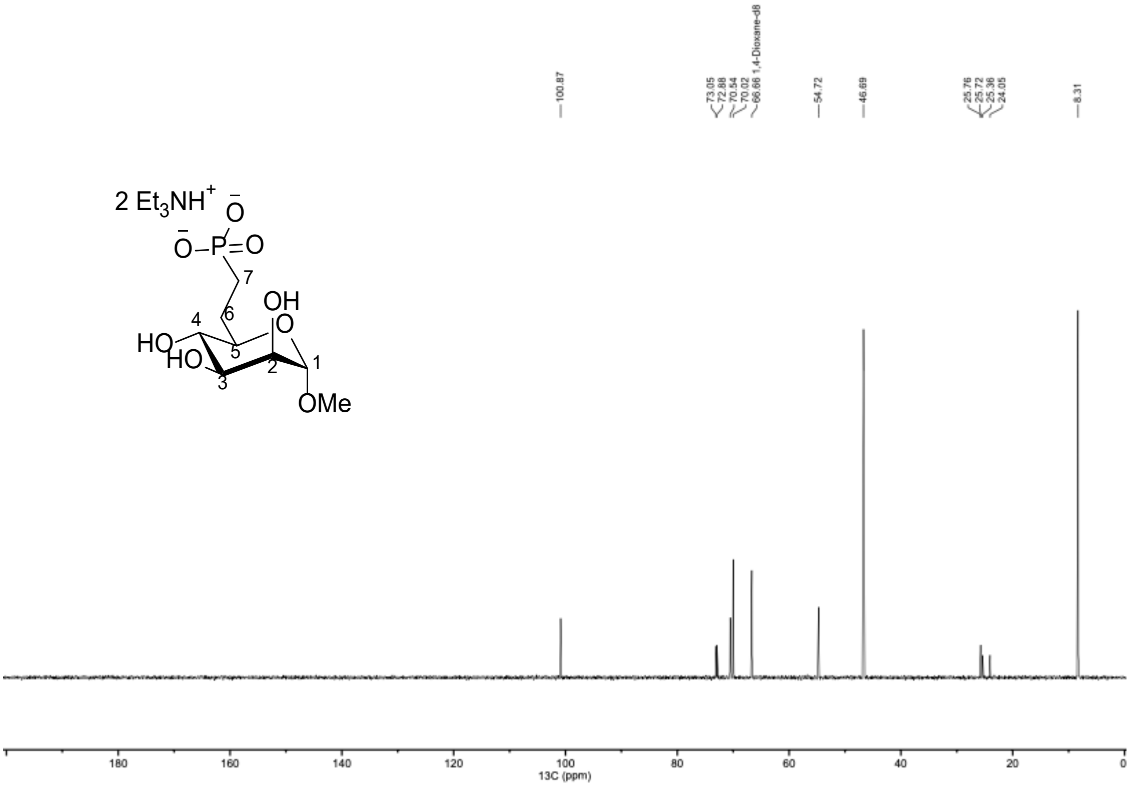

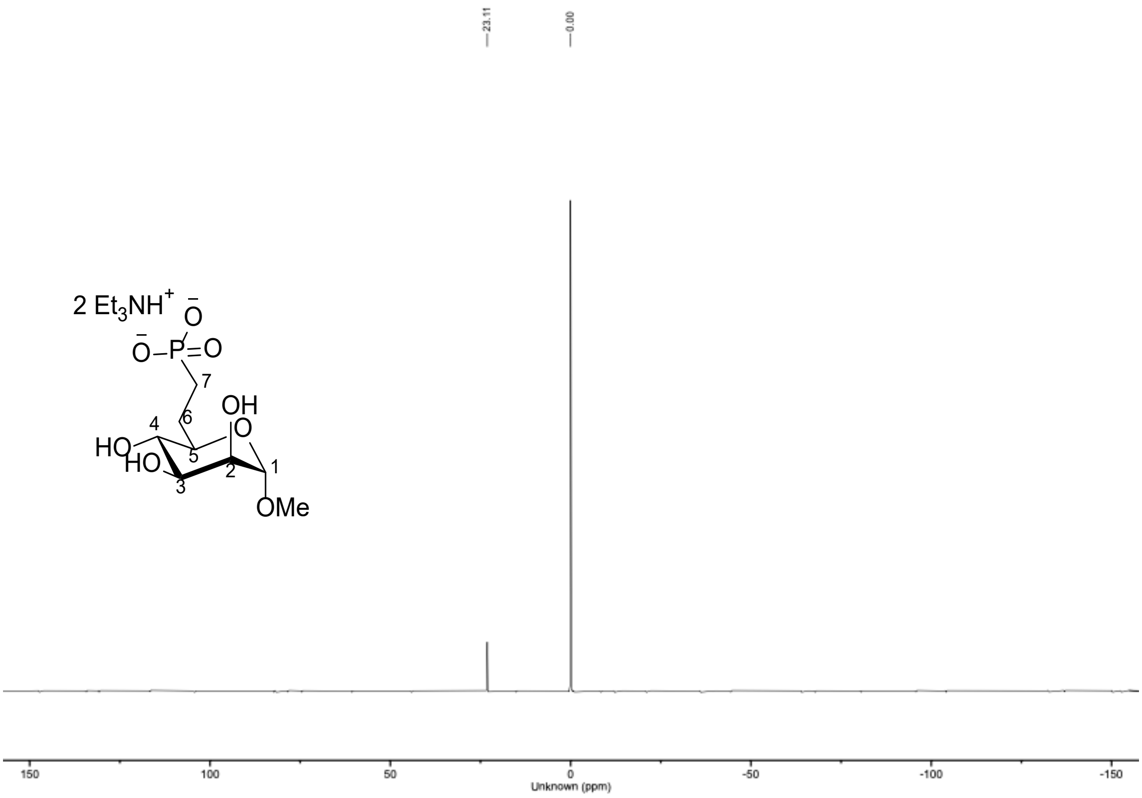
23.11
0.00
2 Et3NH+
O
O–P=O
7
6
OH
O
4
HO
HO
5
2
1
3
OMe
150
100
50
0
-50
-100
-150
Unknown (ppm)

Methyl 6-deoxy-6-phosphono-α-D-mannopyranoside bis(triethylammonium) salt (9)

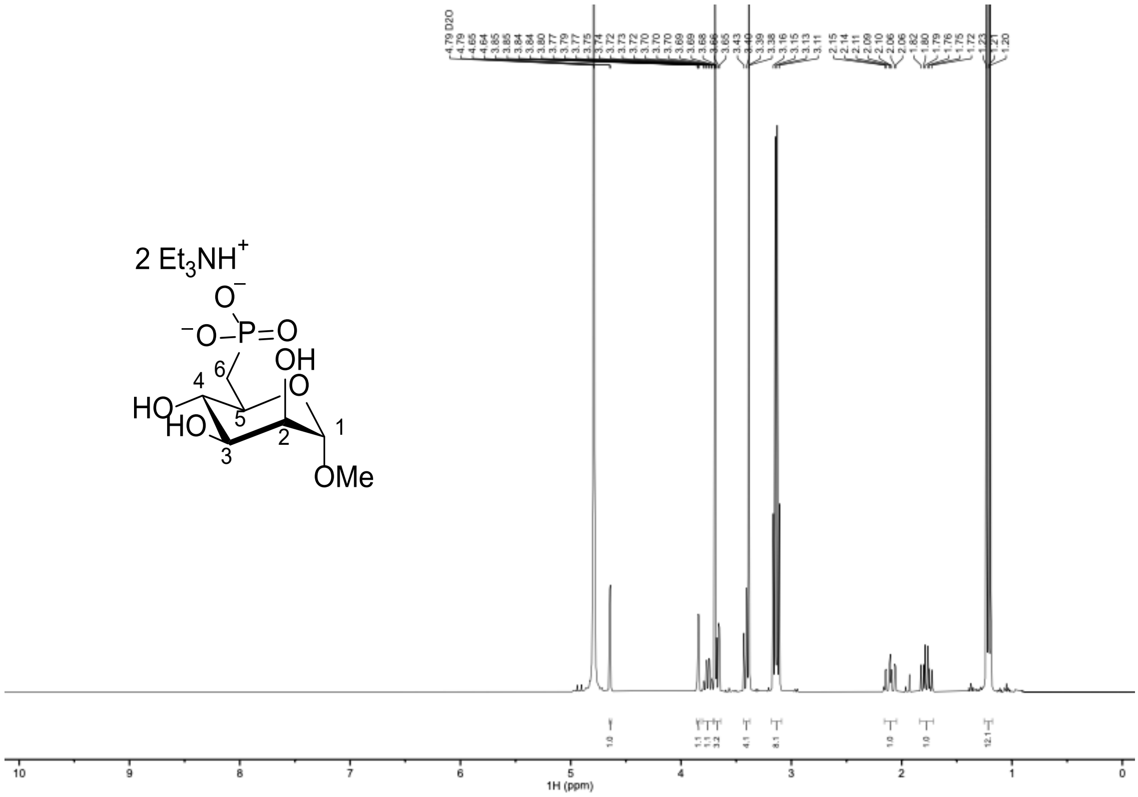

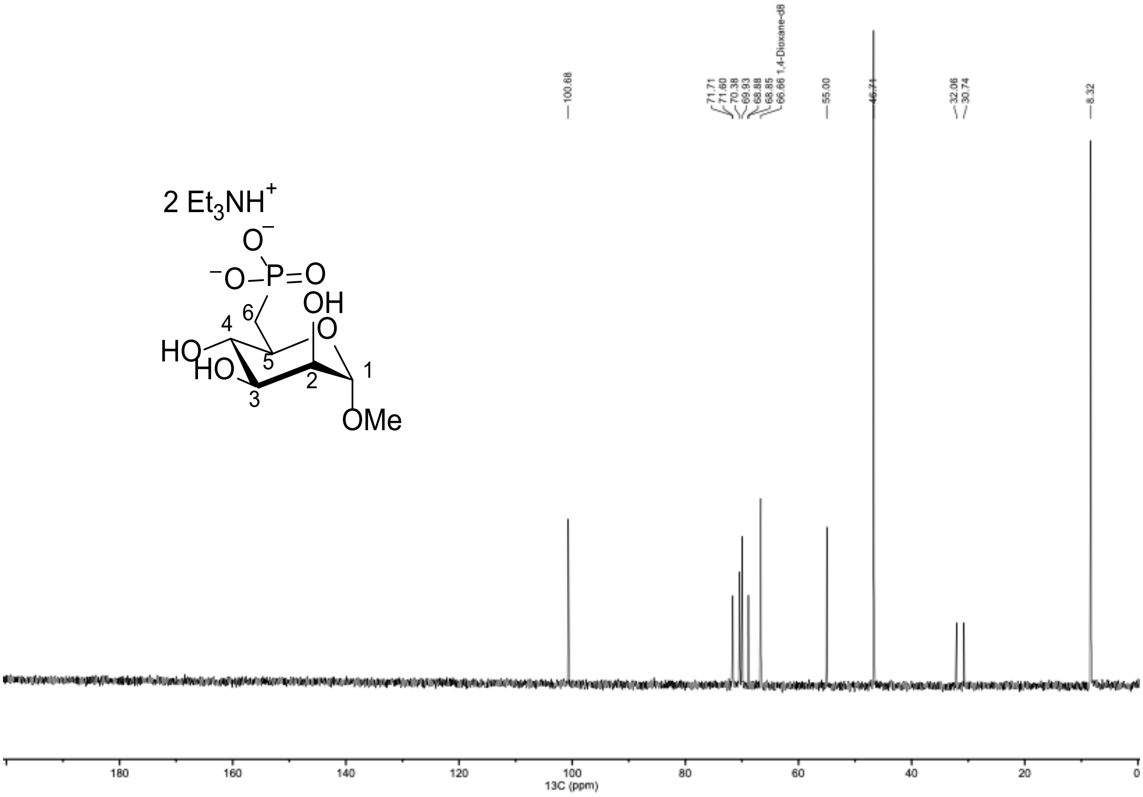

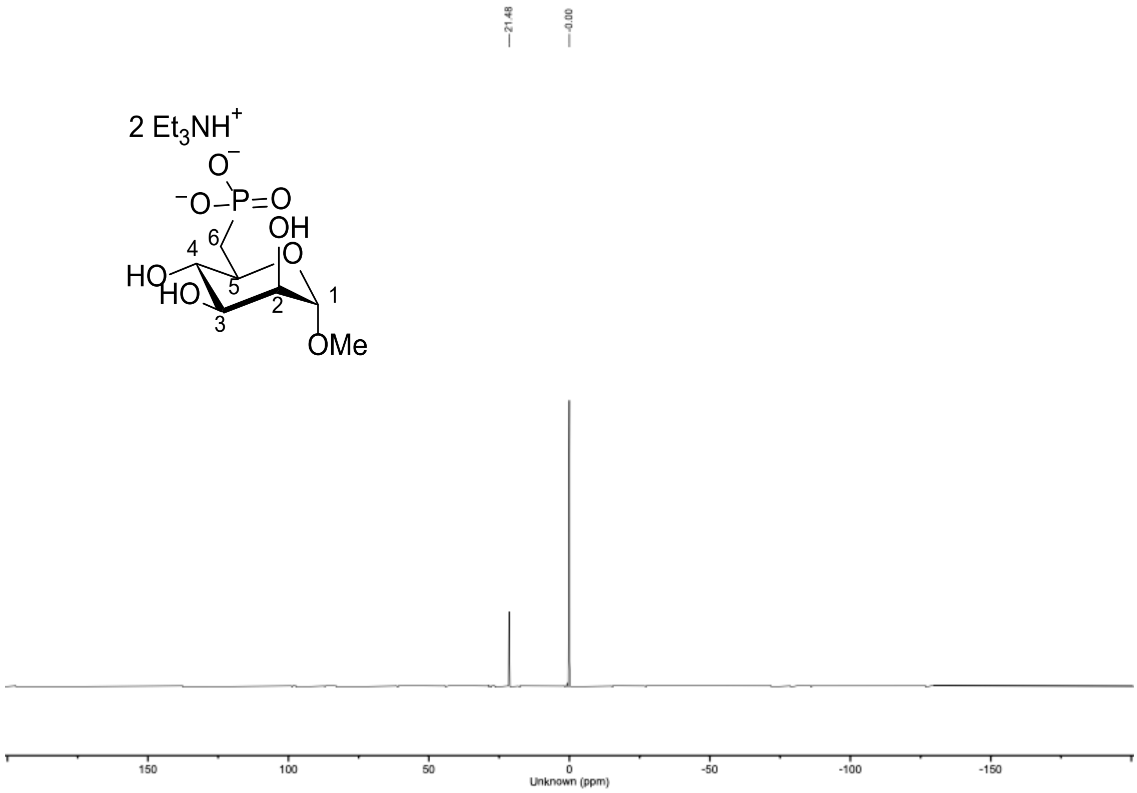

2 Et3NH+
O−
−O−P=O
OH
6
4
O
HO
HO
5
1
2
3
OMe
—21.48
—0.00
150
100
50
0
-50
-100
-150
Unknown (ppm)

Methyl 2,3,4-tri-*O*-benzyl-6,7-dideoxy-7-cyano-α-D-*manno*-heptopyranoside (C)

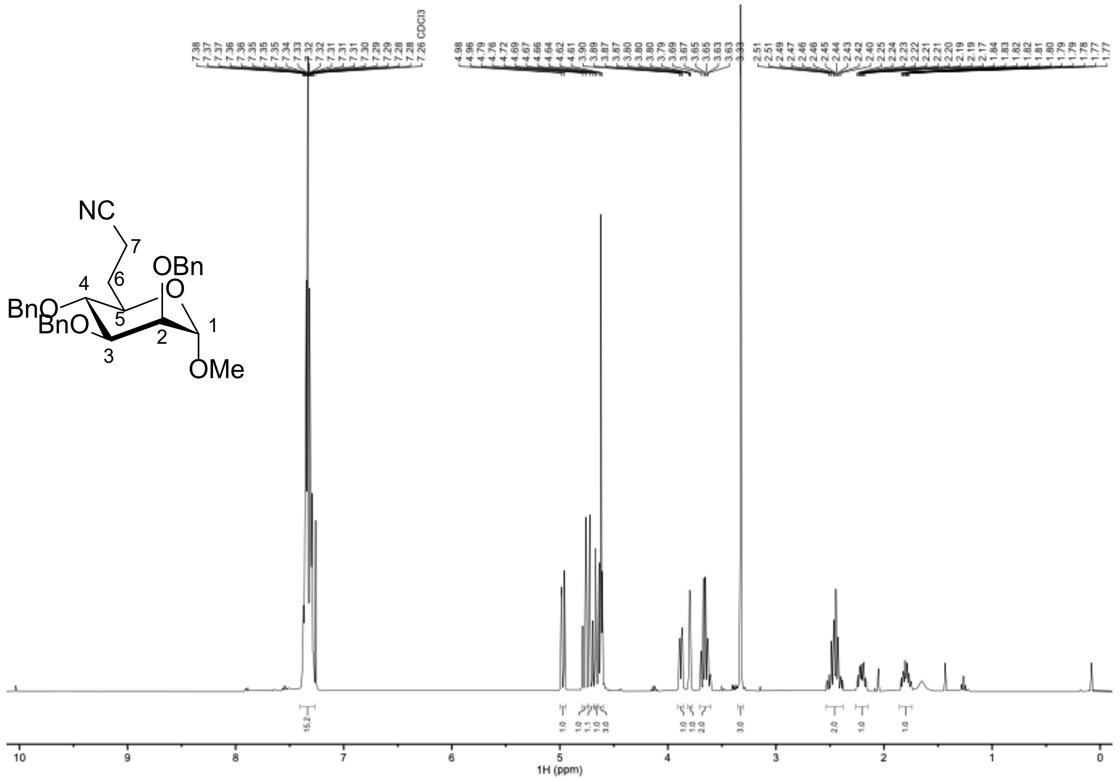

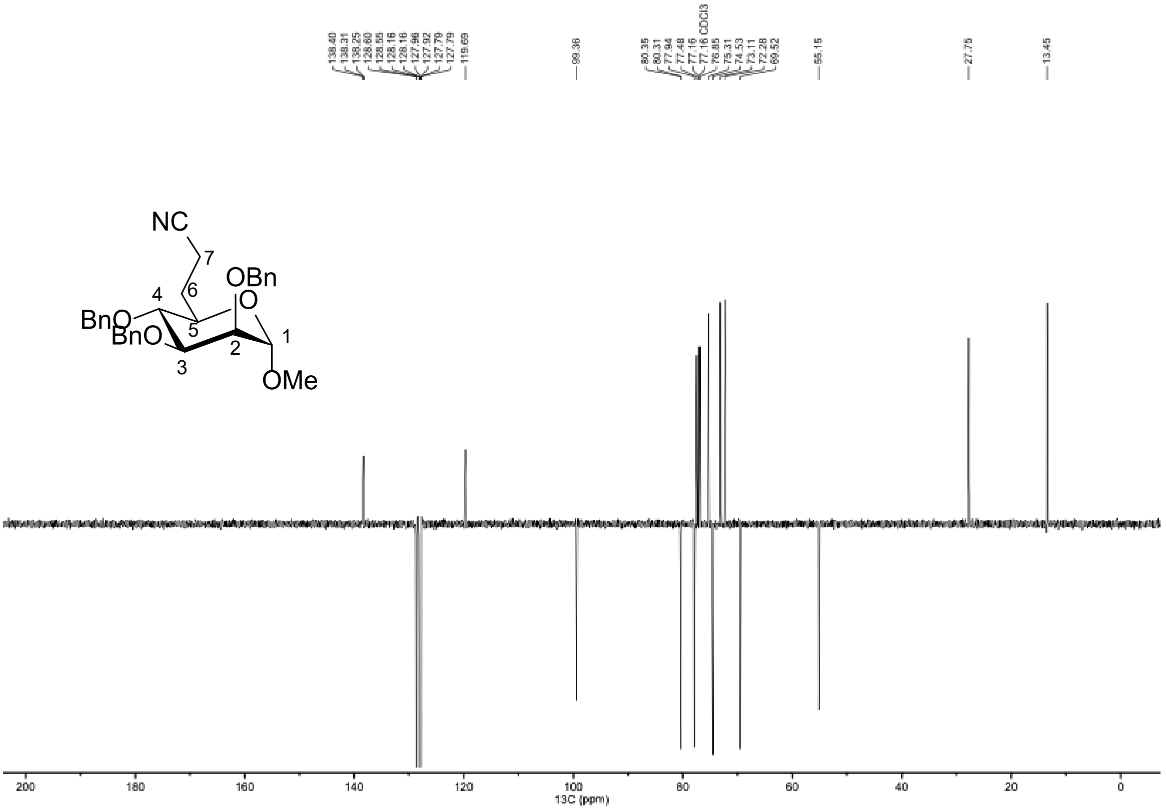

5-(Methyl 2,3,4-tri-*O*-benzyl-6,7-dideoxy-α-D-*manno*-heptapyranos-7-yl)tetrazole (IX)

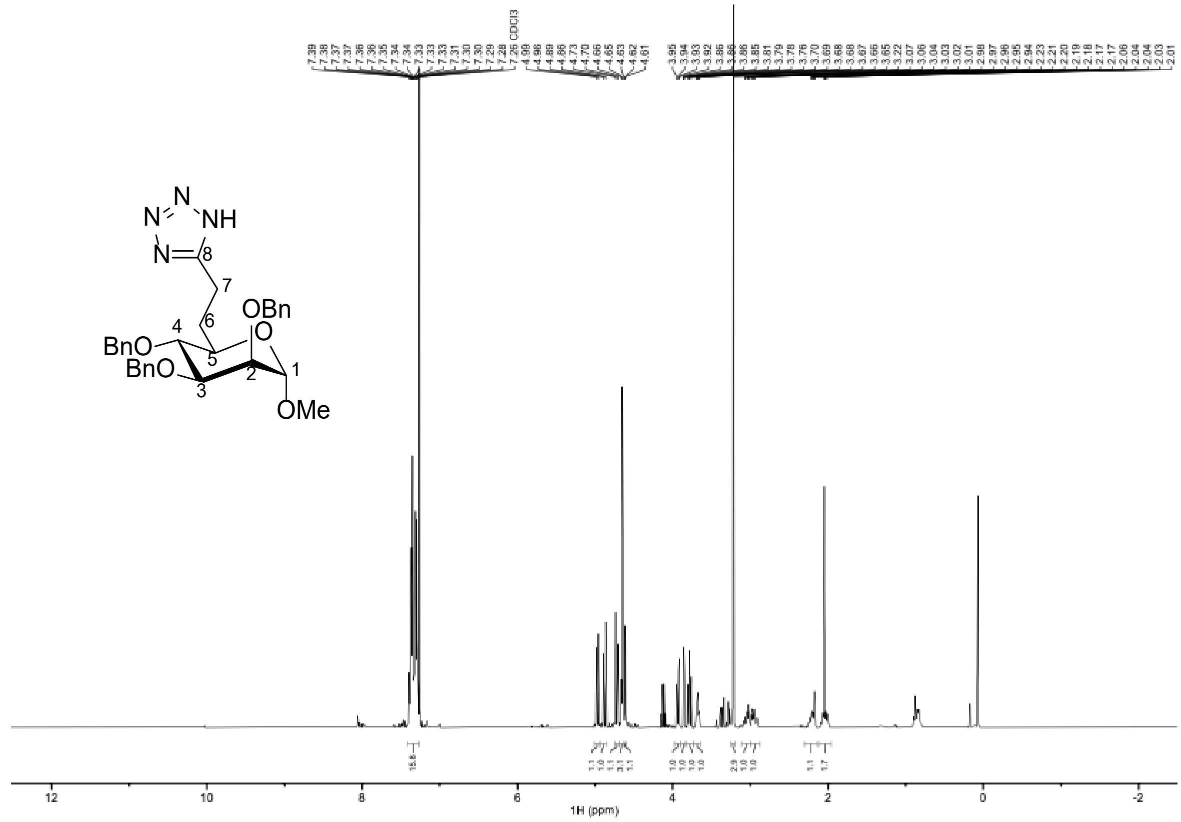

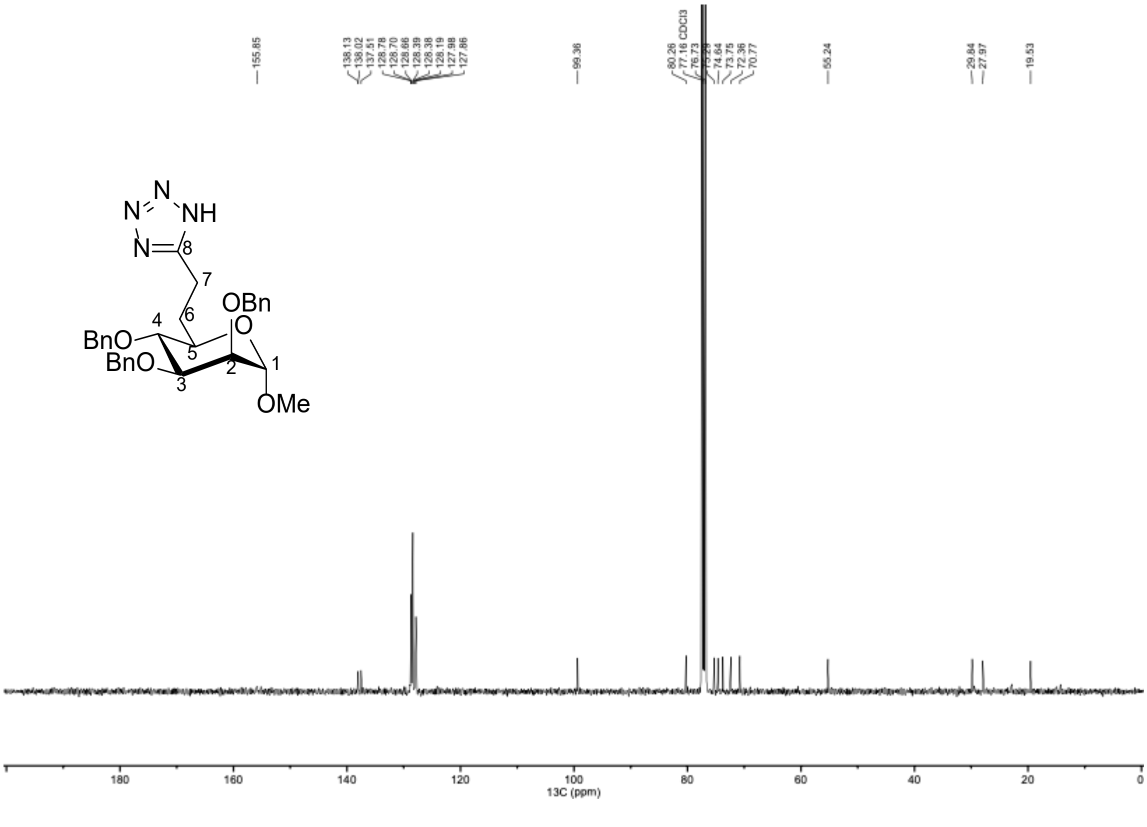

5-(Methyl 6,7-deoxy-α-D-*manno*-heptapyranos-7-yl)tetrazole (10)

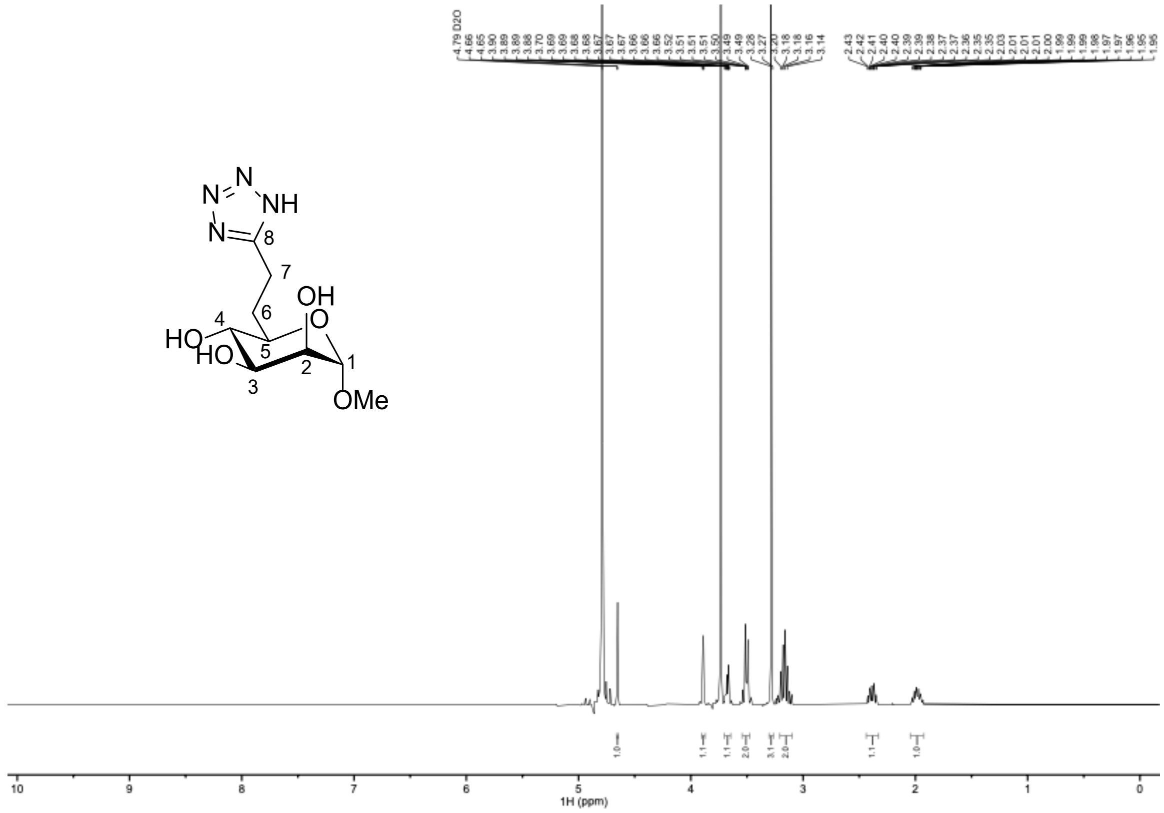

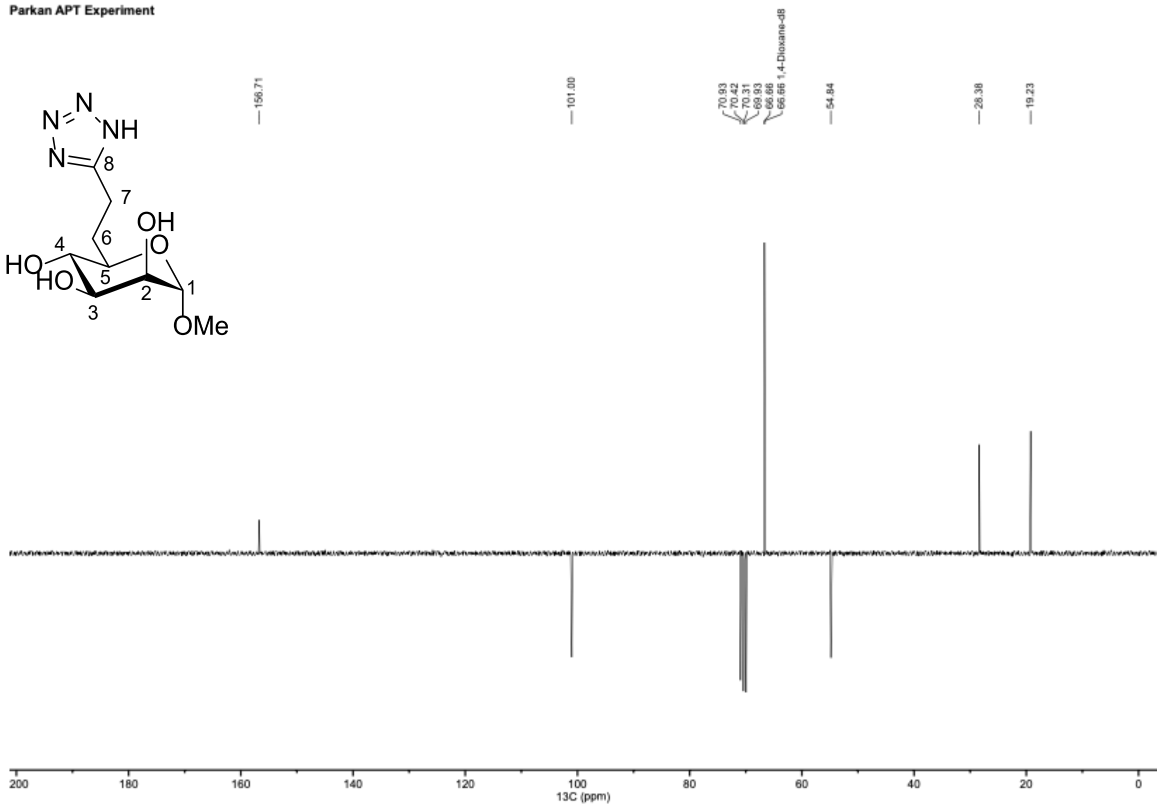

(Methyl 6,7-dideoxy-α-D-*manno*-octopyranoside)uronic acid (11)

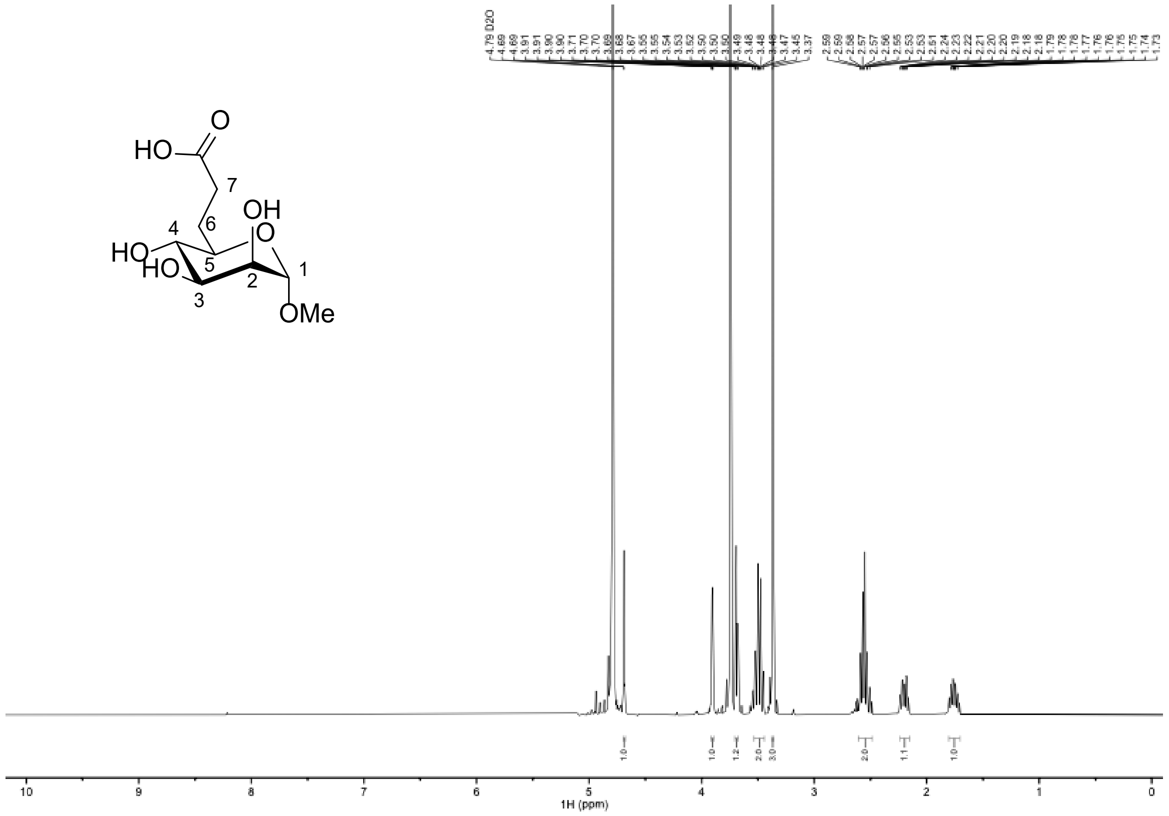

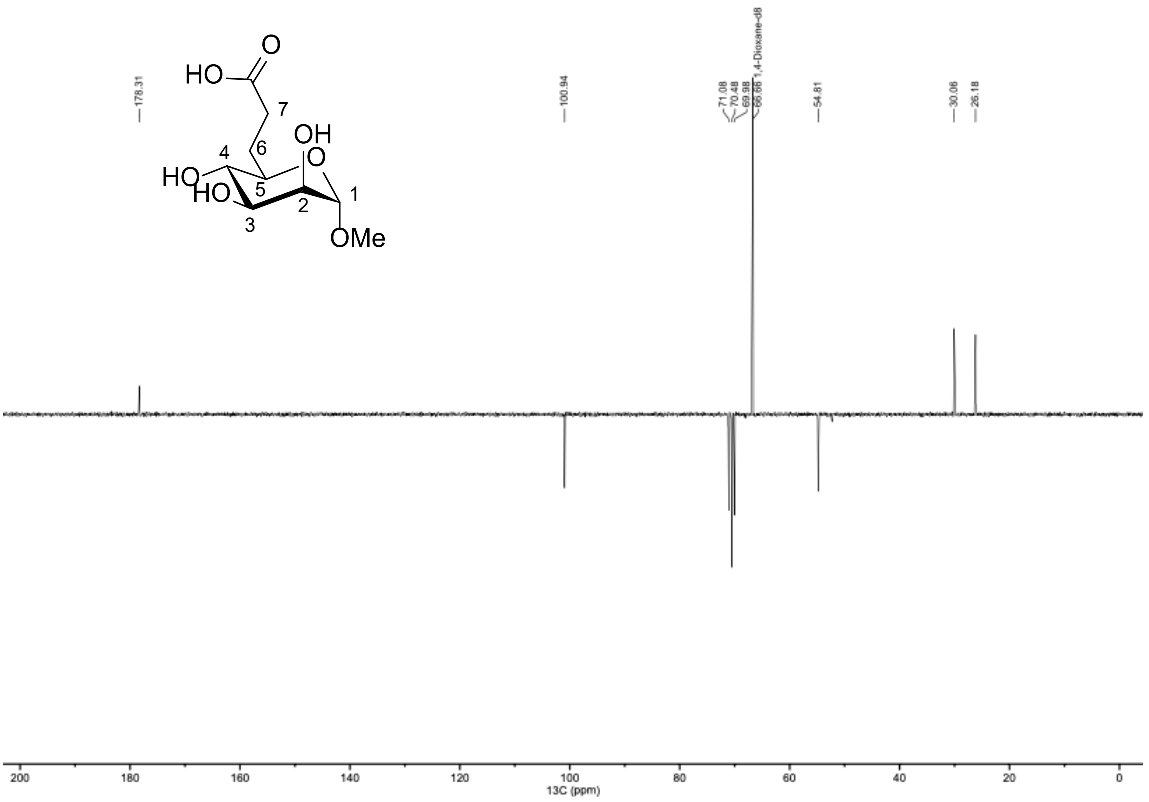

5-(Methyl 6-deoxy-α-D-mannopyranos-6-yl)tetrazole (12)

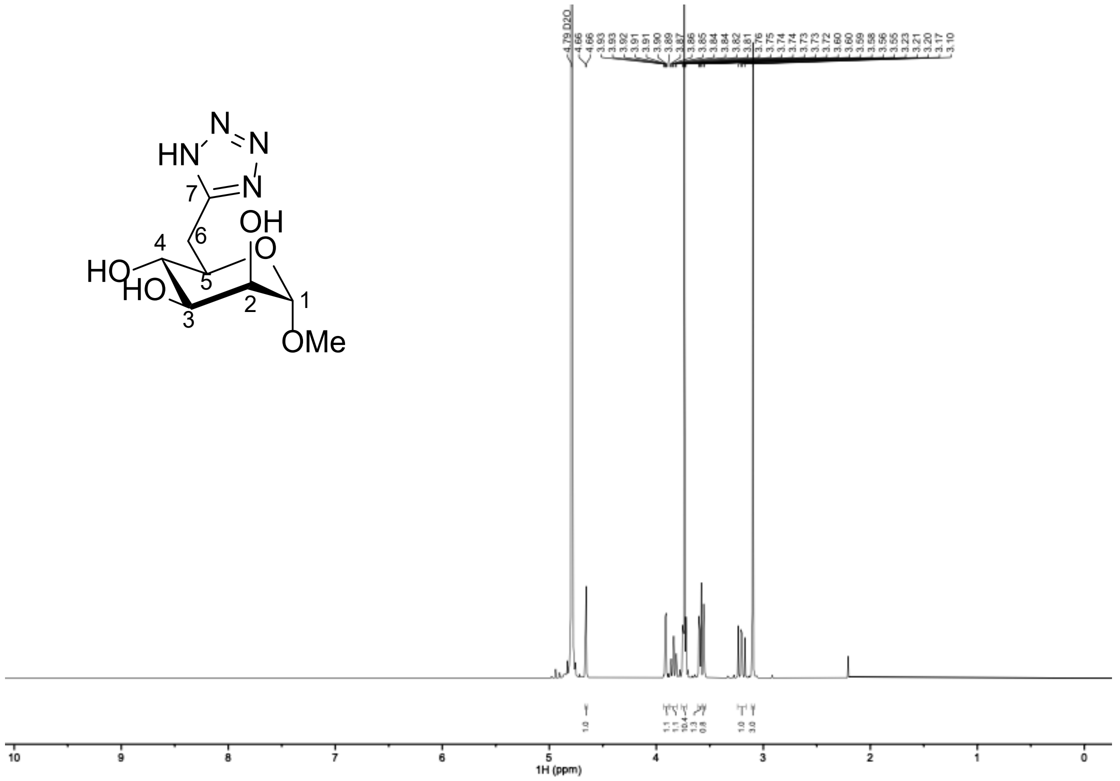

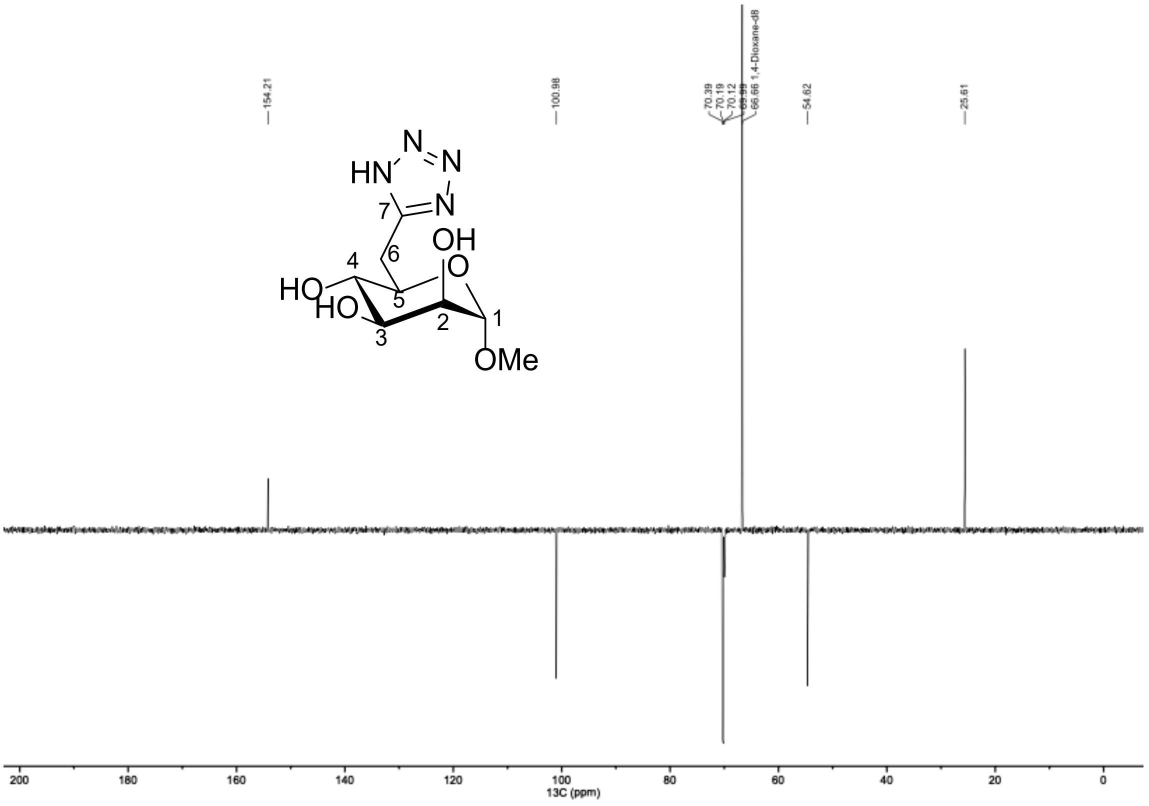

Methyl 2,3,4-tri-*O*-acetyl-6-deoxy-6-(diethylphosphonomethyl)-α-D-mannopyranoside (XIII)

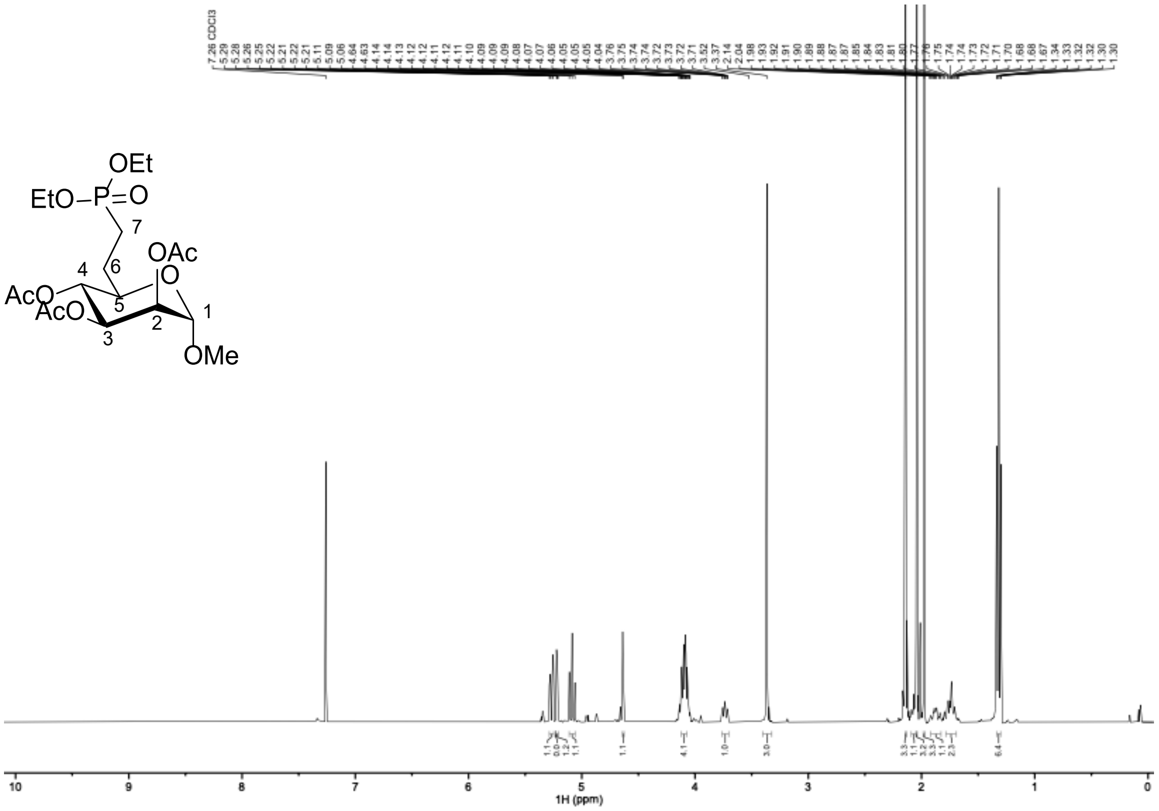

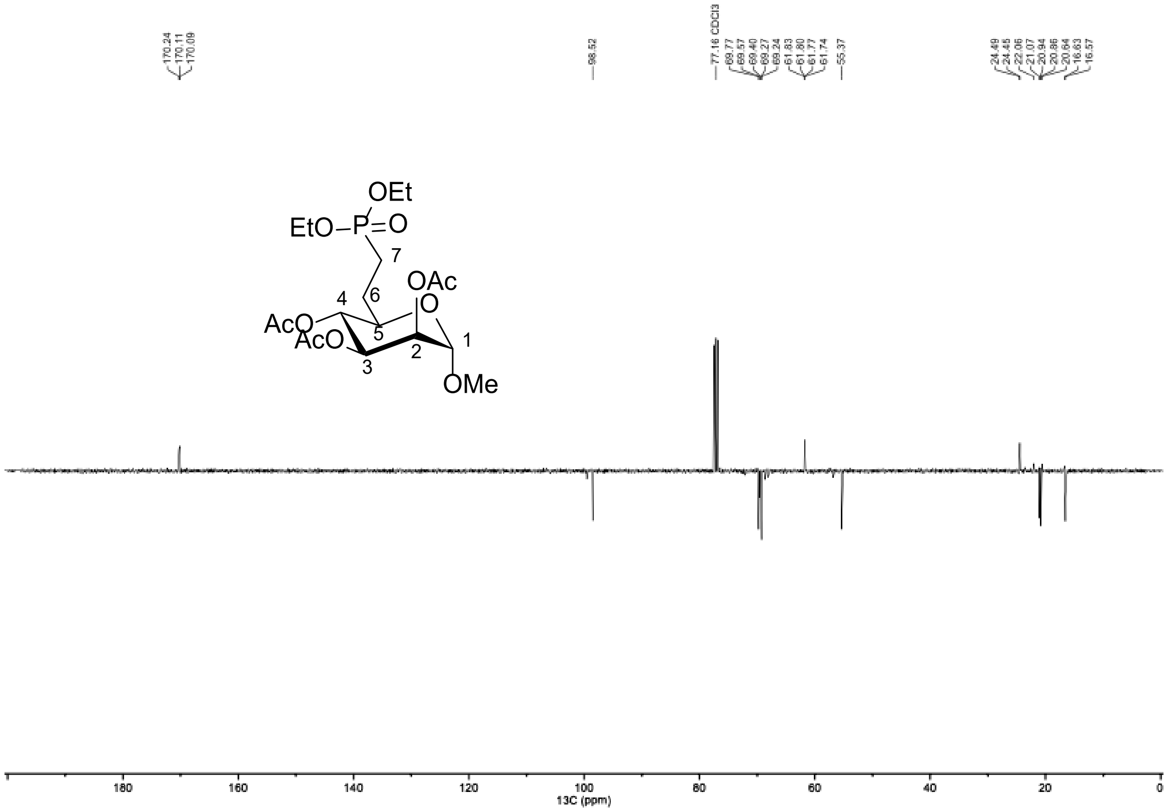

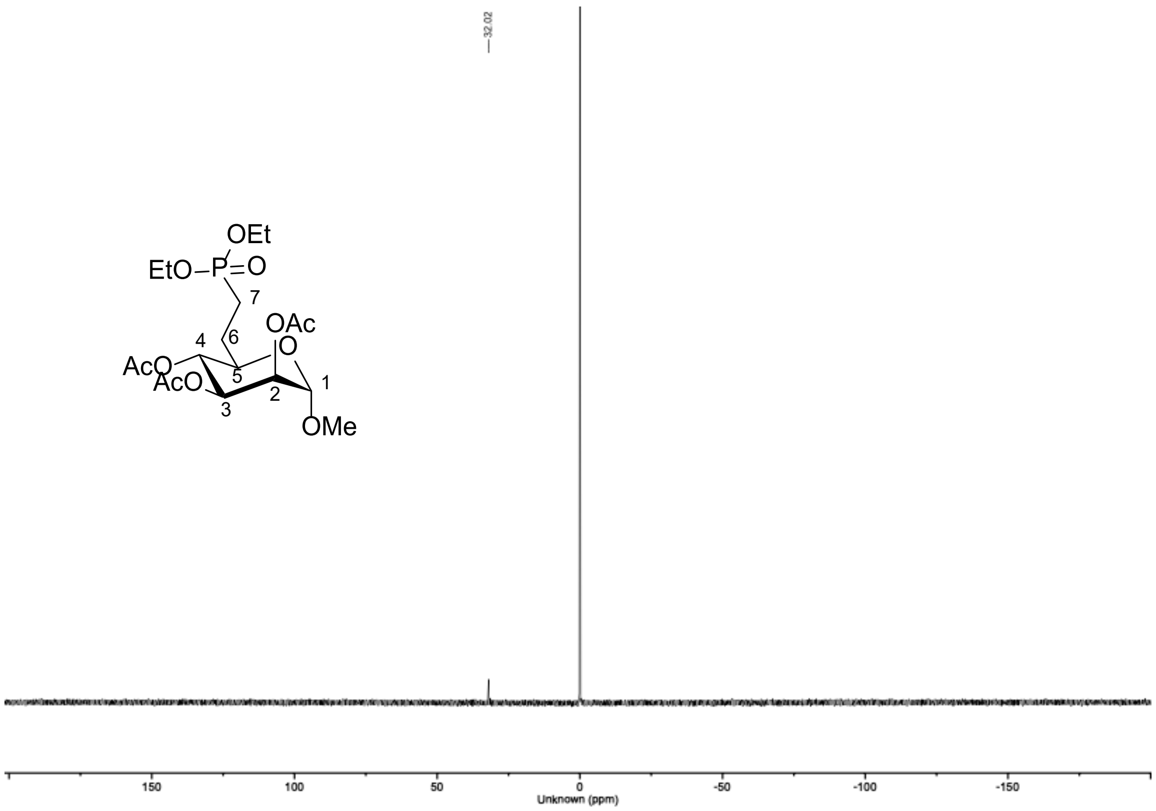
32.02
OEt
EtO–P=O
7
6
OAc
4
O
AcO
5
1
AcO
2
3
OMe
150
100
50
0
-50
-100
-150
Unknown (ppm)

3-(2,3,4-Tri-*O*-acetyl-6-deoxy-6-(diethylphosphonomethyl)-α-D-mannopyranosyl)prop-1-ene (XIV)

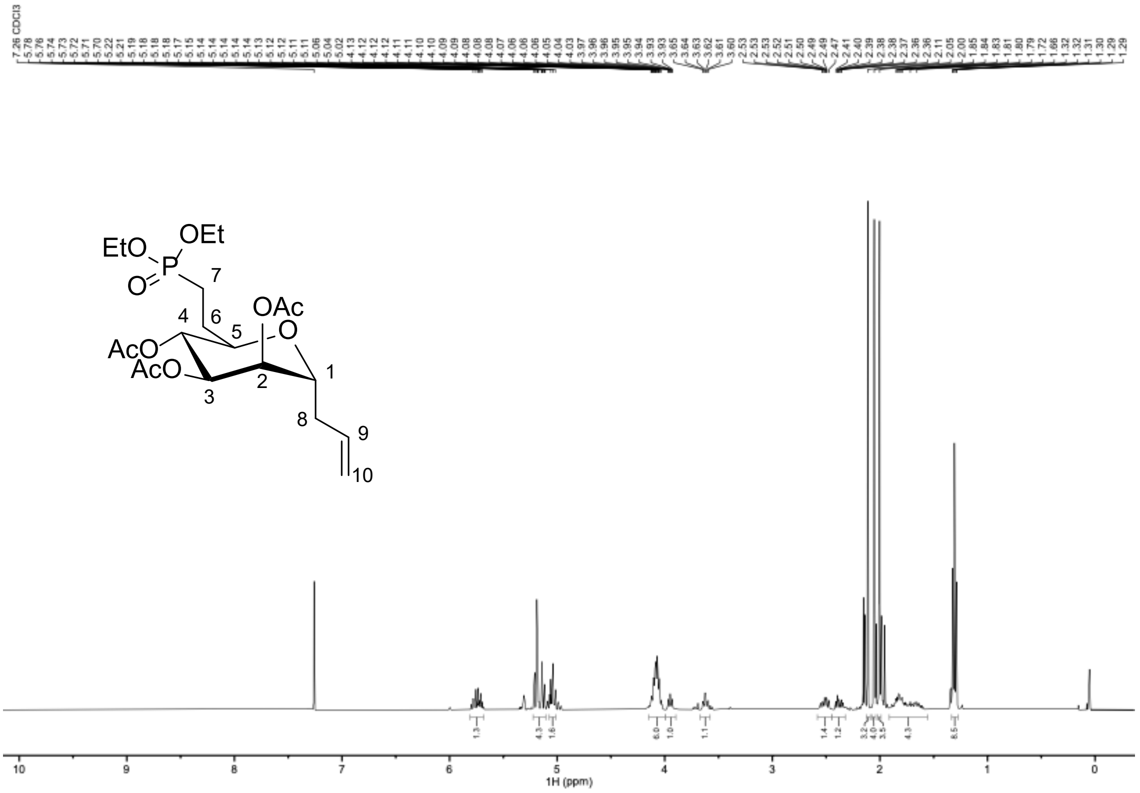

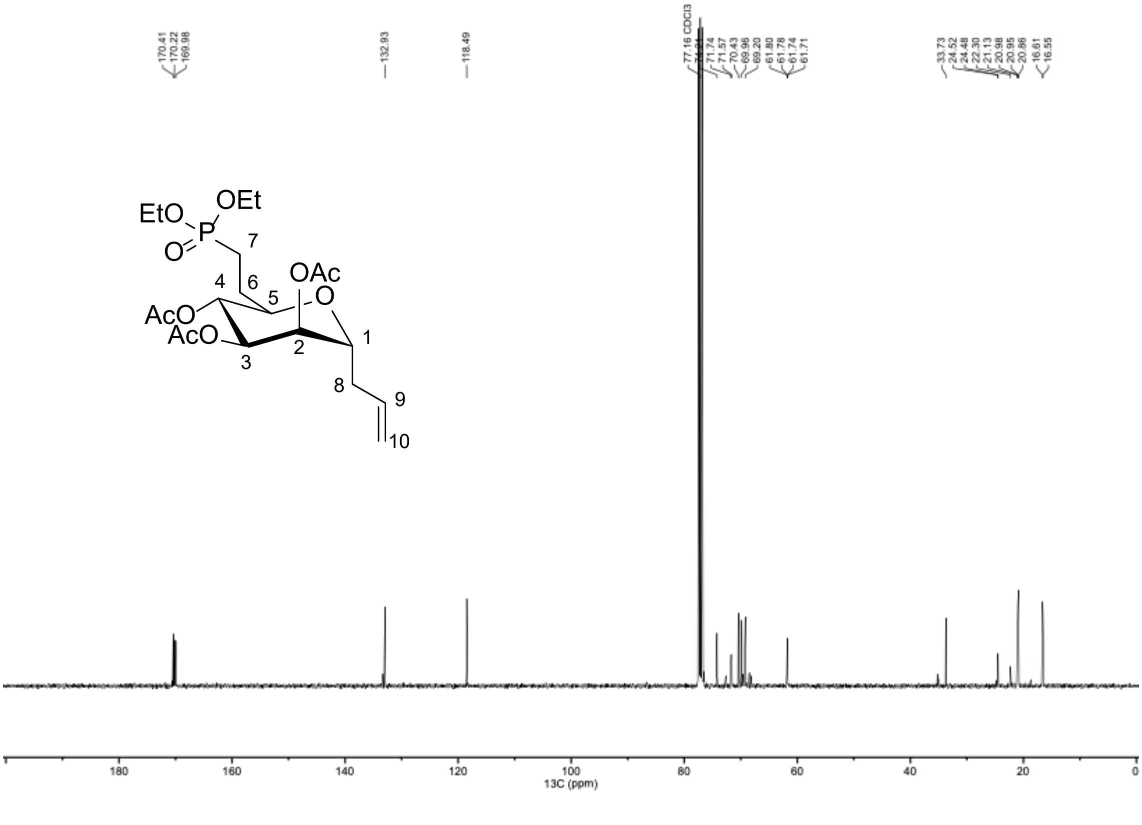

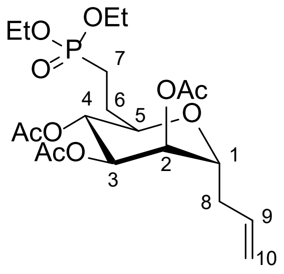
EtO
OEt
P
O
7
6
4
5
OAc
O
AcO
AcO
3
2
1
8
9
10

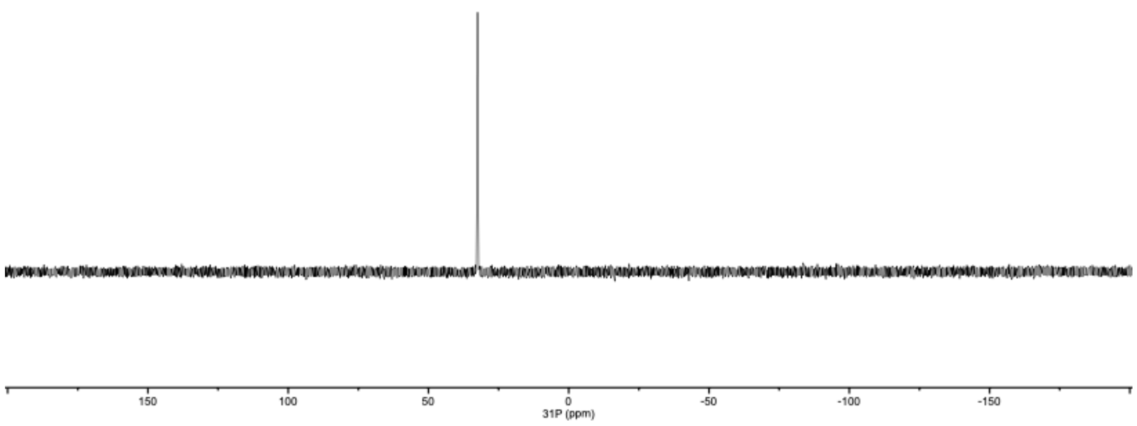
32.50
150
100
50
0
-50
-100
-150
31P (ppm)

(*E*)-4-(3-(6-Deoxy-6-phosphonomethyl-α-D-mannopyranosyl)-prop-1-en-1-yl)phenol bis(triethylammonium) salt (13)

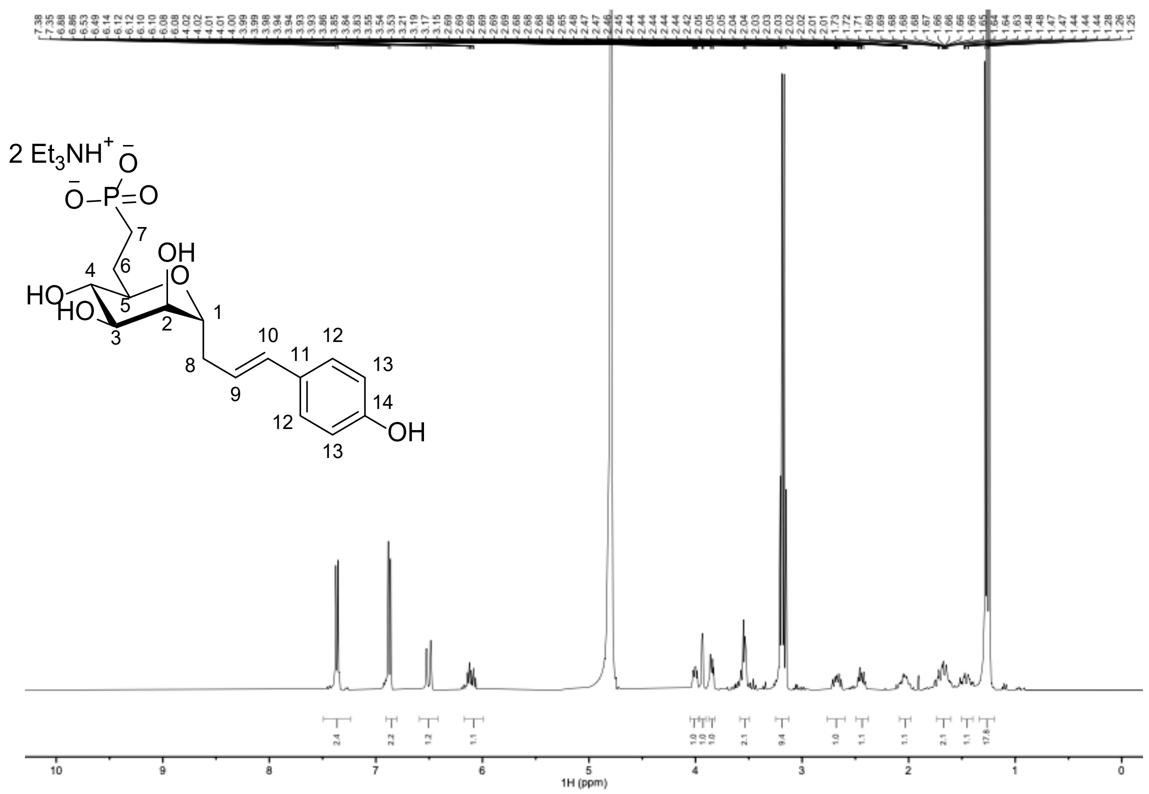

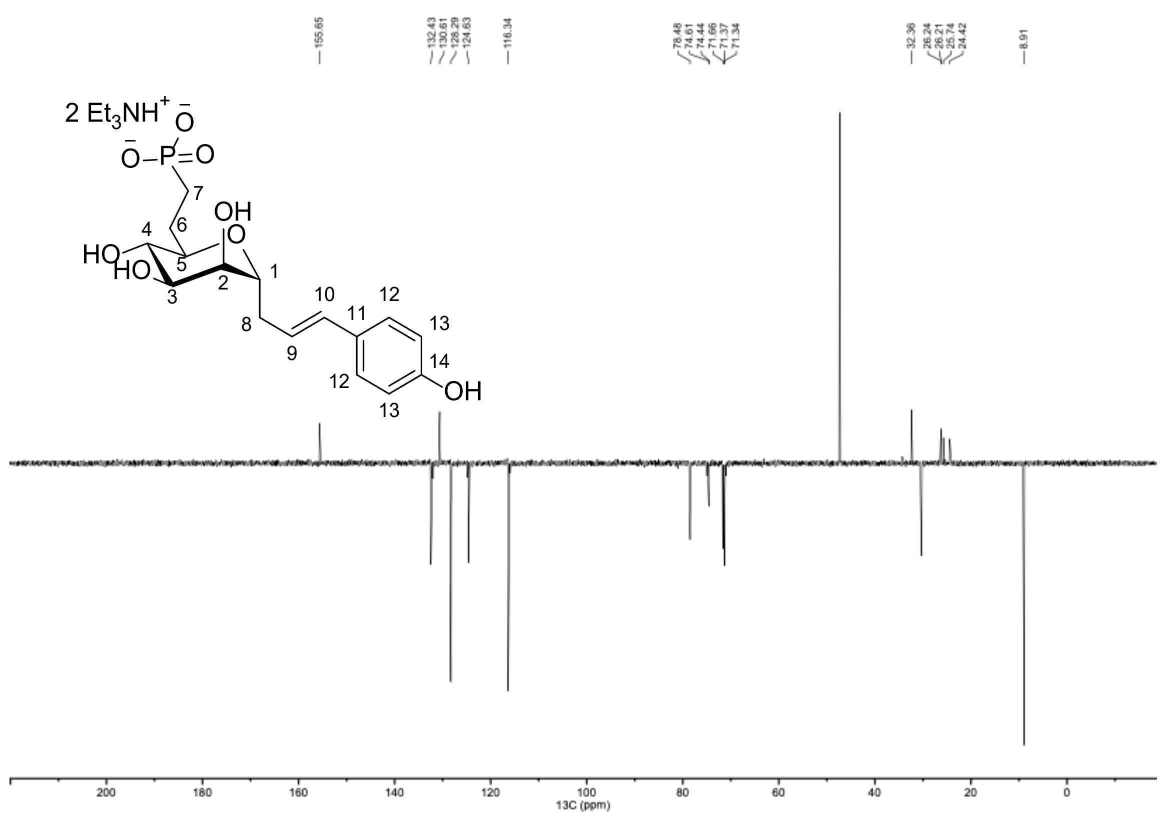

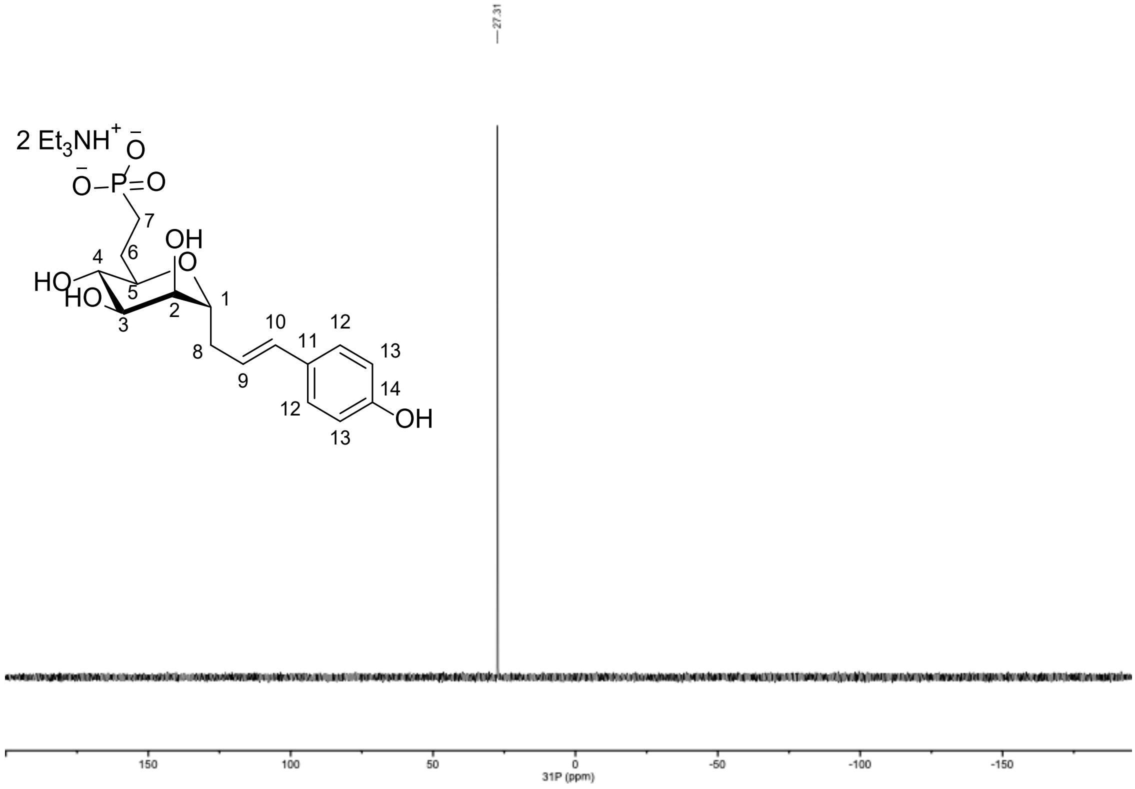

27.31
2 Et3NH+
OH
HO
HO
OH
150
100
50
0
-50
-100
-150
31P (ppm)

2-Bromoethyl (2,3,4-tri-*O*-acetyl-6-deoxy-6-(diethylphosphonomethyl)-α-D-mannopyranoside (F)

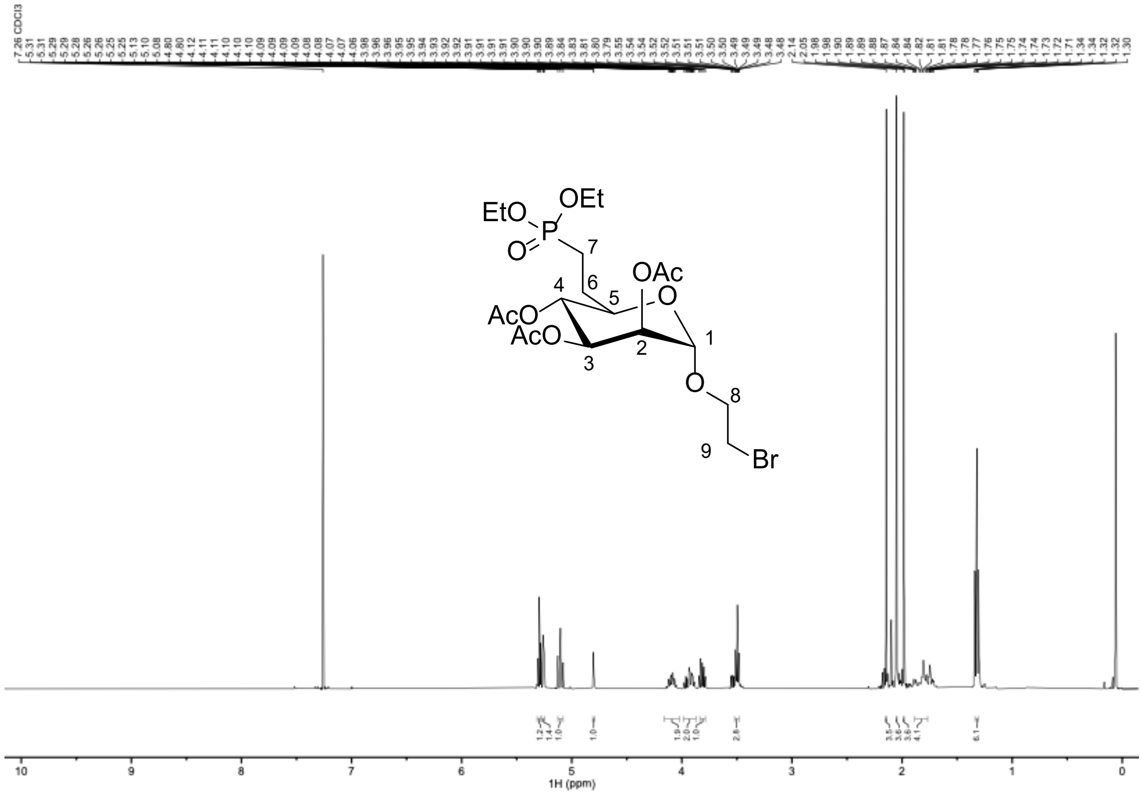

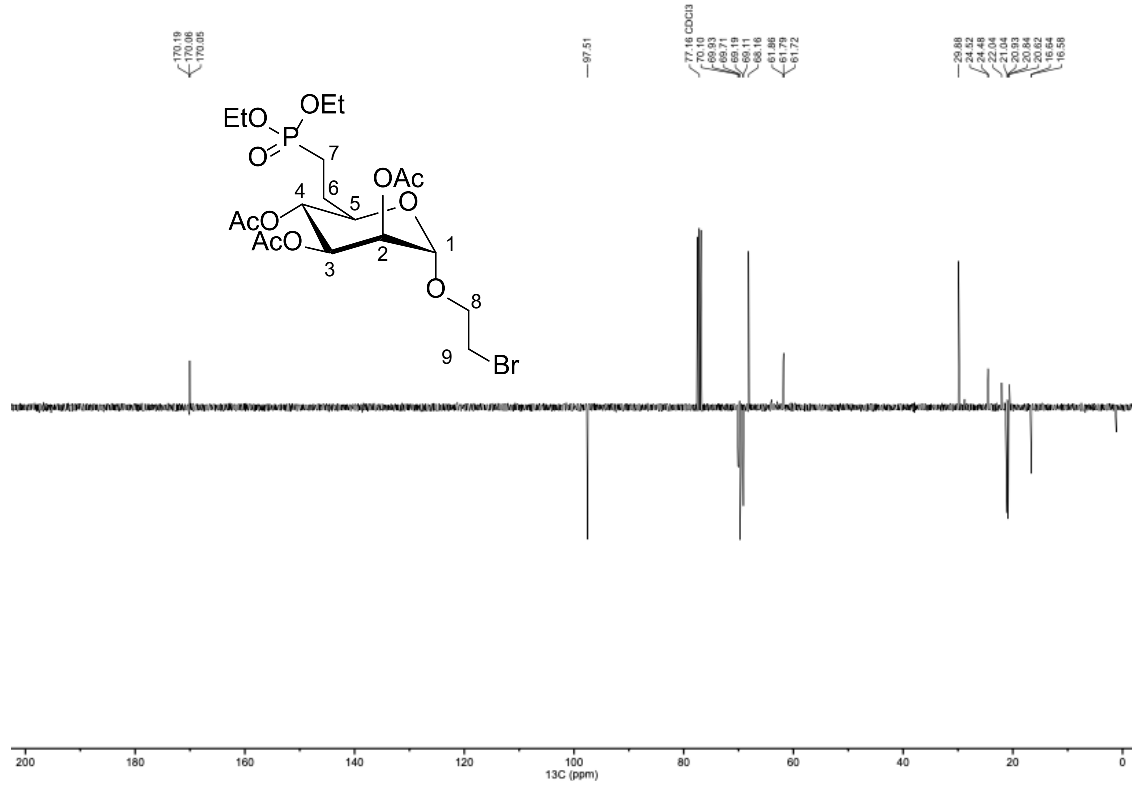

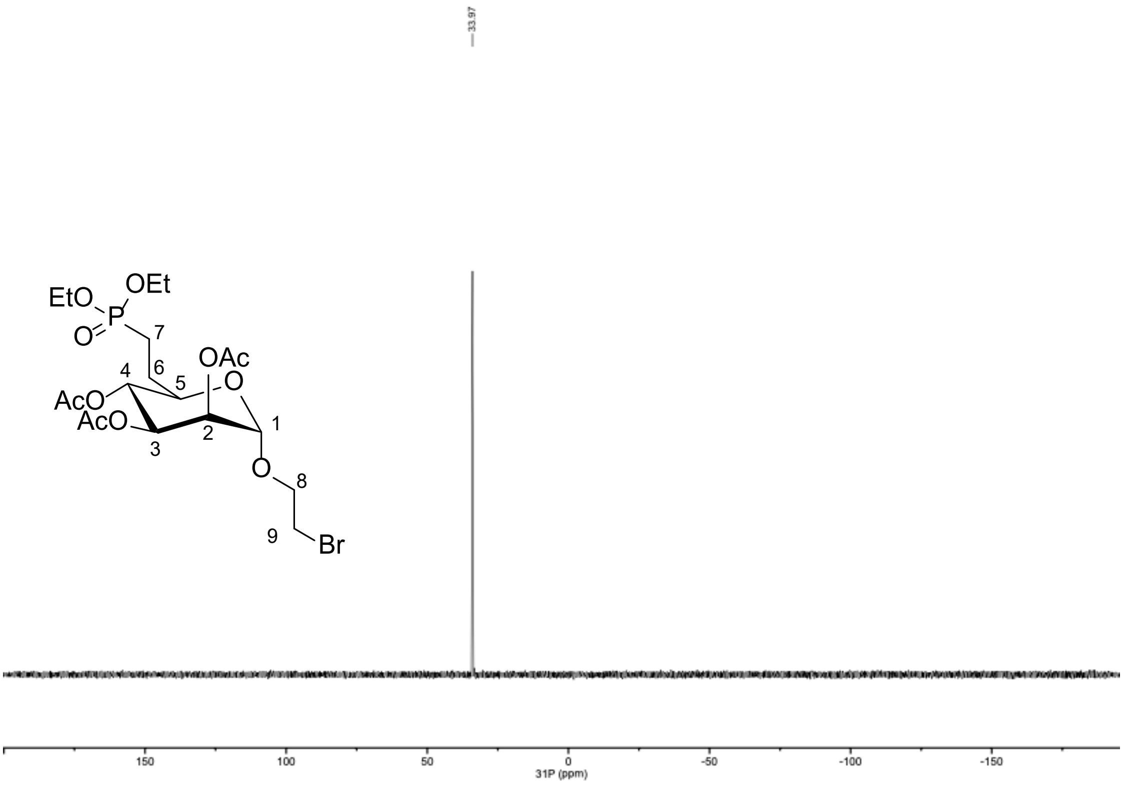
33.97
EtO
OEt
P
O
7
6
5
4
OAc
O
AcO
AcO
3
2
1
O
8
9
Br
150
100
50
0
-50
-100
-150
31P (ppm)

2-Azidoethyl (2,3,4-tri-*O*-acetyl-6-deoxy-6-(diethylphosphonomethyl)-α-D-mannopyranoside (XVI)

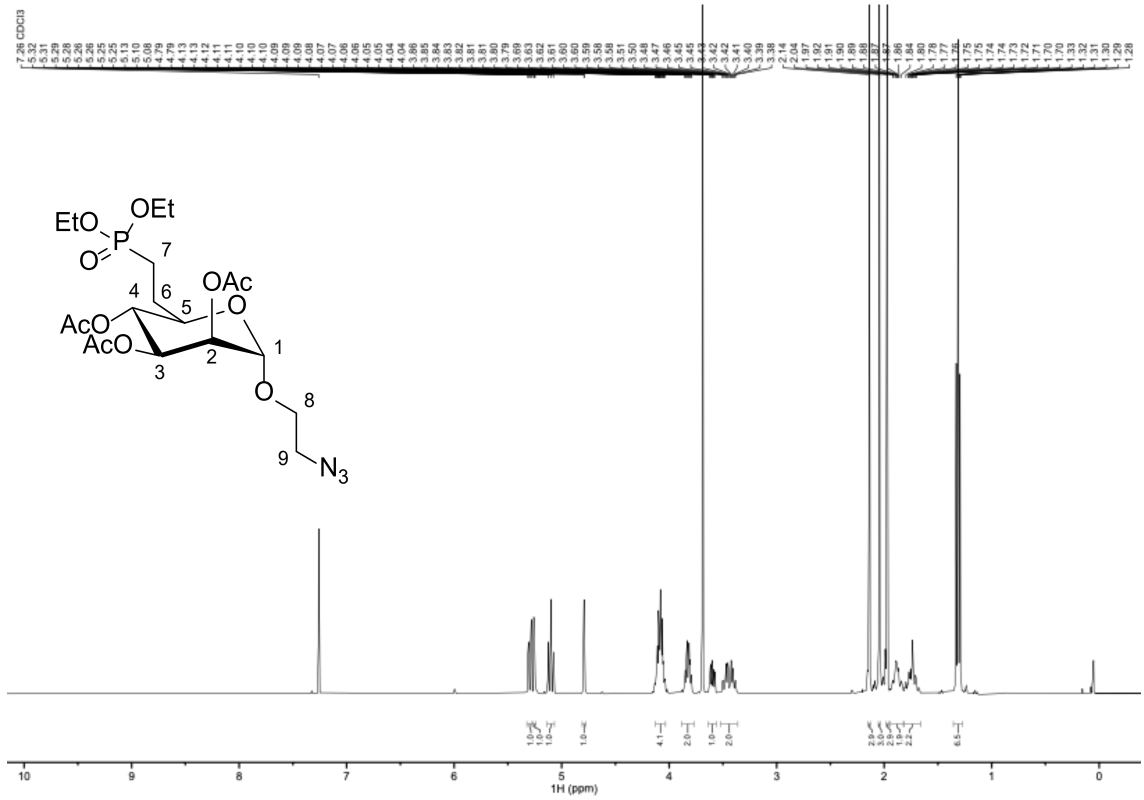

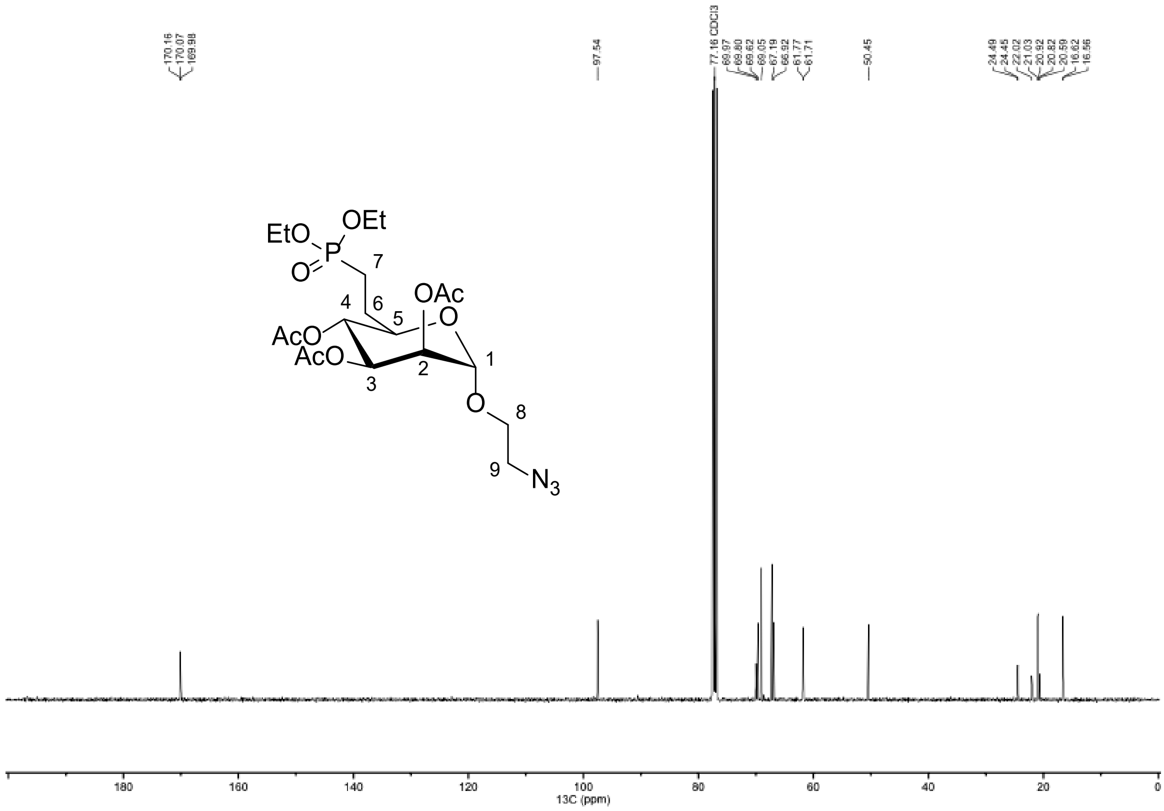

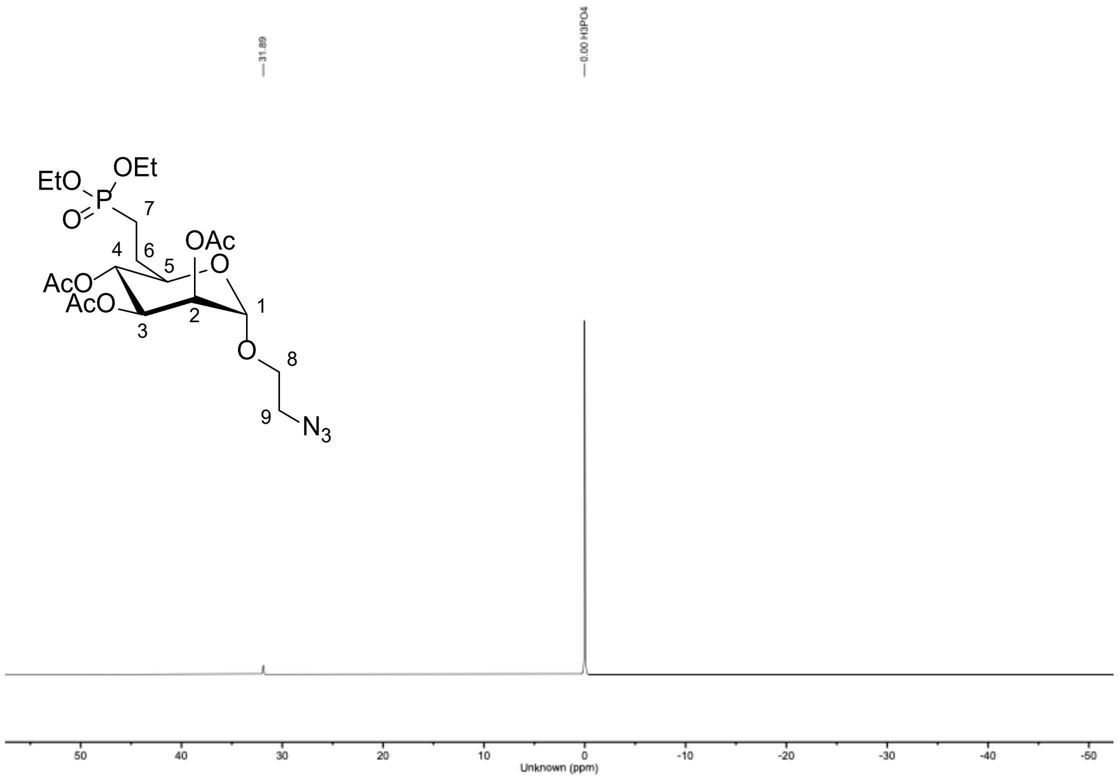
31.89
0.00 H3PO4
EtO
OEt
P
O
7
6
5
4
OAc
O
AcO
AcO
3
2
1
O
8
9
N3
50
40
30
20
10
0
-10
-20
-30
-40
-50
Unknown (ppm)

2-Azidoethyl (6-deoxy-6-phosphonomethyl-α-D-mannopyranoside disodium salt (14)

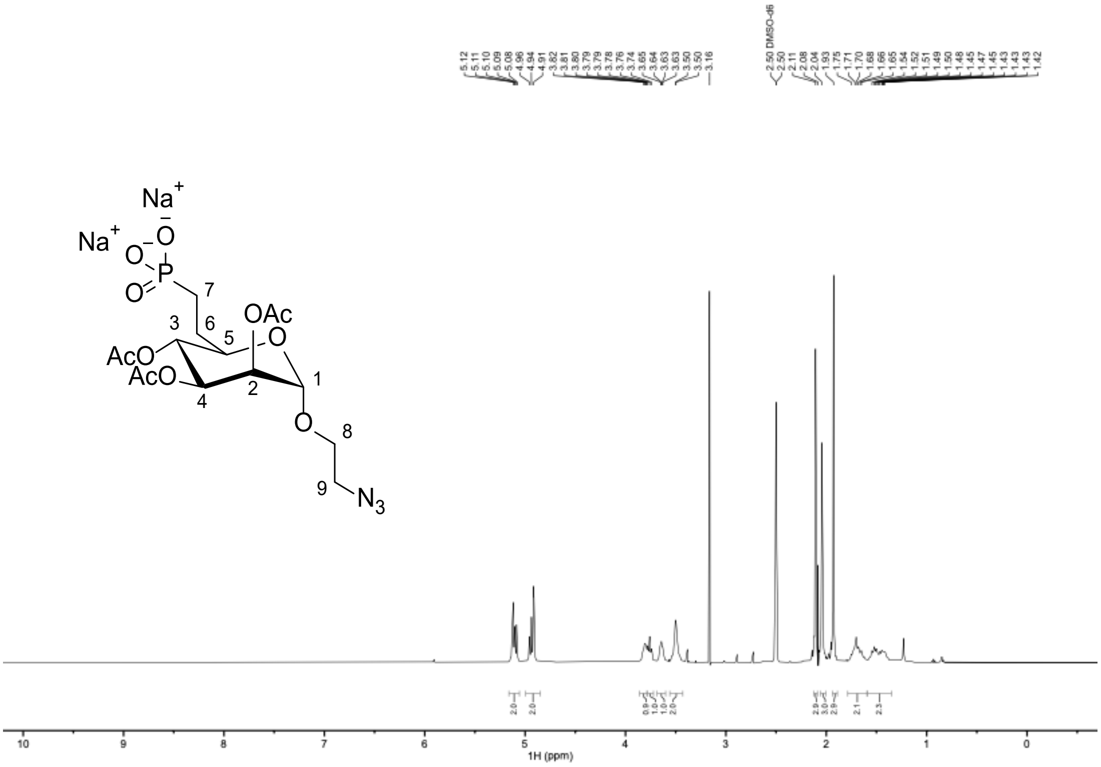

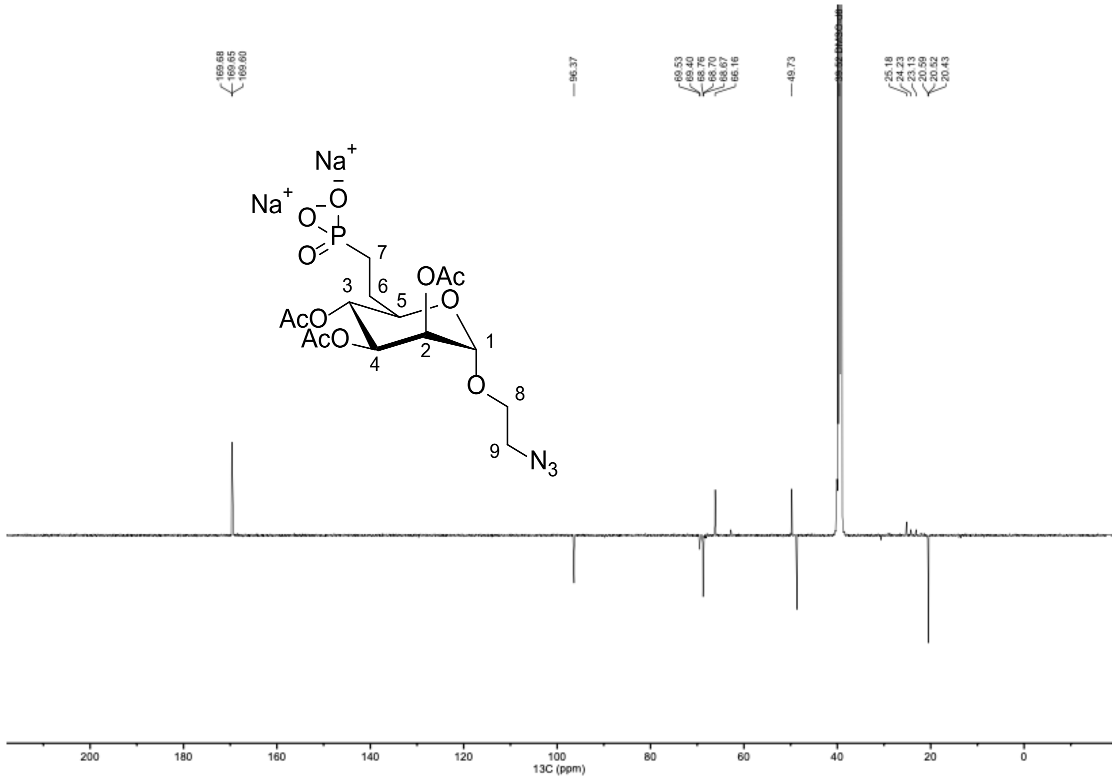

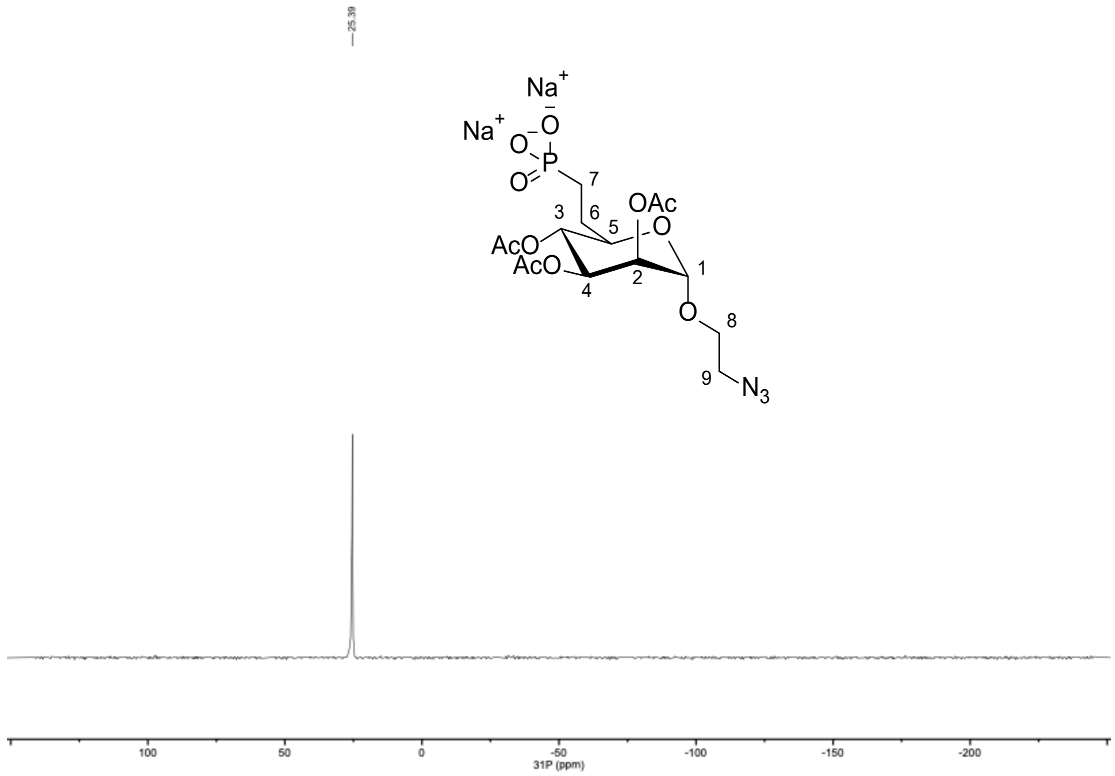
25.39
Na+
Na+
O
O
P
O
7
6
3
5
OAc
O
AcO
AcO
4
2
1
O
8
9
N3
100
50
0
-50
-100
-150
-200
31P (ppm)

Anisotropy measurements with glycopeptide **1** and CI-M6P/IGF2R

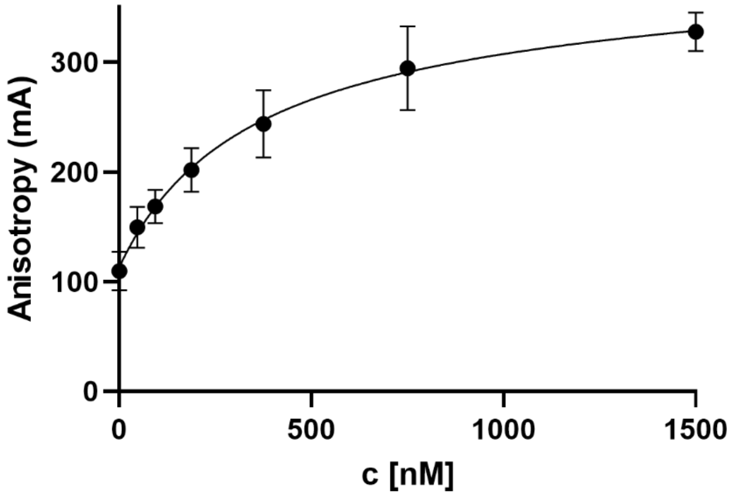

Figure S20. The non-subtracted reverse saturation binding of fluorescent glycopeptide **1** (10 nM) to soluble CI-M6P/IGF2R:D1-D15 determined by fluorescence polarization anisotropy measurements.

References

[6] A. J. Bochel, C. Williams, A. J. McCoy, H.-J. Hoppe, A. J. Winter, R. D. Nicholls, K. Harlos, E. Y. Jones, I. Berger, A. B. Hassan, M. P. Crump, *Structure* **2020**, *28*, 1300–1312.

[21] L. Schrödinger, Version 3, Schrödinger, LLC, New York, NY, **2024**.

[22] a) K. J. Bowers, E. Chow, H. Xu, R. O. Dror, M. P. Eastwood, B. A. Gregersen, J. L. Klepeis, I. Kolossváry, M. A. Moraes, F. D. Sacerdoti, J. K. Salmon, Y. Shan, D. E. Shaw, in *Proceedings of the 2006 ACM/IEEE Conference on Supercomputing*, ACM Press, New York, Tampa, FL, **2006**; b) D. E. S. Research, Maestro-Desmond Interoperability Tools; Schrödinger, New York, NY, **2024**.

[23] J. J. P. Stewart, MOPAC, Stewart Computational Chemistry, Colorado Springs, CO, USA, **2024**.

[24] R. A. Friesner, J. L. Banks, R. B. Murphy, T. A. Halgren, J. J. Klicic, D. T. Mainz, M. P. Repasky, E. H. Knoll, M. Shelley, J. K. Perry, D. E. Shaw, P. Francis, P. S. Shenkin, *J. Med. Chem.* **2004**, *47*, 1739– 1749.

[25] L. J. Olson, F. C. Peterson, A. Castonguay, N. M. Dahms, *Proc. Natl. Acad. Sci. U. S. A.* **2010**, *107*, 12493-12498.

[26] a) M. P. Jacobson, D. L. Pincus, C. S. Rapp, T. J. F. Day, B. Honig, D. E. Shaw, R. A. Friesner, *Proteins: Struct. Funct. Bioinf.* **2004**, *55*, 351-367; b) L. Schrödinger, LigPrep, Schrödinger, LLC, New York, NY, **2024**.

[27] C. Lu, C. Wu, D. Ghoreishi, W. Chen, L. Wang, W. Damm, G. A. Ross, M. K. Dahlgren, E. Russell, C. D. Von Bargen, R. Abel, R. A. Friesner, E. D. Harder, *J. Chem. Theory Comput.* **2021**, *17*, 4291–4300.

[28] J. Choutka, J. Kaminský, E. Wang, K. Parkan, R. Pohl, *J. Chem. Inf. Model.* **2025**, *65*, 762–777.

[29] J. Řezáč, P. Hobza, *J. Chem. Theory Comput.* **2012**, *8*, 141– 151.

[30] A. Klamt, G. Schüürmann, *J. Chem. Soc., Perkin Trans.* **1993**, *5*, 799–805.

[31] K. Kříž, J. Řezáč, *J. Chem. Inf. Model.* **2019**, *59*, 229– 235.

[32] d) V. Barragan-Montero, A. Awwad, S. Combemale, P. de Santa Barbara, B. Jover, J.-P. Molès, J.-L. Montero, *Chemmedchem* **2011**, *6*, 1771-1774; e) C. Vidil, A. Morère, M. Garcia, V. Barragan, B. Hamdaoui, H. Rochefort, J.-L. Montero, *European Journal of Organic Chemistry* **1999**, *1999*, 447-450; f) C. Ionescu, S. Sippelli, L. Toupet, V. Barragan-Montero, *Bioorg Med Chem Lett* **2016**, *26*, 636-639;

[33] V. Cachatra, A. Martins, M. C. Oliveira, M. C. Oliveira, L. Gano, A. Paulo, Ó. López, J. G. Fernández-Bolaños, M. Contino, N. A. Colabufo, D. Evans, T. Man, A. P. Rauter, *Organic & Biomolecular Chemistry* **2025**.

[38] D. Lee, M. S. Taylor, *Journal of the American Chemical Society* **2011**, *133*, 3724-3727.

[39] M. Kleban, U. Kautz, J. Greul, P. Hilgers, R. Kugler, H.-Q. Dong, V. Jäger, *Synthesis* **2000**, *2000*, 1027-1033.

[40] M. H. D. Postema, D. Calimente, L. Liu, T. L. Behrmann, *The Journal of Organic Chemistry* **2000**, *65*, 6061-6068.

[41] L. J. Olson, N. M. Dahms, J.-J. P. Kim, *J. Biol. Chem.* **2004**, *279*, 34000-34009.

[42] R. C. Johnston, K. Yao, Z. Kaplan, M. Chelliah, K. Leswing, S. Seekins, S. Watts, D. Calkins, J. Chief Elk, S. V. Jerome, M. P. Repasky, J. C. Shelley, *J. Chem. Theory Comput.* **2023**, *19*, 2380– 2388.
[43] L. Schrödinger, Prime, Schrödinger, LLC, New York, NY, **2024**.
[44] J. Li, R. Abel, K. Zhu, Y. Cao, S. Zhao, R. A. Friesner, *Proteins: Struct. Funct. Bioinf.* **2011**, *79*, 2794– 2812.
[45] W. L. Jorgensen, J. Chandrasekhar, J. D. Madura, R. W. Impey, M. L. Klein, *J. Chem. Phys.* **1983**, *79*, 926–935.
[46] a) L. Schrödinger, QSite, Schrödinger, LLC, New York, NY, **2024**; b) R. B. Murphy, D. M. Philipp, R. A. Friesner, *J. Comput. Chem.* **2000**, *21*, 1442-1457.
[47] G. J. Martyna, M. L. Klein, M. Tuckerman, *J. Chem. Phys.* **1992**, *97*, 2635– 2643.
[48] A. Y. Toukmaji, J. A. Board, Jr., *Comput. Phys. Commun.* **1996**, *95*, 73– 92.
[49] T. L. Bailey, J. Johnson, C. E. Grant, W. S. Noble, *Nucleic Acids Res.* **2015**, *43*, W39-W49.
[50] L. Holm, A. Laiho, P. Toronen, M. Salgado, *Prot. Sci.* **2023**, *23*, e4519.
